# Chemical structure of cichorinotoxin, a cyclic lipodepsipeptide that is produced by *Pseudomonas cichorii* and causes varnish spots on lettuce

**DOI:** 10.3762/bjoc.15.27

**Published:** 2019-02-01

**Authors:** Hidekazu Komatsu, Takashi Shirakawa, Takeo Uchiyama, Tsutomu Hoshino

**Affiliations:** 1 Department of Applied Biological Chemistry, Faculty of Agriculture and Graduate School of Science and Technology, Niigata University, Ikarashi 2-8050, Nishi-ku, Niigata 950-2181, Japan; 2 Department of Planning and General Administration, Institute of Vegetable and Floriculture Science, National Agriculture and Food Research Organization, 3-1-1 Kannondai, Tsukuba, Ibaraki 305-8519, Japan

**Keywords:** cichorinotoxin, lipodepsipeptide, necrotic lesion of lettuce, phytotoxin, *Pseudomonas cichorii*

## Abstract

*Pseudomonas cichorii*, which causes varnish spots on lettuce and seriously damages lettuce production during the summer season in the highland areas of Japan (e.g., Nagano and Iwate prefectures) was isolated. The structure of a toxin produced by this organism was analyzed based on the detailed evaluation of its 2D NMR and FABMS spectra, and this compound has not been reported previously. We propose the name cichorinotoxin for this toxin. In conjunction with the D or L configurations of each amino acid, which were determined by Marfey’s method, we propose the structure of cichorinotoxin to be as follows: 3-hydroxydecanoyl-(*Z*)-dhThr^1^-D-Pro^2^-D-Ala^3^-D-Ala^4^-D-Ala^5^-D-Val^6^-D-Ala^7^-(*Z*)-dhThr^8^-Ala^9^-Val^10^-D-Ile^11^-Ser^12^-Ala^13^-Val^14^-Ala^15^-Val^16^-(*Z*)-dhThr^17^-D-*allo*Thr^18^-Ala^19^-L-Dab^20^-Ser^21^-Val^22^, and an ester linkage is present between D-*allo*Thr^18^ and Val^22^ (dhThr: 2-aminobut-2-enoic acid; Dab: 2,4-diaminobutanoic acid). Thus, the toxin is a lipodepsipeptide with 22 amino acids. The mono- and tetraacetate derivatives and two alkaline hydrolysates, compounds **A** and **B**, were prepared. We discuss here the structure–activity relationships between the derivatives and their necrotic activities toward lettuce.

## Introduction

*Pseudomonas cichorii* causes varnish spots on lettuce. Varnish spots, also called midrib rot or bacterial rot [1–4], are dark brown and can induce necrotic lesions [1–7]. *P. cichorii*, isolated by us, infects a wide range of host plants, including monocot and dicot species (e.g., *Asteraceae, Solanaceae, Apiaceae,* and *Liliaceae*; 54 species in 17 families) [5], but necrotic lesions most commonly occur in lettuce plants and seriously hurt lettuce production in the highland areas of Japan (e.g., Nagano and Iwate prefectures) in the summer season [5]. Effective ways of inhibiting the infection are of great interest, but the infection mechanism remains unknown. We isolated a phytotoxin from the bacterial bodies of *P. cichorii* isolate YM8705 to clarify its mechanism of action. An injection of 750 pg of the isolated toxin caused necrotic lesions on lettuce midrib [5]. Furthermore, this toxin was confirmed to have an elicitor-like effect on the production of the phytoalexin lettucenin A [6–8]. As described below, the toxin was determined to be a cyclic lipodepsipeptide composed of a 3-hydroxydecanoic acid moiety and 22 amino acid residues. Two types of cyclic lipodepsipeptide phytotoxins are well known: relatively lower molecular weight compounds composed of 9 amino acids, such as those found in syringostatin [9], syringomycin [10–11] and syringotoxin [12], and higher molecular weight compounds consisting of 22 or 25 amino acids, which have been found in syringopeptins [13].

Herein, we report the structural determination of the toxin produced by *P. cichorii* YM8705, which was determined mainly from detailed analyses of its 2D NMR spectra. Although the structure is similar to that of syringopeptin 22A, composed of 22 amino acids, it is not identical, and this new phytotoxic compound has not been reported previously; we propose the name cichorinotoxin for this compound. Very recently, Huang et al. reported new phytotoxins, named cichopeptin A and B, from *P. cichorii* SF1-54 [14], which cause midrib rot disease in lettuce much like cichorinotoxin. However, cichorinotoxin is structurally distinct from the cichopeptins. The structural differences are as follows: four of the amino acid residues are different, and the lipophilic fatty acid also differs between the two toxins. Moreover, one of 22 amino acids was not definitively identified. Thus, cichopeptins A and B are different compounds compared to the cichorinotoxin isolated by us. The structure of cichopeptin proposed by Huang et al. was determined mainly by MS/MS spectral analyses, and the stereochemistry (D, L) of each of the amino acids was not determined. Herein, we present the almost complete assignments of all the ^1^H and ^13^C NMR signals of cichorinotoxin, and we determined the stereochemistry of 12 out of the 22 amino acids. In addition, we prepared chemically modified derivatives of cichorinotoxin and evaluated the abilities of some derivatives to cause lettuce rot, in order to provide insight into which structural units have a crucial role for causing the phytotoxic activity.

## Results and Discussion

### Composition of cichorinotoxin: 3-hydroxydecanoic acid and 22 amino acids

The culture conditions of *P. cichorii* YM8705 and the isolation procedure are described in the Experimental section. This toxin was isolated as a white solid. Its IR spectrum (KBr tablet, Supporting Information File 1, Figure S1) showed strong and broad absorption bands at 1535 cm^−1^ and 1660 cm^−1^, indicating that cichorinotoxin has amide groups and that it is a peptide. In addition, a band at 1740 cm^−1^ (medium) was also observed, suggesting that an ester bond is present. This toxin was subjected to Edman degradation, but this technique provided no information about the composition of the amino acids, suggesting that the amino group at the N-terminus is modified or blocked. However, this toxin was positive for ninhydrin; thus, a basic amino functional group is likely present in cichorinotoxin. FABMS (positive mode; matrix: *m*-nitrobenzyl alcohol, NBA) showed signals at *m*/*z* 2069 [M + H]^+^ and *m*/*z* 2070 (isotope peak), and signals at *m*/*z* 2067 [M − H]^+^ and *m*/*z* 2068 (isotope peak) were observed in the negative mode (Supporting Information File 1, Figure S2). This finding demonstrates that the molecular ion of cichorinotoxin (M^+^) is *m*/*z* 2068. To clarify the amino acid composition, cichorinotoxin was hydrolyzed in 6 N HCl at 110 °C for 5 h, 10 h and 22 h, and analysis of the amino acids in the hydrolytic residues showed that Thr, Ser, Pro, Ala and Val were present in the structure of this toxin. To determine the stereochemistry (either D or L) of each amino acid, the hydrolytic residues were reacted with Marfey’s reagent (1-fluoro-2,4-dinitrophenyl-5-L-alaninamide, FDAA) [15] to generate diastereomers. The diastereomers were then separated by HPLC with a C_18_ column using a solvent system composed of CH_3_CN and triethylamine phosphate (pH 3.0, see Figures S5B–S5E, Supporting Information File 1). Marfey’s method revealed the presence of 2,4-diaminobutanoic acid (Dab) and Ile in the toxin (Figures S5B and S5D). Four diastereomers are present in both Thr and Ile (structures, see Figure S5A, Supporting Information File 1): L-*allo*-type; L-*threo*-type; D-*threo*-type; and D-*allo*-type. The four diastereomers (FDAA derivatives) of Thr were well separated by HPLC (Supporting Information File 1, Figure S5C). In contrast, in the case of the Ile derivatives, separations between the L-*allo*- and L-*threo*-types and between the D-*allo*- and D-*threo*-types were unsuccessful, but separations between the L-*allo*- and D-*allo*-Ile and between the L-*threo*- and D-*threo*-Ile were achieved (Supporting Information File 1, Figure S5E). Consequently, the HPLC analyses demonstrated that the Thr residue in the toxin was the D-*allo*-form with (2*R*,3*R*)-stereochemistry, and the Ile residue was either the D-*allo*-derivative, with (2*R*,3*S*)-stereochemistry, or the D-*threo*-form, with (2*R*,3*R*)-stereochemistry. Table 1 summarizes the composition and the stereochemistry of each amino acid. As described below, NMR analysis of cichorinotoxin confirmed that three units of dehydrothreonine (dhThr: 2-aminobut-2-enoic acid) were present in the toxin, and the *Z*-geometry of the double bond was determined by NOESY analysis (see Figure 6 and Supporting Information File 1, Figure S10F).

**Table 1:** The proportion of each enantiomer of each amino acid, which were determined by an amino acid analyzer and by the peak areas of the D- and L-amino acids as estimated by Marfey’s method.

	Ala	Val	Pro	Ser	D-*allo*Thr^a^	Dab^b^	Ile^c^

D	7.65	4.33	1.49	0.68	1.0	–	0.91
L	1.21	1.42	–	0.82	–	0.92	–

The composition ratios of each amino acid presented here were in good agreement with those of the overall structure of cichorinotoxin shown in Figure 6. ^a^D-*allo*Thr indicates (2*R*, 3*R*)-stereochemistry. ^b^Dab: 2,4-diaminobutanoic acid. ^c^The Ile residue is either the D-*allo*- or the D-*threo*-type and not the L-*allo*- or L-*threo*-type.

**Figure 1:**
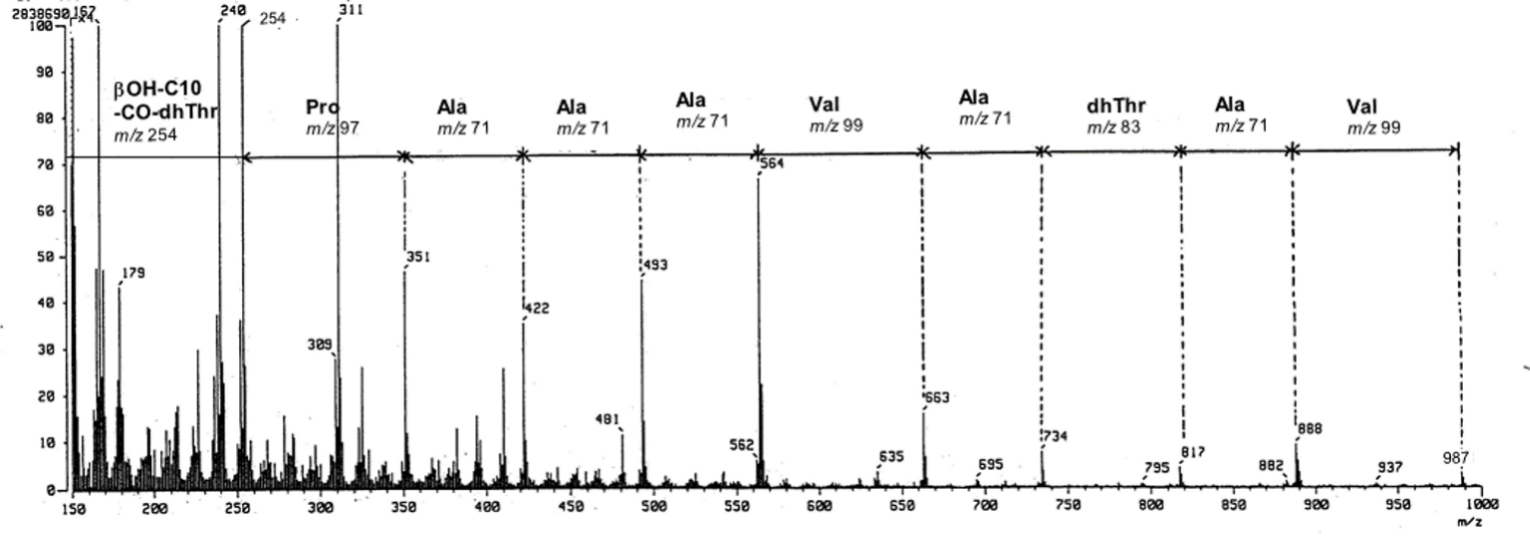
FABMS spectrum (positive mode, NBA matrix) of cichorinotoxin over the mass range of *m*/*z* 150─1000.

**Figure 2:**
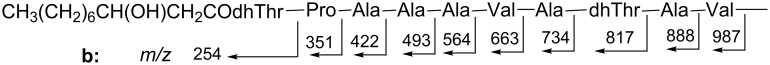
The segment identified based on the FABMS spectrum (peptide fragment **b** ions).

**Figure 3:**
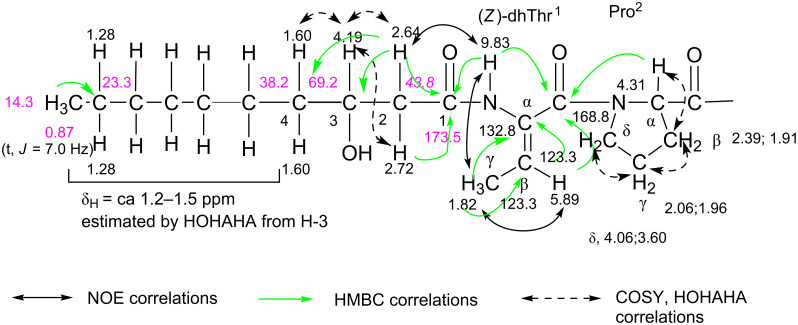
NMR analyses of the segment consisting of 3-hydroxydecanoyl-dehydrothreonin-proline (600 MHz, acetone-*d*_6_). DhThr^1^ and Pro^2^ indicate that the first and second amino acids in the peptide backbone are dhThr and Pro, respectively.

Analyses of the fragment ions in the FABMS spectrum of cichorinotoxin (Figure 1) showed the presence of the following peptide segment: Pro-Ala-Ala-Ala-Val-Ala-dhThr-Ala-Val (Figure 2). As described below, this toxin has a 3-hydroxydecanoic acid moiety as a fatty acid. Furthermore, the amino group at the N-terminus is modified or blocked, as mentioned above. Observation of a fragment ion of *m*/*z* 254 suggests that the 3-hydroxydecanoyl dehydrothreonine moiety (*m*/*z* 254) is linked to the Pro residue (Figure 2). This segment was further confirmed by detailed NMR analyses (Figure 3). To confirm the presence of this fatty acid moiety, cichorinotoxin (2.0 mg) was hydrolyzed at 120 °C for 90 min with 1.0 mL of 6 N aqueous HCl. EtOAc (1.5 mL) was added to the hydrolysate, and then the fatty acid was extracted. The extract was dried over anhydrous MgSO_4_ and concentrated in vacuo. A mixture (1.0 mL) of trimethylchlorosilane and *N*,*O*-bis(trimethylsilyl)acetamide (10:1) was added to the residue, the solution was heated at 80 °C for 24 h. The reaction mixture was then analyzed by GC–MS, which showed *m/z* 332 as the parent ion [M^+^], demonstrating that two TMS molecules were attached (Supporting Information File 1, Figure S3A). Commercially available (±)-3-hydroxydecanoic acid was also derivatized with the above mentioned TMS reagent (Supporting Information File 1, Figure S3B). The two products showed the same GC retention time and fragment ions in the MS, verifying that the fatty acid in cichorinotoxin is 3-hydroxydecanoic acid. As described above, a basic amino acid (positive in the ninhydrin test) is present in cichorinotoxin. To confirm the presence of a free amino group, cichorinotoxin (6.0 mg) was acetylated for 6 h at room temperature with Ac_2_O (1.5 mL) in a mixed solvent (5 mL) composed of CH_3_CN/pH 9.7 Na_2_CO_3/_NaHCO_3_ buffer solution (1:1). The FABMS spectrum (positive mode, NBA matrix, Supporting Information File 1, Figure S4A) showed a signal at *m*/*z* 2111 [M + H]^+^, indicating that the monoacetate was obtained; thus, the number of basic amino acids was limited to one. As shown in Table 1, Dab is the only basic amino acid present in the molecule. A prominent peak at *m*/*z* 2133 [M + Na: 2068 + 42 + 23]^+^ was observed in addition to the signal at *m/z* 2111 [M + H]^+^. In contrast, the reaction with Ac_2_O/Py showed a parent ion at *m*/*z* 2259 in the FABMS spectrum (*m*-NBA matrix, Supporting Information File 1, Figure S4B), which suggested that the tetraacetate was generated *m/z* [2068 + 42 × 4 + 23 = 2259], but the molecular ion of the tetraacetate peak (*m/z* 2237 [M + H]^+^) did not appear in the FABMS spectrum (Supporting Information File 1, Figure S4B). Consequently, these MS data of the mono- and tetraacetates, which were obtained under controlled reaction conditions, clearly indicated that one amino group (Dab) and three OH groups are present in the toxin. As shown in Table 1, cichorinotoxin has three OH groups in its structure due to the presence of two serine residues and one threonine residue. In addition, one 3-hydroxyfatty acid is present, as described above (Figure 2). Thus, there are four OH groups in cichorinotoxin. As mentioned below, the four acetate moieties were determined to be at Dab, the two serine residues and the β-hydroxyfatty acid based on NMR analyses. Thus, the OH group on the Thr residue is blocked through an ester linkage (IR, 1740 cm^–1^).

### NMR analyses to determine the overall structure of the monoacetate

**Figure 4:**
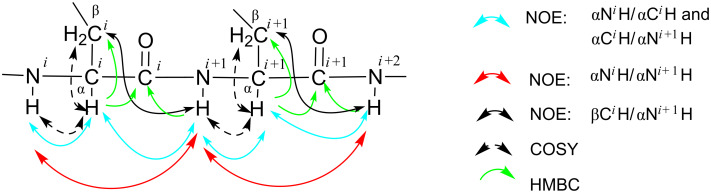
Key NMR observations used to construct the backbone sequence.

The ^1^H NMR spectrum of cichorinotoxin (not the acetate derivative) in DMSO-*d*_6_ (600 MHz) was not sufficiently resolved to elucidate the structure (Supporting Information File 1, Figure S6). However, the ^1^H NMR signals of the monoacetate were well separated (600 MHz, acetone-*d*_6,_ Supporting Information File 1, Figure S7). Therefore, the 1D and 2D NMR spectra of the monoacetate were analyzed to determine the overall structure. The ^1^H,^1^H COSY, TOCSY, NOESY, HMQC and HMBC spectra are shown in Supporting Information File 1, Figures S8–S12. The side chain of each amino acid (type of amino acid) was mainly determined from the ^1^H,^1^H COSY and TOCSY spectra. The NOESY spectrum provided important information on the backbone of the amino acid sequence, as depicted in Figure 4. The presence of a dhThr moiety in cichorinotoxin was confirmed as follows (see dhThr^1^ in Figure 3). The methyl signal (δ_H_ 1.82, d, *J* = 7.0 Hz, γC*H_3_*) had clear COSY cross peaks with the olefinic proton at δ_H_ 5.89 (q, *J* = 7.0 Hz, β*H*), which showed an HMQC cross peak with the carbon at δ_c_ 123.3 (d). In the HMBC spectrum, the γC*H_3_* moiety had clear correlations with the double bond carbons (δ_c_ 132.8, s, αC and δ_c_ 123.3, d, βC), and the β*H* exhibited clear HMBC correlations with the αC and carbonyl carbon (δ_c_ 168.8, s, *C*O). These NMR data clearly indicate the presence of a dhThr moiety (Figure 3), and the strong NOE between the γC*H_3_* protons and the αN*H* proton demonstrated that the double bond is in the *Z*-configuration. The αN*H* proton of dhThr showed a definitive HMBC cross peak with the carbonyl carbon (δ_c_ 173.5, s, C-1) of the fatty acid (FA) and showed NOEs with H-2 (δ_H_ 2.64, m; 2.72, m) of the FA, which exhibited HMBC correlations with C-1, the alcoholic carbon (δ_c_ 69.3, d, C-3) and C-4 (δ_c_ 38.2, t) of the FA, demonstrating that the 3-hydroxydecanoic acid (the FA) is directly connected to the dhThr moiety via an amide bond. This structural unit (Figure 3) was further confirmed by the *m*/*z* 254 ion in the FABMS spectrum of natural cichorinotoxin (Figure 1). We then determined the presence of three dhThr residues in the cichorinotoxin (Figure 6) by analyzing the NMR data; the βC*H* protons (olefinic protons) of the three dhThr moieties are found at δ_H_ 6.37 (q, *J* = 7.0 Hz), δ_H_ 6.24 (q, *J* = 7.0 Hz) and δ_H_ 5.89 (q, *J* = 7.0 Hz) in the ^1^H NMR spectrum of the monoacetate (Supporting Information File 1, Figure S7). In the NMR spectra of the monoacetate, α*H* (δ_H_ 4.31) of Pro^2^ showed a clear HMBC cross peak with the carbonyl carbon (*C*O, δ_c_ 168.8, s) of dhThr^1^, indicating that Pro is the 2nd amino acid residue, as shown in Figure 3. The five-membered Pro skeleton was confirmed by the COSY and TOCSY spectra, as shown in Figure 3. The FA-dhThr-Pro fragment was further supported by the fragment ion at *m*/*z* 351 in the FABMS (Figure 1 and Figure 2). As described above, a basic amino acid (positive to the ninhydrin test) is present; thus, the amino group was acetylated under basic conditions to afford the monoacetate of cichorinotoxin (Figure 6). This basic amino acid was determined to be 2,4(α,γ)-diaminobutanoic acid (Dab-20) by detailed analyses of the NMR data. In the COSY and TOCSY spectra of the acetate, the αN*H* proton (δ_H_ 8.61) was correlated with αC*H* (δ_H_ 3.76), βC*H_2_* (δ_H_ 2.33; 2.08) and γC*H_2_* (δ_H_ 3.21). The γC*H_2_* protons had a clear HMBC cross peak with the acetyl carbonyl carbon (δ_c_ 170.5, s, *C*OCH_3_), as shown in Figure S12B (Supporting Information File 1). The spectrum shown in Figure S12B also indicated that the ester linkage (IR, 1740 cm^−1^) is between βC*H* of Thr-18 (δ_H_ 5.23, m) and *C*O of Val-22 (δ_c_ 170.5, s).

Detailed analyses of the 2D NMR data (^1^H,^1^H-COSY, TOCSY and NOESY) and ^1^H,^13^C correlations (HMQC and HMBC)) of the peptides and proteins [16–18], which are illustrated in Figure 4, allowed us to construct the complete backbone amino acid sequence of cichorinotoxin; the NOESY data are shown in Figures S10A–S10F (Supporting Information File 1), and the HMBC data are shown in Figures S12A–S12C. Figures S10B and S10C highlight the NOE correlations of αN*^i^*H/αN*^i^*^+1^H. Figures S10D and S10E mainly depict the NOE correlations of αN*^i^*H/αC*^i^*H and αC*^i^*H/αN*^i^*^+1^H. Figure S10F mainly highlights the NOEs of βC*^i^*H/αN*^i^*^+1^H. Distinct NOEs were observed between the γN*H* of Dab-20 and COC*H*_3_, further verifying that the γNH_2_ of Dab was acetylated. The HMBC spectrum (Supporting Information File 1, Figure S12C) established that Dab^20^ was acetylated; an HMBC cross peak between γC*H_2_* of Dab^20^ and *C*OCH_3_ was observed.

Figure 6 depicts the key NMR data. Tables S1 and S2 (Supporting Information File 1) summarize the ^1^H and ^13^C NMR data of the monoacetate. Based on these data, cichorinotoxin contains a lipophilic fatty acid and 22 amino acids, and the peptide part is cyclized through an ester linkage between two of the amino acid residues (Thr^18^ and Val^22^). Thus, cichorinotoxin is classified as a cyclic lipodepsipeptide.

**Figure 5:**
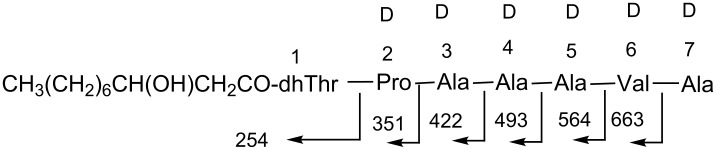
Fragment obtained by acid hydrolysis and each amino acid was exclusively D-configuration (peptide fragment **b** ions).

**Figure 6:**
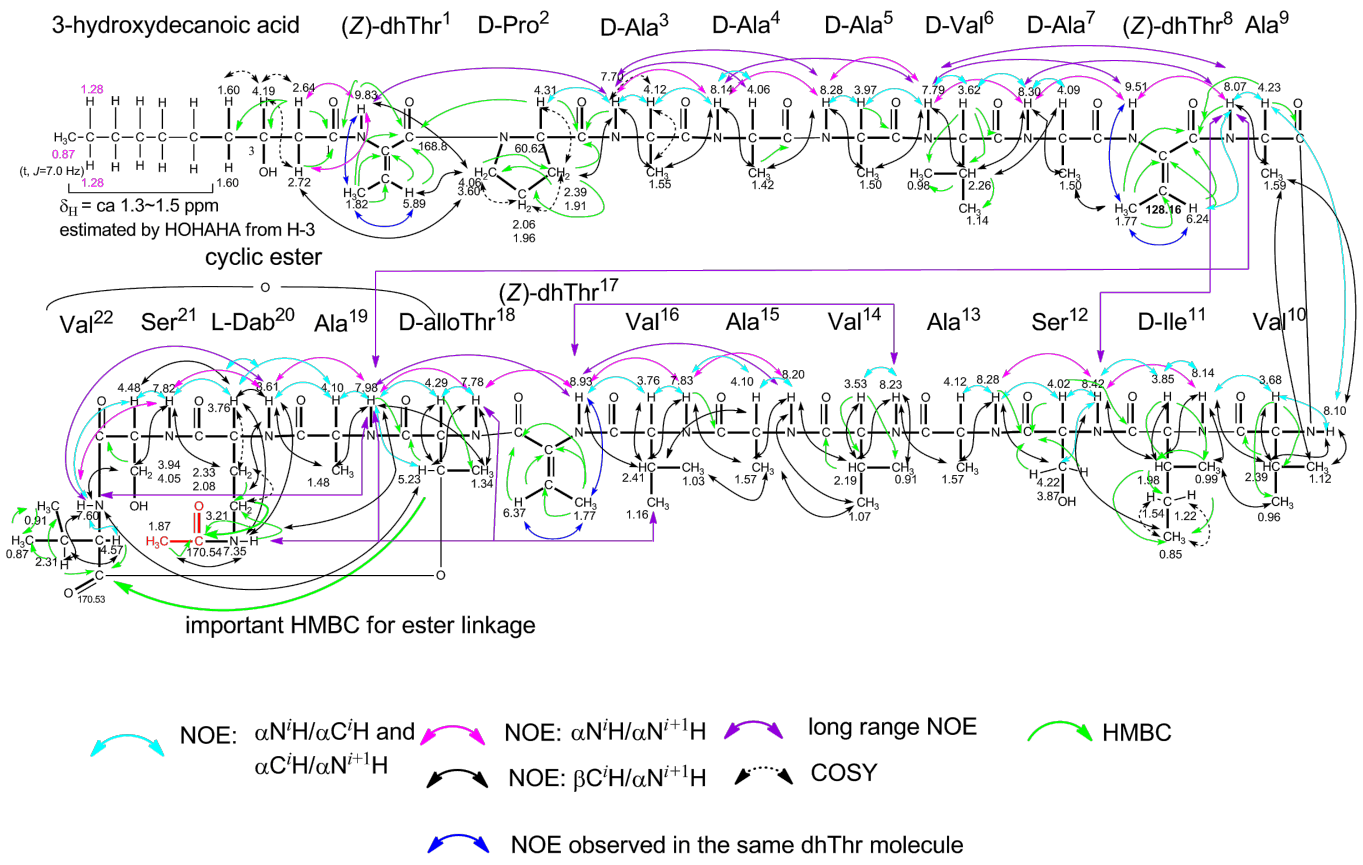
The complete structure of cichorinotoxin monoacetate and the assignments of ^1^H NMR chemical shifts, which were determined by various 2D NMR techniques.

To determine the stereochemistry of the amino acids (D or L), we tried to isolate partial fragments by acid hydrolysis. The toxin was dissolved in 12 N HCl and reacted for 20 h at room temperature. The partial hydrolysates were subjected to reversed-phase HPLC (C_18_), which showed that 6 main peptide fragments were produced. These fragments were purified and analyzed by FABMS. The FABMS spectrum of one of the fragments indicated that the segment had the structure shown in Figure 5. This fragment was subjected to acid hydrolysis according to the method described above. Marfey’s method revealed the following amino acid composition, including the stereochemistry: Pro (D × 1), Ala (D × 4) and Val (D × 1). None of the other generated peptide fragments were useful for determining stereochemistry. Considering the D or L configurations (Table 1), we could propose the stereochemistry of 12 of the amino acids (including the dhThr moieties), but the stereochemistries of the remaining 10 amino acids remain undetermined (see Figure 6).

### NMR data of the tetraacetate

**Figure 7:**
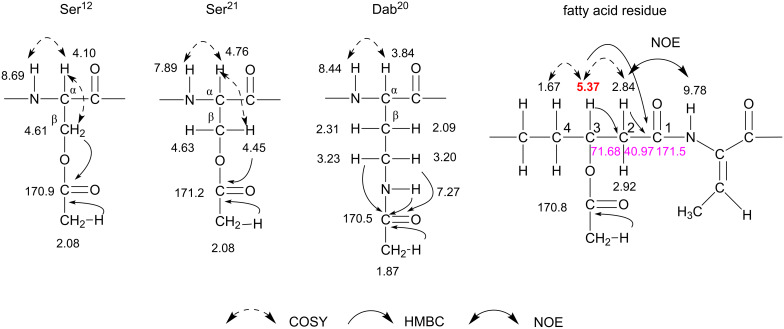
Determination of the positions of the acetyl groups in the tetraacetate.

To further confirm the structure of cichorinotoxin, the tetraacetate was analyzed by NMR (600 MHz, acetone-*d*_6_). The majority of the ^1^H NMR signals of the tetraacetate (Figure S13A, Supporting Information File 1) were separated, which was not the case in the spectrum of the monoacetate. The TOCSY, COSY and NOESY spectra of the tetraacetate are shown in Figures S13E–S13N (Supporting Information File 1). The NOE data indicated that the amino acid sequence of the tetraacetate is as shown in Figure S14 (Supporting Information File 1). The positions of the acetates were determined mainly from the HMBC and COSY spectra (Figure 7). The β-C*H_2_* protons of Ser^12^, Ser^21^ and Dab^20^ each had distinct HMBC correlations with one of the acetyl carbonyl carbons, the signals of which were confirmed by clear HMBC cross peaks for the acetyl methyl protons (see Figures S13B–S13D in Supporting Information File 1). Thus, the hydroxy groups of the two Ser residues were acetylated in the tetraacetate, and the amino group of Dab was acetylated in both the monoacetate and the tetraacetate. We found that the hydroxy group at C-3 of the FA residue was acetylated despite the H-3 proton not having a clear HMBC cross peak with the acetyl carbonyl carbon. The δ_H_ of H-3 in the tetraacetate was shifted downfield by ca. 1.0 ppm (acetylation shift) relative to that in the monoacetate (δ_H_ 4.19 → δ_H_ 5.37), confirming that the OH group of the FA was acetylated. Consequently, the complete structure of cichorinotoxin tetraacetate is shown in Figure S14 (Supporting Information File 1), and the fundamental scaffolds, composed of 3-hydroxydecanoic acid and the amino acid sequence backbone, of the mono- and tetraacetates were identical. Tables S3 and S4 (Supporting Information File 1) summarize the assignments of ^1^H and ^13^C NMR signal data.

### Compounds **A** and **B** generated by alkaline hydrolysis

**Figure 8:**
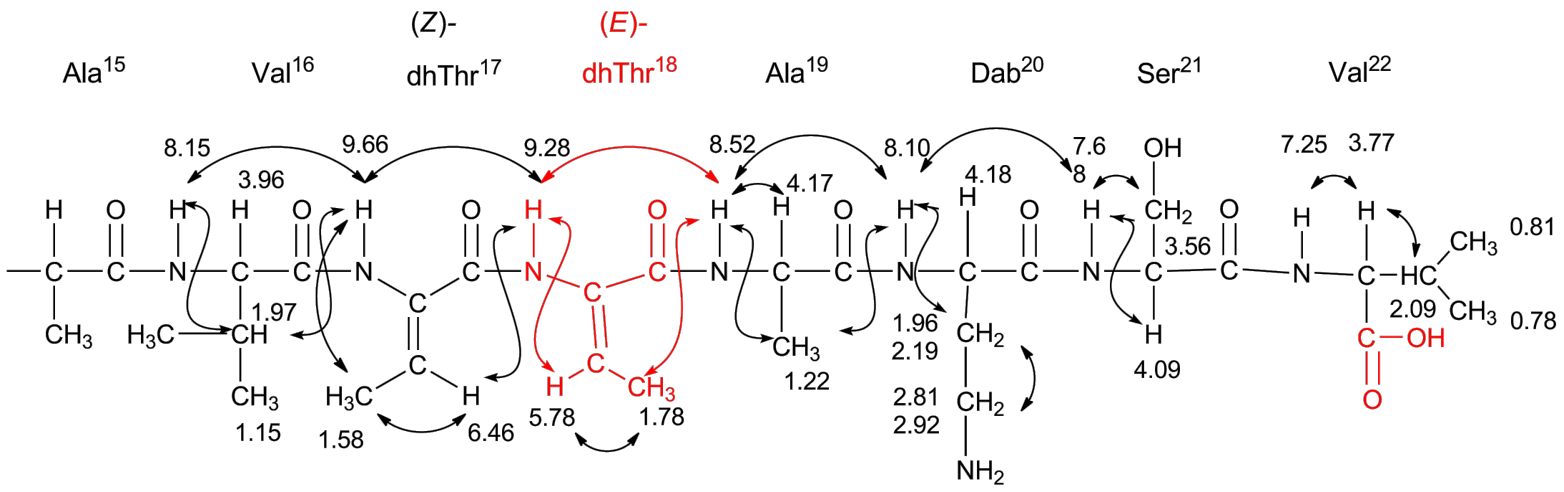
Partial structure of compound **A** as deduced from its NOE data; key NOEs are represented by double-headed arrows. The δ_H_ (ppm) are given, and these signals were measured in DMSO-*d*_6_ (600 MHz). The double bond of dhThr^18^ was in the *E*-configuration.

This toxin is composed of a cyclic peptide formed by an ester linkage between the OH of Thr^18^ and the CO_2_H of Val^22^. Figure S15 (Supporting Information File 1) shows the primary structures of cichopeptin A and B [14], syringopeptin 22-A and 22-B [13], corpeptin A and B [19], fuscopeptin [20] and tolaasin I [21–22]**,** which are typical lipodepsipeptides produced by *Pseudomonas* sp., and they are structural analogues of cichorinotoxin. All of these peptides have a lipophilic fatty acid moiety connected to dhThr^1^-D-Pro^2^, and the amino acids dhThr^17^ and *allo*Thr^18^ are strictly conserved. Moreover, the conserved *allo*Thr moiety is involved in the cyclic ester linkage. Therefore, the ester bond of natural cichorinotoxin was cleaved by alkaline hydrolysis (0.02 N KOH/MeOH) at room temperature for 5 h to examine the role of the cyclic structure in the ability to cause varnish spots on lettuce. Two products, **A** and **B**, were generated by the hydrolysis. The FABMS spectrum of compound **A** indicated that the MW of **A** was identical to that of unreacted cichorinotoxin (Figure S16A, Supporting Information File 1). As shown in Figure S16B, signals for four olefinic protons (βC*H* of dhThr) were observed in the ^1^H NMR spectrum (600 MHz, DMSO-*d*_6_) δ_H_ 6.46 (q, *J* = 6.6 Hz, 1H for dhThr^17^); 6.38 (q, *J* = 7.0 Hz, 1H for dhThr^8^); 5.78 (q, *J* = 7.2 Hz, 1H for dhThr^18^) and 5.58 (q, *J* = 6.8 Hz, 1H for dhThr^1^). This finding indicated that an additional dhThr residue was produced during the hydrolysis. Moreover, the NOE data indicated that the double bond in the newly formed dhThr was in the *E*-geometry (Figures S16C–16E), as shown in Figure 8. The NOE data (Figure 8), which reveal the amino acid sequence, verified that Thr^18^, which is connected to CO_2_H of Val^22^ via an ester bond, was changed into dhThr and that the backbone sequence from the fatty acid moiety to dhThr^17^ was identical to that of parent cichorinotoxin, but the ester bond was cleaved by the alkaline hydrolysis to yield the linear peptide, as shown in Figure 8 and Figure S18 (Supporting Information File 1).

**Figure 9:**
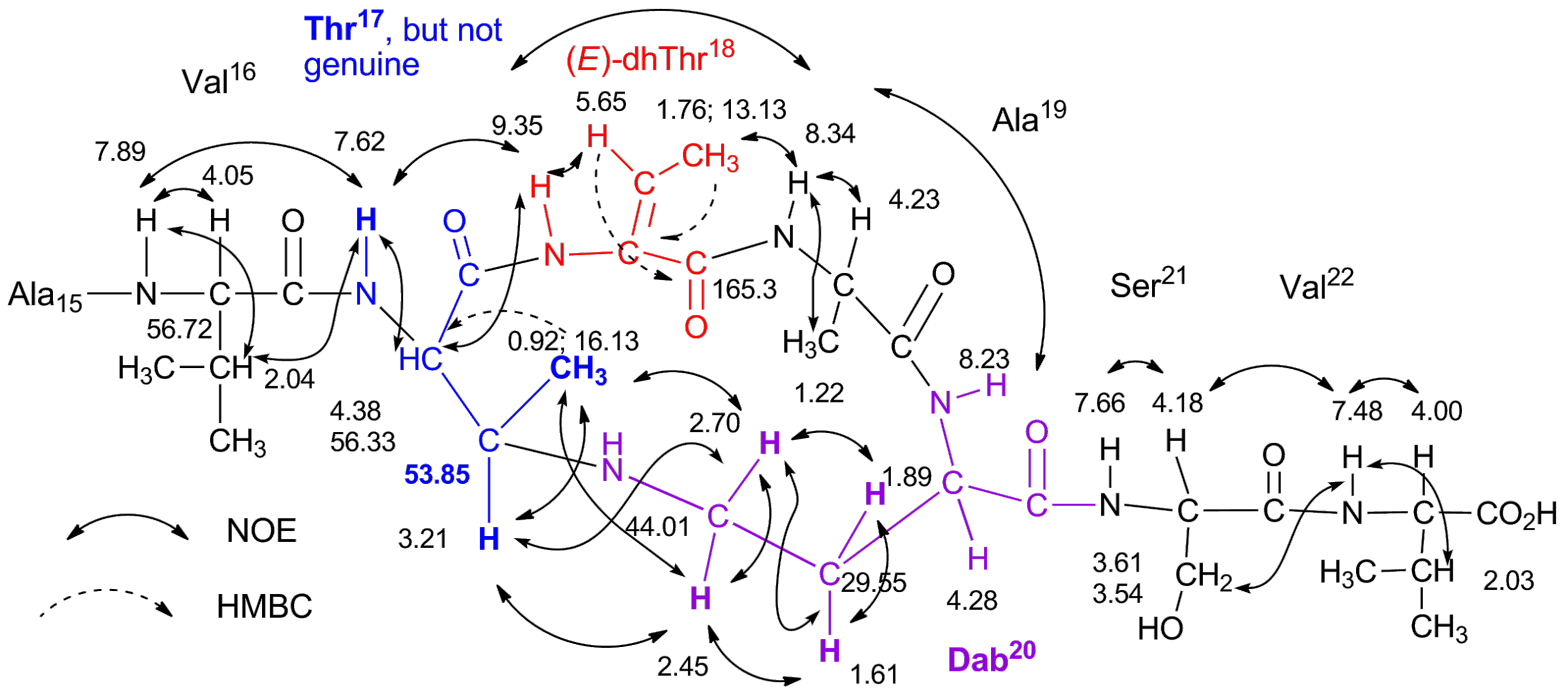
Partial structure of compound **B** (Val^16^ to Val^22^ residues). The chemical shifts (δ_H_ and δ_C_ (ppm)) are shown, and these values were obtained in DMSO-*d*_6_ (600 MHz NMR instrument).

On the other hand, compound **B** contained three olefinic protons (βC*H* of the dhThr residues) in its structure (Figure S17B), and their signals appeared at δ_H_ 6.37 (q, *J* = 7.0 Hz, 1H for dhThr^8^); 5.65 (q, *J* = 7.2 Hz, 1H for dhThr^18^); 5.59 (q, *J* = 7.0 Hz, 1H for dhThr^1^). The chemical shifts of the two olefinic protons of dhThr^8^ and dhThr^1^ were nearly the same as those of compound **A**. The NOESY spectrum, shown in Supporting Information File 1, Figure S17F, indicated that the double bonds of dhThr^8^ and dhThr^1^ were in the *Z*-configuration, which is identical to unreacted cichorinotoxin, but the *E*-geometry was found for the double bond of dhThr^18^, which is identical to that of compound **A**. The amino acid sequence of compound **B** was determined mainly from its NOESY spectrum (Supporting Information File 1, Figures S17C, D and E). The fatty acid moiety was retained, and the sequence from dhThr^1^ to Ala^16^ was the same as that of unreacted cichorinotoxin. As shown in Figure 9, the 17th residue was Thr-like amino acid, which was different from that (dhThr) in the parent cichorinotoxin and in compound **A**. The subsequent amino acids in the sequence (18th to 22nd) were identical to those in compound **A**. However, it is notable that the residue in the 17th position was not in fact genuine Thr because the chemical shift of β*C*H was *δ*_C_ (ppm) 53.85, which is significantly upfield compared with the resonance of this carbon in genuine Thr (approximately 67 ppm β*C*H-O function). Furthermore, distinct NOEs were observed between the γC*H* protons of Dab^20^ and the βC*H* protons of the 17th amino acid (Thr-like) and between the γC*H* protons of Dab^20^ and the γC*H_3_* protons of the 17th Thr-like amino acid. These data unambiguously indicated that the Thr-like residue was linked with Dab through the γ-nitrogen atom of Dab^20^, as shown in Figure 9.

**Figure 10:**
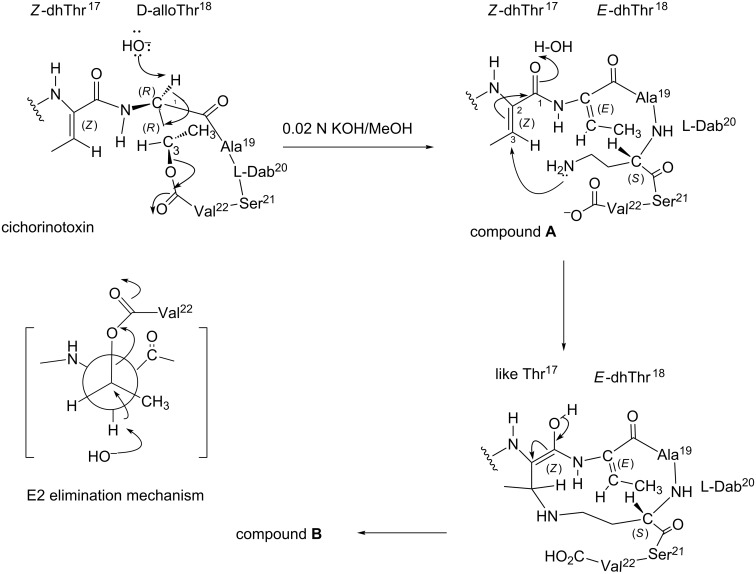
Mechanism of the formation of compounds **A** and **B** from cichorinotoxin by alkaline hydrolysis.

Consequently, the overall structures of compounds **A** and **B** are depicted in Figure S18 (Supporting Information File 1). Figure 10 shows the reaction mechanisms for generating compounds **A** and **B** from cichorinotoxin by treatment with dilute KOH. The proton at α-position of D-*allo*Thr^18^ was first eliminated by the base (OH^−^ ion), followed by the release of Val^22^-COO^−^ ion and the production of dhThr. This process follows an E2-type elimination mechanism (see a Newman projection), thus leading to compound **A**, which has an *E*-double bond for dhThr instead of a *Z*-double bond. Compound **A** was further converted into compound **B** by nucleophilic attack of the free amino group of Dab^20^ on the C-3 of dhThr^17^.

### Necrotic lesion on lettuce by cichorinotoxin derivatives (mono- and tetraacetates and compounds **A** and **B**)

Figure S19A (Supporting Information File 1) shows the bioassay method. Figure S19B indicates that the necrotic lesions caused by the monoacetate (3) were somewhat less severe, but the compound was still active; the tetraacetate (4) showed almost no activity. For the monoacetate (3), the hydrophilic NH_2_ of Dab^20^, which is one of the residues in the macrocycle of the peptide, is acetylated. The tetraacetate (4), in which the fatty acid, two serine residues (Ser^12^ and Ser^21^) and Dab^20^ are all acylated, had no activity. Ser^12^, which is present in the linear portion of the peptide, is not conserved in the toxins listed in Supporting Information File 1, Figure S15; thus, it is likely that the hydrophilic Ser^21^, embedded in the macrocycle of the peptide, is crucial for the necrosis activity. The peptides in the macrocycle, listed in Supporting Information File 1, Figure S15, consist of 5 or 8 amino acids, and of these, at least two of the hydrophilic amino acids are conserved; this could be the Dab-Ser or the Dab-Dab sequence for example. The conversion of the two hydrophilic amino acids into hydrophobic residues by acylation may have caused the complete loss of activity seen with the tetraacetate (4). Acyclic compound **A** (a linear lipopeptide) produced by alkaline treatment had no activity, supporting that a macrocyclic peptide would be essential for biological activity. The sequence *Z-*dhThr^17^-D-*allo*Thr^18^ of cichorinotoxin was converted to *Z*-dhThr^17^-*E*-dhThr^18^ by alkaline hydrolysis. This additional structural modification may have further decreased the activity. Compound **B** has a macrocycle composed of 4-amino acids, but it exhibited no necrotic activity. As seen in the structures of all the toxins depicted in Figure S15 (Supporting Information File 1), the macrocycles are in terminal positions; thus, having the macrocycle at the termini may be required for the toxic activity. At the present stage, the crucial structural units for the toxic activity cannot be specified and are still uncertain. Further studies are required.

## Conclusion

In summary, we succeeded in proposing the overall structure of cichorinotoxin, 3-hydroxydecanoyl-(*Z*)-dhThr^1^-D-Pro^2^-D-Ala^3^-D-Ala^4^-D-Ala^5^-D-Val^6^-D-Ala^7^-(*Z*)-dhThr^8^-Ala^9^-Val^10^-D-Ile^11^-Ser^12^-Ala^13^-Val^14^-Ala^15^-Val^16^-(*Z*)-dhThr^17^-D-*allo*Thr^18^-Ala^19^-L-Dab^20^-Ser-^21^-Val^22^, in which an ester linkage is formed between D-*allo*Thr^18^ and Val^22^. The configurations (D or L) of all 22 amino acids could not be determined in the present study. Authentic D-[1-^13^C]- and L-[1-^13^C]-Val were separately fed into cultures of *P. cichorii* YM8705, and the cichorinotoxin thus obtained was analyzed by ^13^C NMR spectroscopy. Five Val residues (four D-Val and one L-Val) are included in cichorinotoxin (Figure 6 and Table 1). All the five ^13^C signals of *C*O- of Val residues appeared with almost the same signal intensities in each of the ^13^C NMR spectra, which were obtained by the feedings of L- and D-Val (Figure S20B and S20C, Supporting Information File 1). This finding definitively demonstrated that an epimerase (racemase) is involved in the biosynthetic gene cluster. Differences in the ^13^C signal intensities were a little in the feeding experiments using L- or D-[1-^13^C]-Ala (see Figure S20D and S20E, Supporting Information File 1). These results indicated that an epimerase is involved in the biosynthetic gene cluster of this strain. Thus, we failed to assign the D or L configurations for all the amino acids by the feeding experiments. However, we succeeded in proposing the complete stereochemistry of the molecule by careful analysis of the biosynthetic gene cluster (nonribosomal peptide synthetases, NRPS). These results will be reported in due course. The present studies gave some information of which structural units are required for the toxic activity, but are not sufficient. Further investigations are necessary to address the structure–activity relationship.

## Experimental

### General analytical methods

NMR spectra were recorded in acetone-*d*_6_ on a Bruker DMX 600 spectrometer; the chemical shifts (δ) are given in ppm relative to the residual solvent peaks (δ_H_ 2.04 and δ_C_ 29.80) as the internal references for the ^1^H and ^13^C NMR spectra, respectively. In the case of DMSO-*d*_6_ solutions, the chemical shifts are given in ppm relative to the solvent peaks (δ_H_ 2.49 and δ_C_ 39.50). The coupling constants, *J*, are given in Hz. FABMS spectra were obtained on a JEOL SX 100 spectrometer using *m*-nitrobenzyl alcohol (NBA). A Shimadzu LC-10A HPLC apparatus and a Shimadzu SPD-10AVP detector were used to determine the D or L configuration of each amino acid.

#### Bioassay method

The necrotic lesions were detected by the method given in Figure S19A (Supporting Information File 1). The lesions were usually detected after standing for 1 day. In the cases of the mono- and tetraacetates and compounds **A** and **B**, the necrotic activities significantly decreased; thus, the samples were allowed to stand for 3 days to estimate the activities, as shown in Supporting Information File 1, Figure S19B.

#### Isolation of cichorinotoxin

*Pseudomonas cichorii* isolate YM8705 has been deposited in NARO Genebank as MAFF730229 (NARO: National Agricultural and Food Research Organization).

*P. cichorii* YM8705 was cultured in YPD medium (polypeptone (10 g, Nippon Shinyaku Ltd), dry yeast extract (5 g, Oxoid Ltd.), NaCl (3 g) and mannitol (5 g, Wako Ltd.) in 1000 mL of H_2_O). Precultured seeds were grown at 28 °C for 24 h in 100 mL of YPD medium using a 500 mL Sakaguchi flask with reciprocal shaking. Next, 10 mL of the precultured seed culture was inoculated into 1000 mL of the YPD medium and cultured at 28 °C for 72 h. The cells obtained from 6 L of culture were collected by centrifugation and lyophilized. The dried cells were soaked in approximately 1.0 L of MeOH and stirred at room temperature for 2 h. The cells were then removed by filtration. The methanolic fraction, which contained the crude cichorinotoxin, was developed with a mixed solvent of (CH_3_)_2_CO/MeOH (2:1) on a SiO_2_ TLC. The toxin was visualized as a yellowish white spot by spraying either a BTB reagent or deionized water. Crude cichorinotoxin was obtained by SiO_2_ column chromatography eluting with the above solvent. Pure cichorinotoxin was obtained by reversed-phase HPLC (column: Shiseido CAPCEL PAK C_18_ UG120; mobile phase: MeOH/1% aqueous NH_3_ = 87:13, detected at 254 nm) in a yield of approximately 122 mg. Melting point: 181–184 °C.

#### Preparation of the monoacetate and the tetraacetate

This toxin (6.0 mg) was dissolved in 5 mL of a mixture (1:1) of CH_3_CN and 0.01 M carbonate buffer solution (pH 9.7), and then 1.5 mL of Ac_2_O was added. The reaction was carried out at room temperature for 6 h. Almost all of the toxin had been consumed by SiO_2_ TLC analysis. Complete purification was achieved by reversed-phase HPLC (MeOH/1% aq NH_3_ = 83:17), yielding 5.4 mg of the monoacetate. Cichorinotoxin (8.0 mg) was dissolved in Py (3.0 mg), and 1.5 mL of Ac_2_O was added. The reaction was continued for 1 h at ambient temperature, and the crude material was purified by the same HPLC conditions as described for the monoacetate to afford 6.8 mg of the tetraacetate.

#### Determination of the amino acid composition and of the D and L configurations

To 1.0 mg of cichorinotoxin was added 1.0 mL of 6 N HCl, and the mixture was frozen at −30 °C and degassed under a high vacuum pump. The glass vessel was sealed and heated at 110 °C for 5 h, 10 h and 22 h to obtain the hydrolysates, each of which were concentrated to dryness. The residues were evaluated using an amino acid analyzer. Marfey’s method was employed to determine the stereochemistry of each of the amino acids. The dried hydrolysates obtained by heating for 10 h were dissolved in 140 μL of H_2_O. To 25 μL of the solution were added 1% Marfey’s reagent ((1-fluoro-2,4-dinitro-5-fluorophenyl)-L-alaninamide, 1.8 μM) dissolved in acetone and 10 μL of 1 M NaHCO_3_ (10 μM). The solution was heated at 35 °C for 1 h, then 2 N aq HCl was added to quench the reaction, and then it was concentrated to dryness. The residues were dissolved in DMSO and subjected to reversed-phase HPLC (C_18_) using a mobile phase composed of 13% CH_3_CN/87% 50 mM triethylamine phosphate. Amino acid compositions of natural cichorinotoxin (not Marfey’s derivatives) were analyzed by Hitachi Keisoku Service Ltd (Tokyo).

#### Partially hydrolyzed peptide fragments

Seven milliliters of 12 N HCl was added to 15 mg of cichorinotoxin, and the reaction was allowed to stand at room temperature for 20 h. The hydrolysates were subjected to reversed-phase HPLC (the same ODS column) using MeOH/H_2_O (58:42) with 0.3% NH_3_ and afforded 6 major peaks. After purification, only one peak remained in the HPLC chromatogram, and this compound was analyzed by FABMS. To clarify the stereochemistry of the amino acids present, Marfey’s method was employed.

#### Hydrolysis of cichorinotoxin in a basic medium

The purified toxin (26 mg) was dissolved in 0.02 N KOH/MeOH and allowed to stand at ambient temperature for 5 h. This reaction mixture was neutralized with 2 N aq HCl and then lyophilized. The residue was subjected to reversed-phase HPLC (C18) eluting with MeOH/1% aq NH_3_ (71:29), and the eluate was monitored at 254 nm. New peaks from compounds **A** (earlier elution) and **B** (later elution) were observed in addition to the peak of cichorinotoxin. The isolated yields were as follows: 2.5 mg for compound **A**; 6.3 mg for compound **B**; and 6.1 mg of recovered cichorinotoxin.

#### Feeding experiments of D- and L-[1-^13^C]Val and -Ala

*P. cichorii* YM8705 was grown in the culture medium composed of the following compositions: mannitol (10 g), Na_2_HPO_4_·12H_2_O (3 g), KH_2_PO_4_ (0.5 g), NaCl (3 g), (NH_4_)_2_SO_4_ (1 g), MgSO_4_·7H_2_O (25 mg), CaCl_2_·2H_2_O (23 mg) and dry yeast extract (0.1 g) dissolved in 1000 mL of H_2_O. To the 500 mL Sakaguchi flask containing 100 mL medium, 25–40 mg of the labelled Val and Ala was aseptically added and then grown at 28 °C for 120 h. The labelled cichorinotoxins were isolated by the same HPLC conditions as described above.

## Supporting Information

File 1 Analytical data (^1^H, ^13^C NMR, IR and MS), photographs of bioassay methods.
