# Supplementary material for: Chemical structure of cichorinotoxin, a cyclic lipodepsipeptide that is produced by Pseudomonas cichorii and causes varnish spots on lettuce
Source: Beilstein J Org Chem. 2019 Feb 1;15:299–309. doi: 10.3762/bjoc.15.27 (PMC6369977; doi:10.3762/bjoc.15.27)
Supplement: File 1 — Analytical data (1H, 13C NMR, IR and MS), photographs of bioassay methods. [file Beilstein_J_Org_Chem-15-299-s001.pdf]

BEILSTEIN JOURNAL OF ORGANIC CHEMISTRY

Supporting Information

for

Chemical structure of cichorinotoxin, a cyclic lipodepsipeptide that is produced by *Pseudomonas cichorii* and causes varnish spots on lettuce

Hidekazu Komatsu, Takashi Shirakawa, Takeo Uchiyama and Tsutomu Hoshino

Beilstein J. Org. Chem. **2019,** *15,* 299–309. doi:10.3762/bjoc.15.27

Analytical data (^{1}H, ^{13}C NMR, IR and MS), photographs of bioassay methods

License and Terms: This is a supporting information file under the terms of the Creative Commons Attribution License (http://creativecommons.org/licenses/by/4.0). Please note that the reuse, redistribution and reproduction in particular requires that the authors and source are credited.
The license is subject to the *Beilstein Journal of Organic Chemistry* terms and conditions: (https://www.beilstein-journals.org/bjoc)

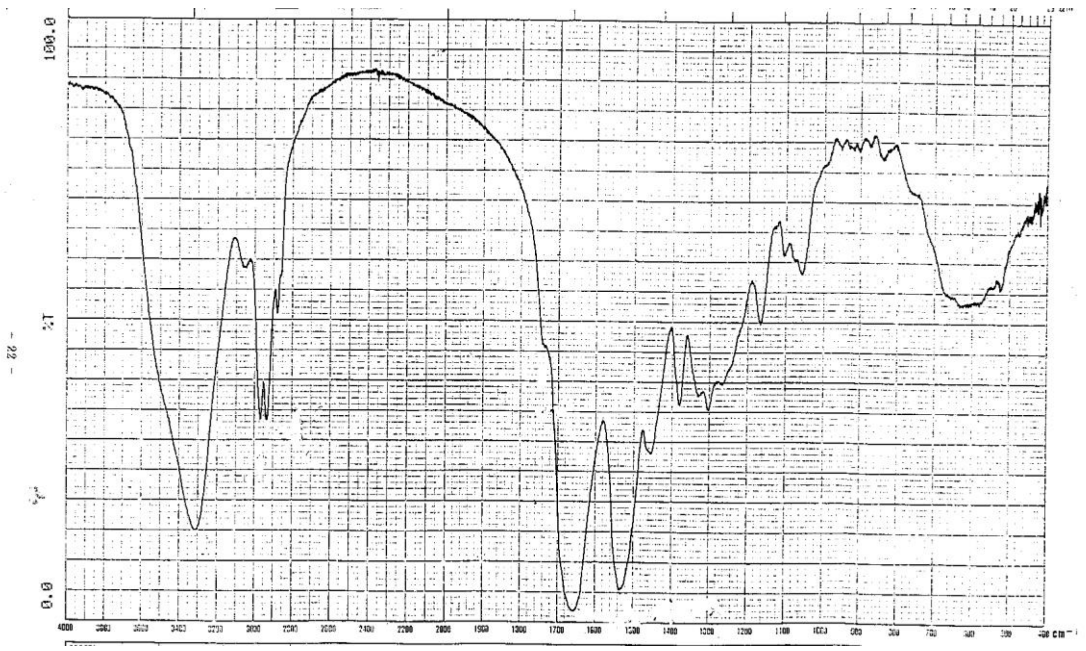

Figure S1: IR spectrum of cichorinotoxin (KBr method).

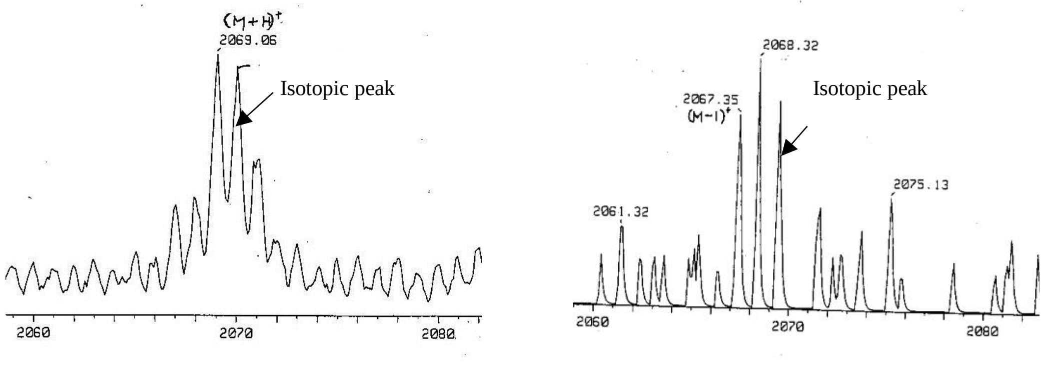

Figure S2: FABMS spectra of cichorinotoxin (matrix, m-NBA: *m*-nitrobenzenzyl alcohol); left, positive mode; right, negative mode.

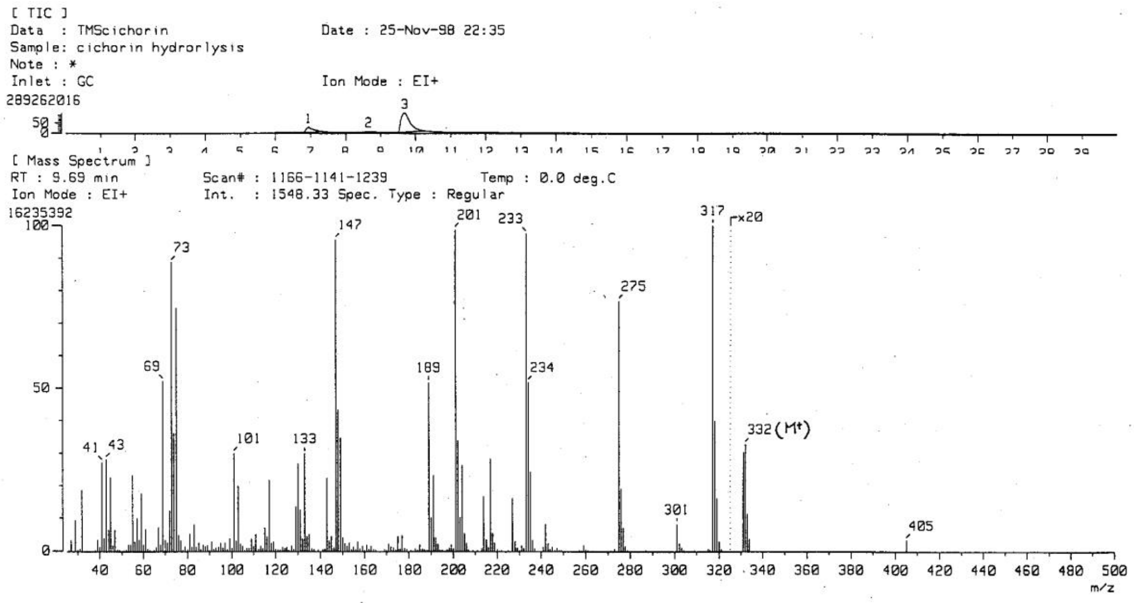

Figure S3A: GC–EIMS of the TMS derivative of the fatty acid included in cichorinotoxin.

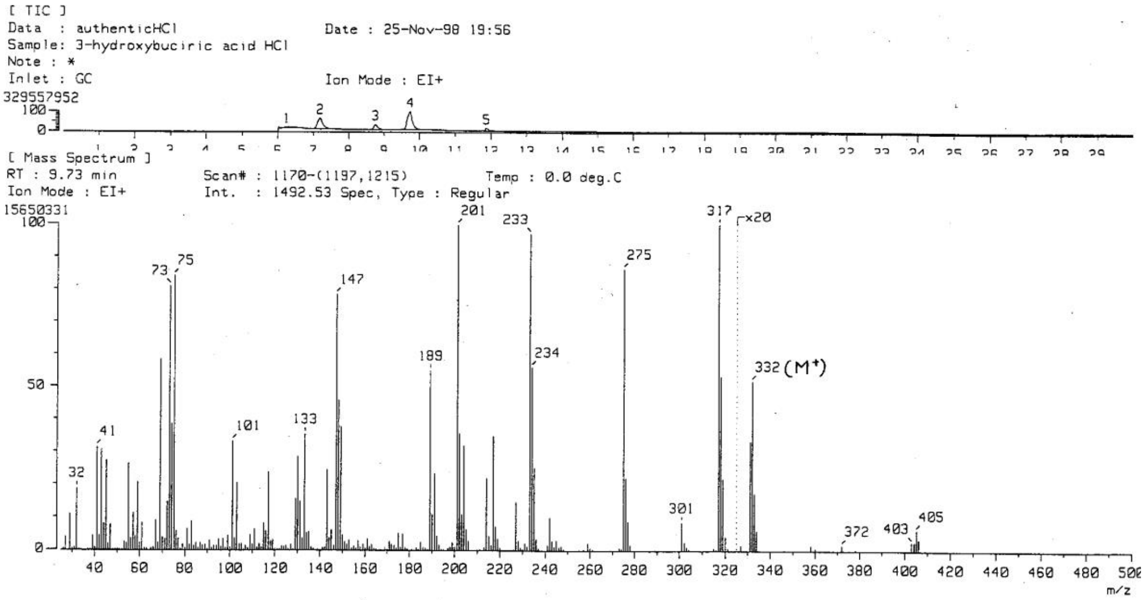

Figure S3B: GC–EIMS of the TMS derivative of commercially available 3-hydroxydecanoic acid.

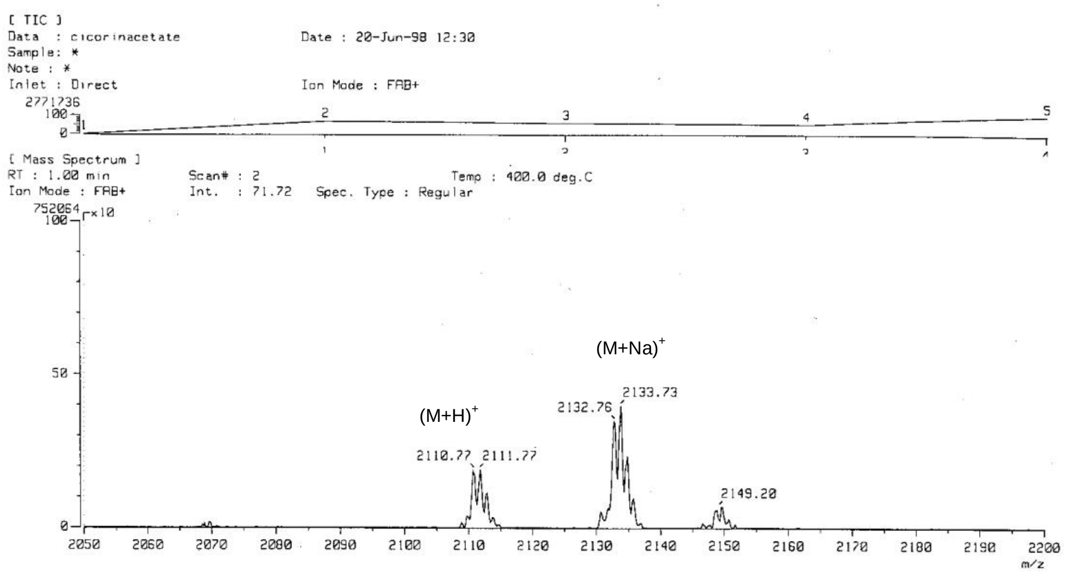

Figure S4A: FABMS spectrum of monoacetate of chicorinotoxin (+, NBA matrix), prepared with Ac_2O dissolved in a mixture of CH_3CN/pH 9.7 aq buffer solution.

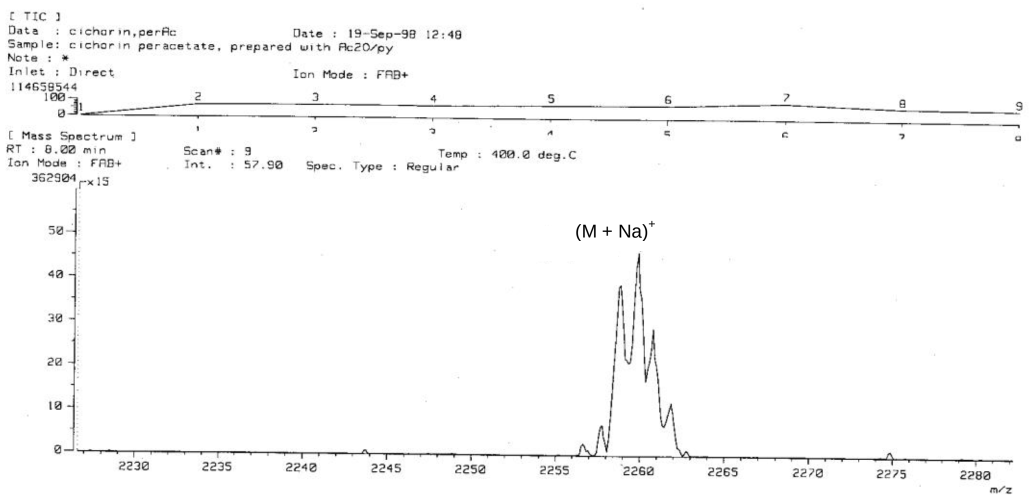

Figure S4B: FABMS spectrum of tetraacetate (peracetate) of chicorinotoxin (+, NBA matrix), prepared with Ac_2O/Py.

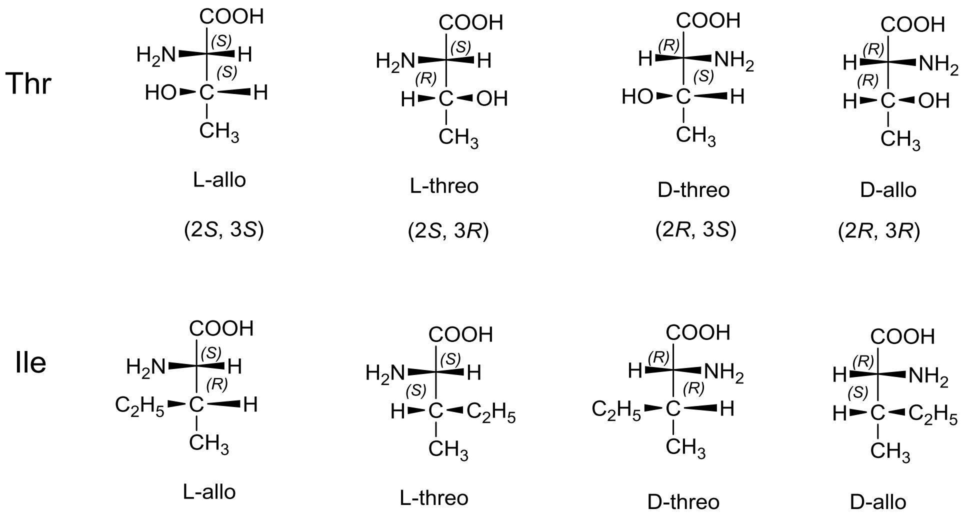

Figure S5A: Four different diastereomers of Thr and Ile amino acids.

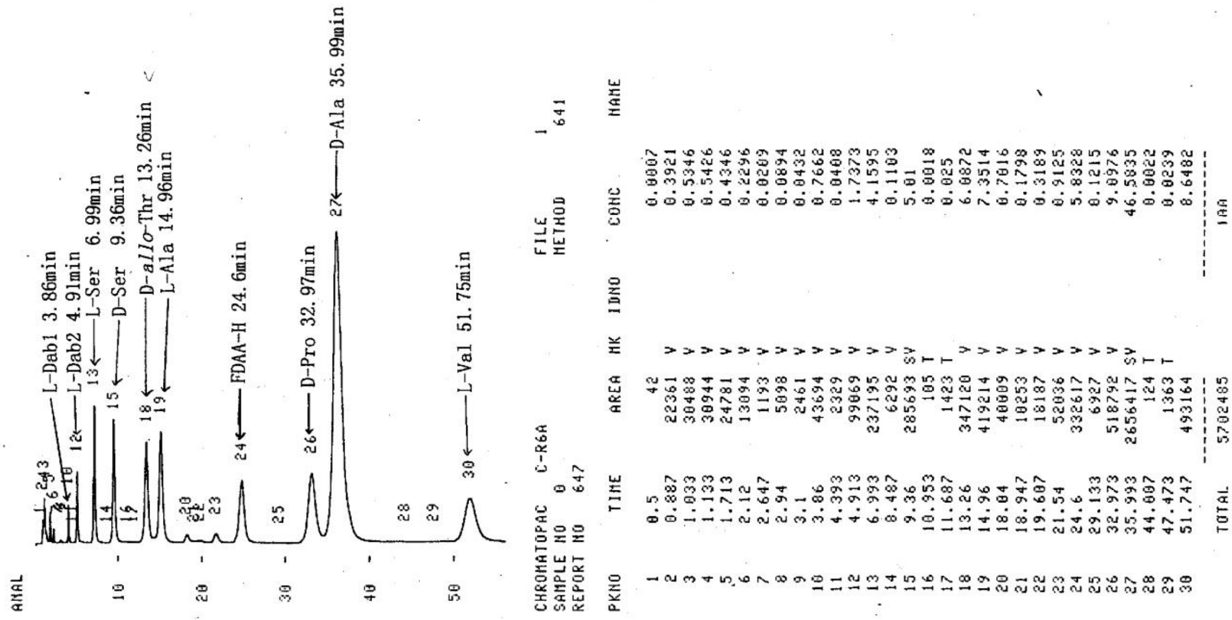

Figure S5B: HPLC profile of Marfey's derivatives of amino acids obtained from cichorinotoxin (elution solvent: 13% CH_3CN–87% 50 mM trimethylamine phosphate buffer (pH 3.0)).

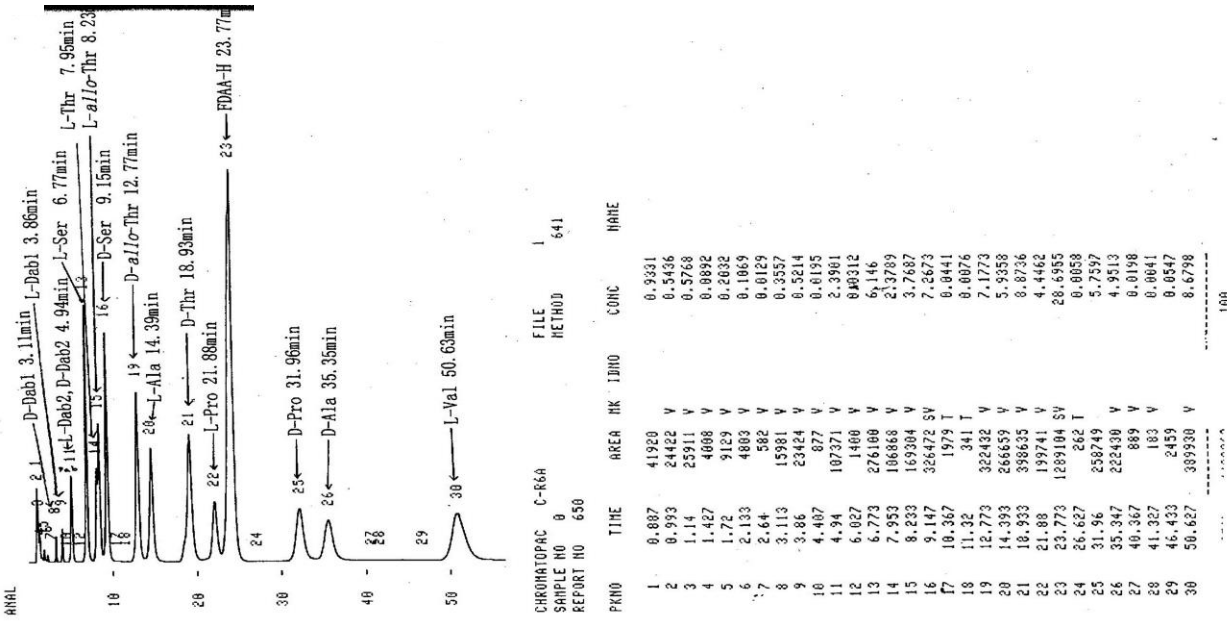

Figure S5C: HPLC profile of Marfey's derivatives of authentic amino acids (elution solvent: 13% CH_3CN-87% 50 mM trimethylamine phosphate buffer (pH 3.0)). HPLC conditions were the same as those of Fig. S5B. The derivatives of authentic amino acids to be injected were as follows: D, L-Ser; D,L-Thr; D, L-*allo*Thr; D, L-Ala, D, L-Pro, L-Val, the D and L-isomers of these amino acids having been separated. HPLC conditions: column: Shiseido CAPCELL PACK C18, UG120, S-3mm (1.6 × 100 mm), HPLC apparatus: Shimadzu LC-10A, flow rate 1.5 ml/min, detected at 340 nm.

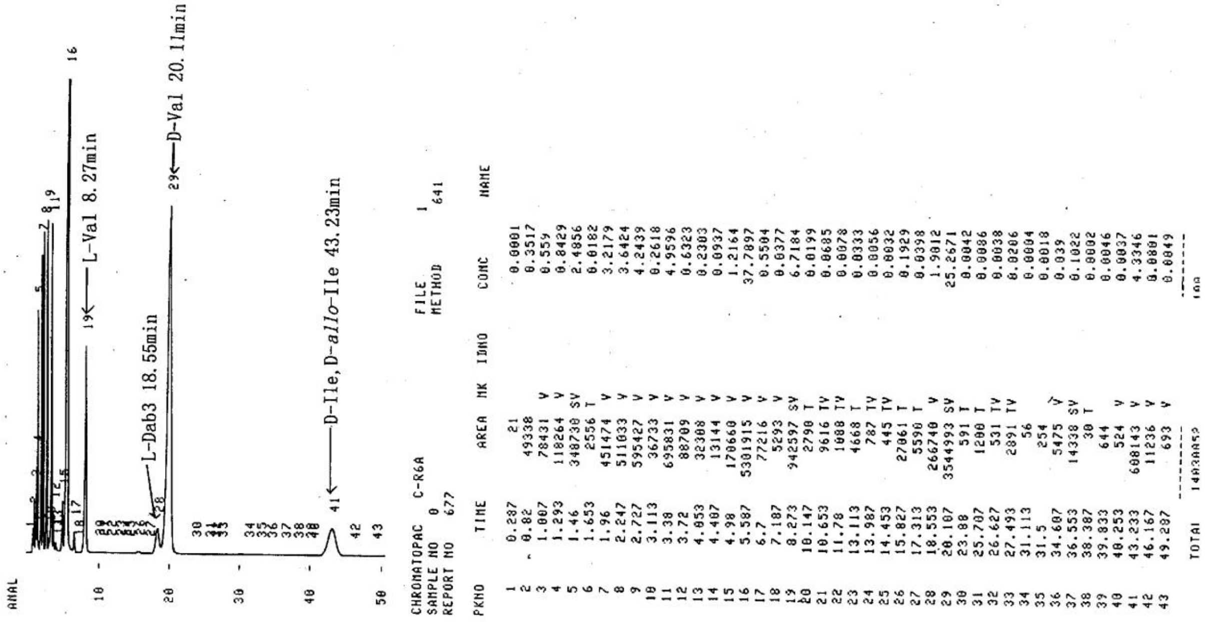

Figure S5D. HPLC profile of Marfey's derivatives of amino acids obtained from cichorinotoxin (elution solvent: 25%CH_3CN–75% 50 mM trimethylamine phosphate buffer (pH 3.0)). HPLC conditions were the same as those of Figure S5B and 5C except elution solvent.

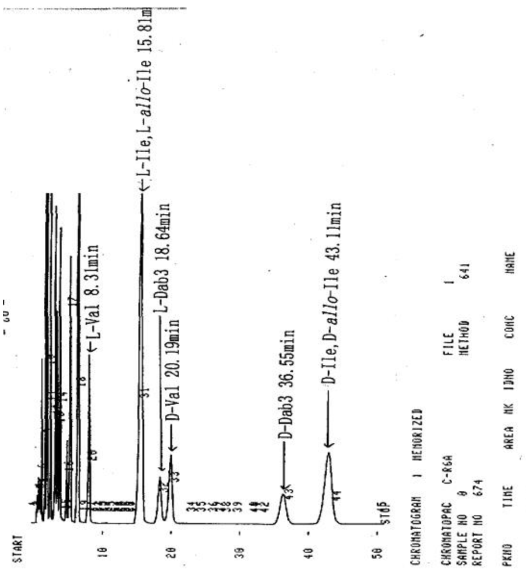

Figure S5E: HPLC profile of Marfey's derivatives of authentic amino acids. (elution solvent: 25% CH_3CN–75% 50 mM trimethylamine phosphate buffer (pH 3.0)). Injected samples were as follows: D- and L-Val; D- and L-Ile; D- and L- *allo*Ile; D- and L-Dab, the stereoisomers having been separated. HPLC conditions were the same as those of Figure S5B and 5C except elution solvent.

Conclusions: Figure S5C (elution: 13% CH_3CN) indicates that the following enantiomers were separable: L-*allo*-Thr and D- *allo*-Thr, L-Ser and D-Ser, L-Ala and D-Ala, L-Pro and D-Pro, and L-Dab and D-Dab. This information enabled us to determine from Fig. S5B that the following enantiomers are included in cichorinotoxin: D-allo-Thr, both L- and D-Ser, both D- and L-Ala, D-Pro, and L-Dab.

Figure 5E (elution: 25% CH_3CN) indicates that the separations of L-Val and D-Val and that of L-Dab3 and D-Dab3 were successful. However the separations of L-Ile and L-allo-Ile and that of D-Ile and D-allo-Ile were unsuccessful, but the D-form and L-form were completely separated. Thus, it can be concluded from Figure S5D that both L- and D-Val and L-Dab3 are involved in the toxin. Ile residue in cichorinotoxin is either the D-allo- or D-threo-type and not the L-allo- or L-threo-type. As to FDAA derivative of Dab, three peaks were observe: two peaks Dab1 and Dab2 in Figure 5C, and another peak Dab3 in Figure 5E. These peaks are assigned to be the following three compounds.

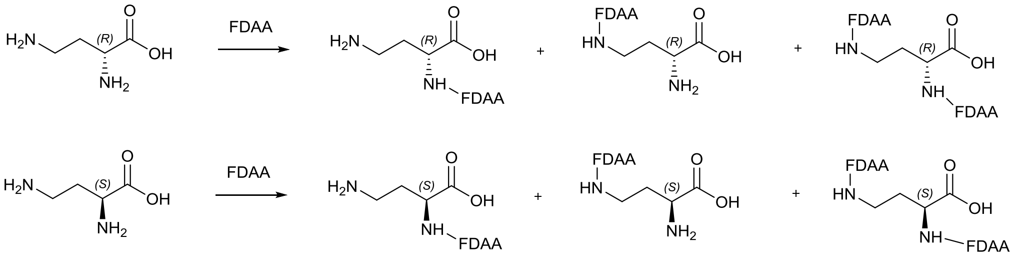

Consequently, amino acid compositions of cichorinotoxin are summarized in Table 1 (main text).

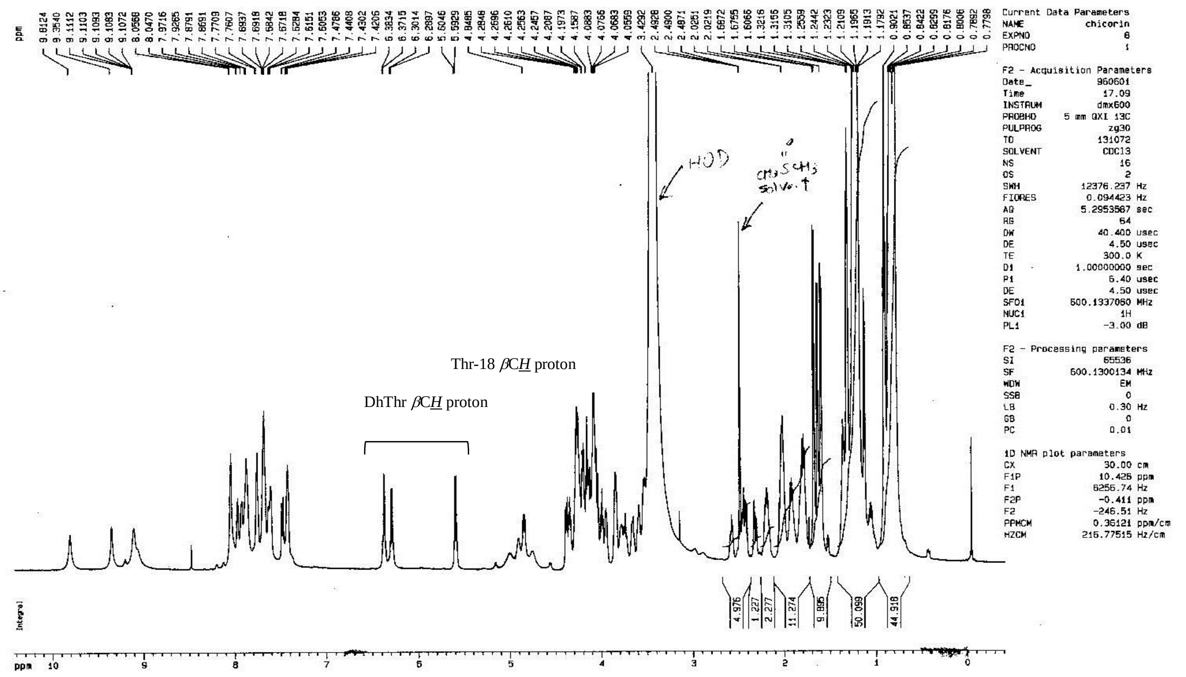

Figure S6: ^{1}H NMR spectrum of cichorinotoxin (overall region) in DMSO-d_6 measured at 600 MHz at ambient temperature. A large amount of natural cichorinotoxin is readily soluble in DMSO.

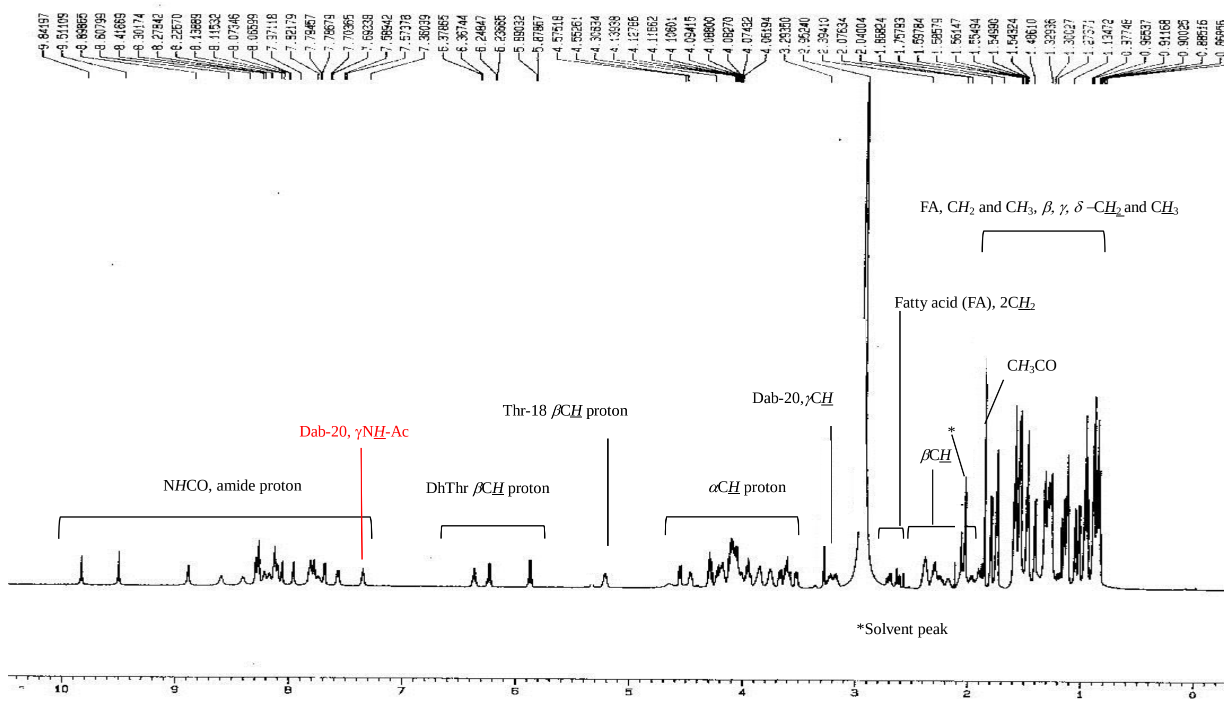

Figure S7: ^{1}H NMR spectrum of cichorinotoxin monoacetate (overall region) in acetone-d_6 measured at 600 MHz at ambient temperature.

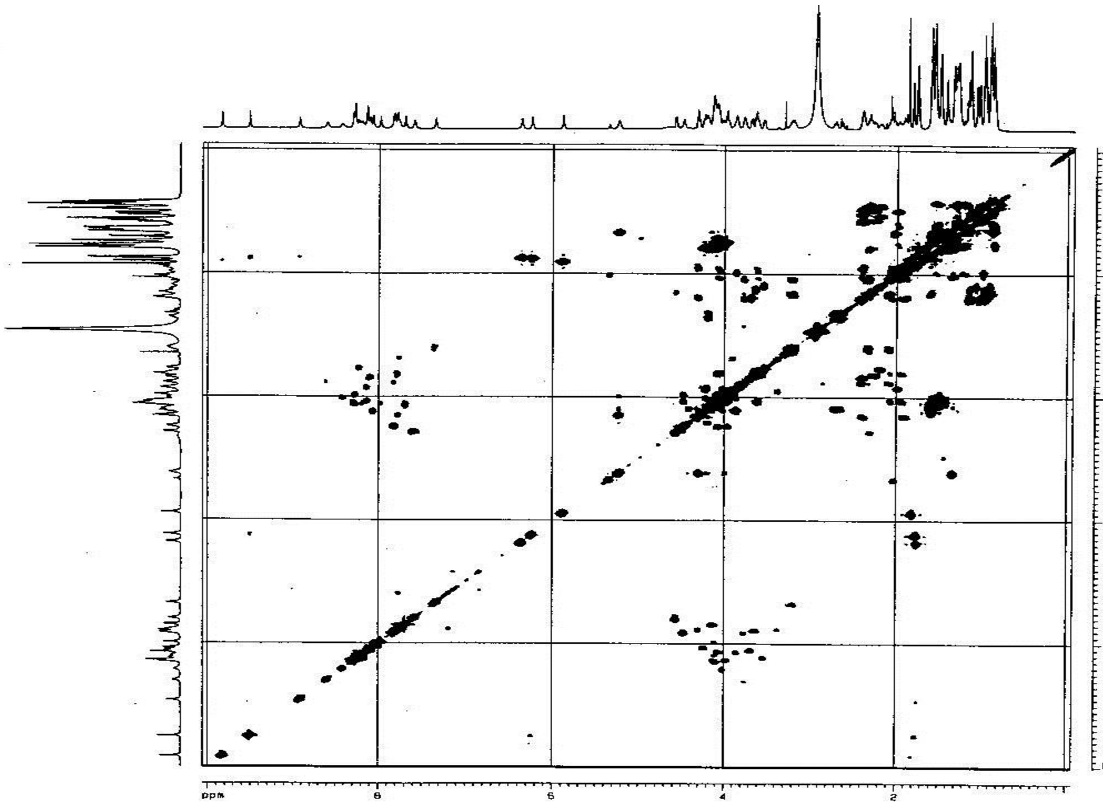

Figure 8A: $^{1}H,^{1}H$ COSY spectrum of the monoacetate (overall spectrum).

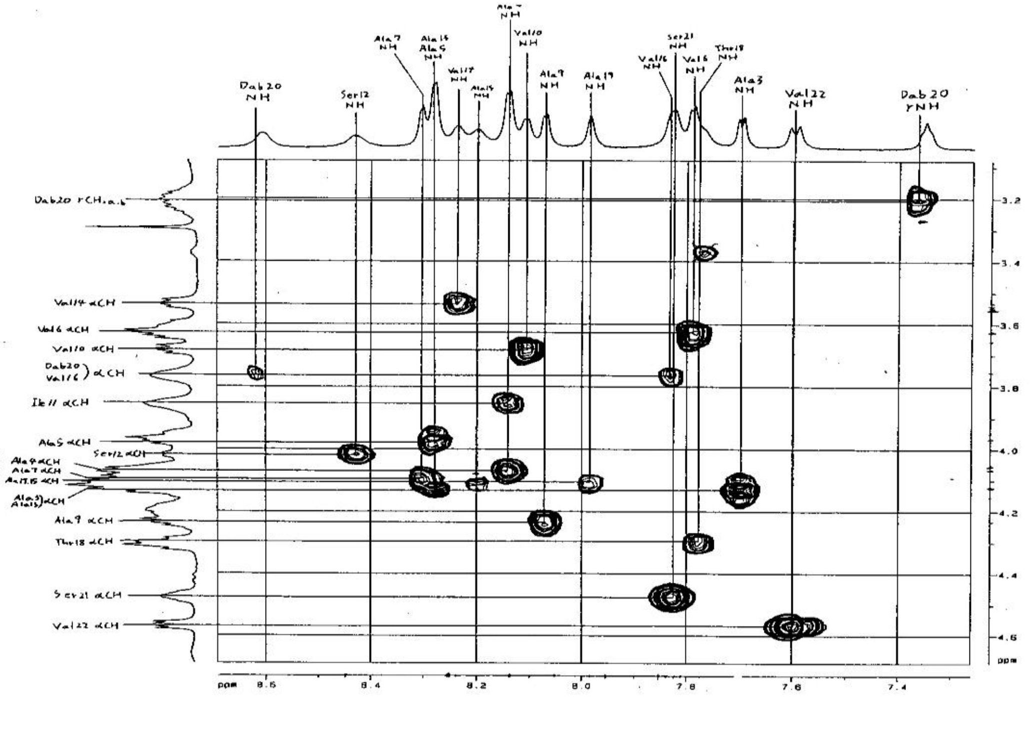

Figure S8B: $^{1}H,^{1}H$ COSY spectrum of the monoacetate, in which the spin couplings for αCH/amide NH protons are highlighted.

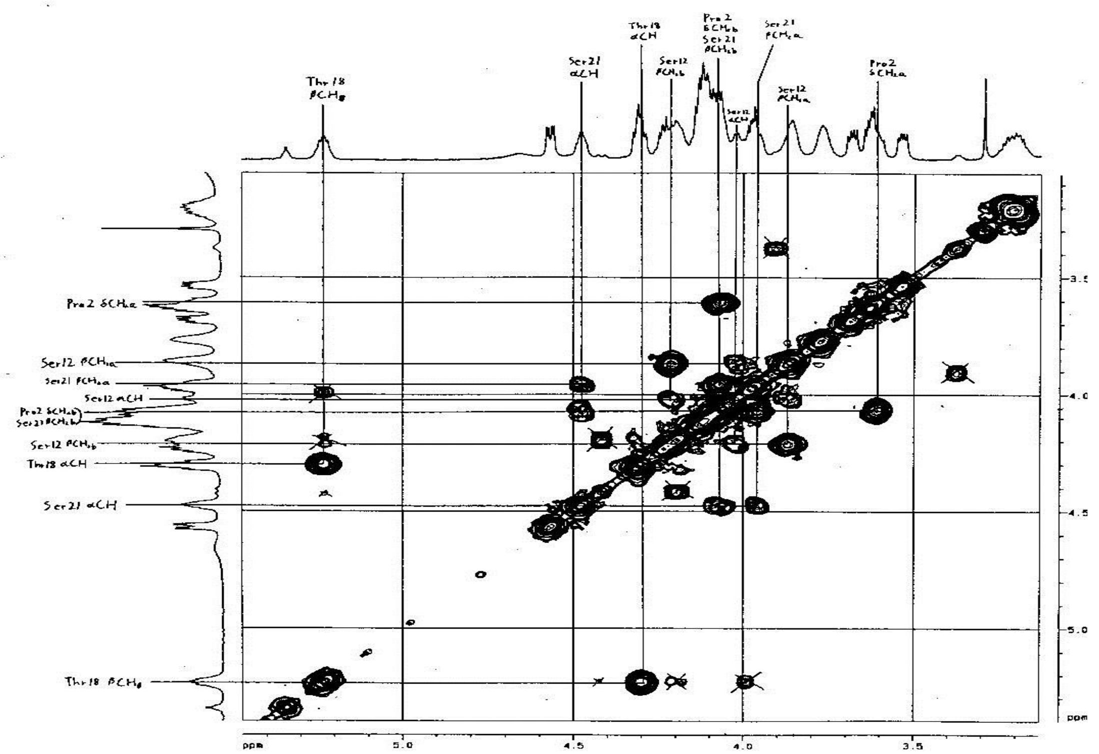

Figure S8C: $^{1}H,^{1}H$ COSY spectrum of the monoacetate, in which the proton spin–spin couplings for αCH/βCH found in Ser, Thr and Pro residues are highlighted.

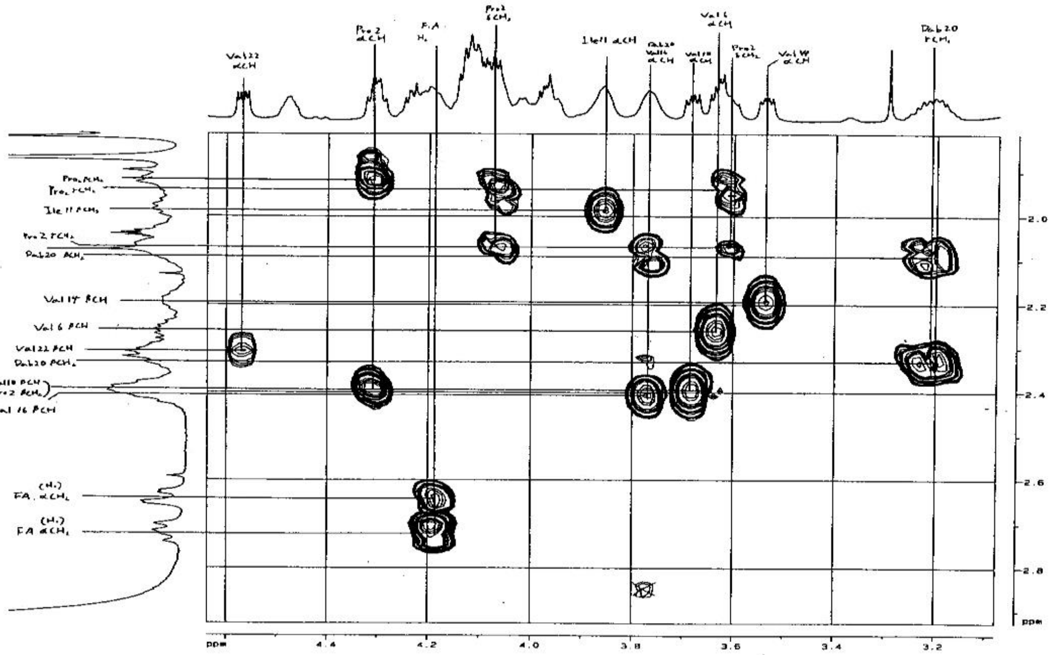

Figure S8D: $^{1}H,^{1}H$ COSY spectrum of the monoacetate, in which the COSY cross peaks of αCH/βCH found mainly in other amino acids residues are highlighted (excluding the amino acids shown in Figure S8C).

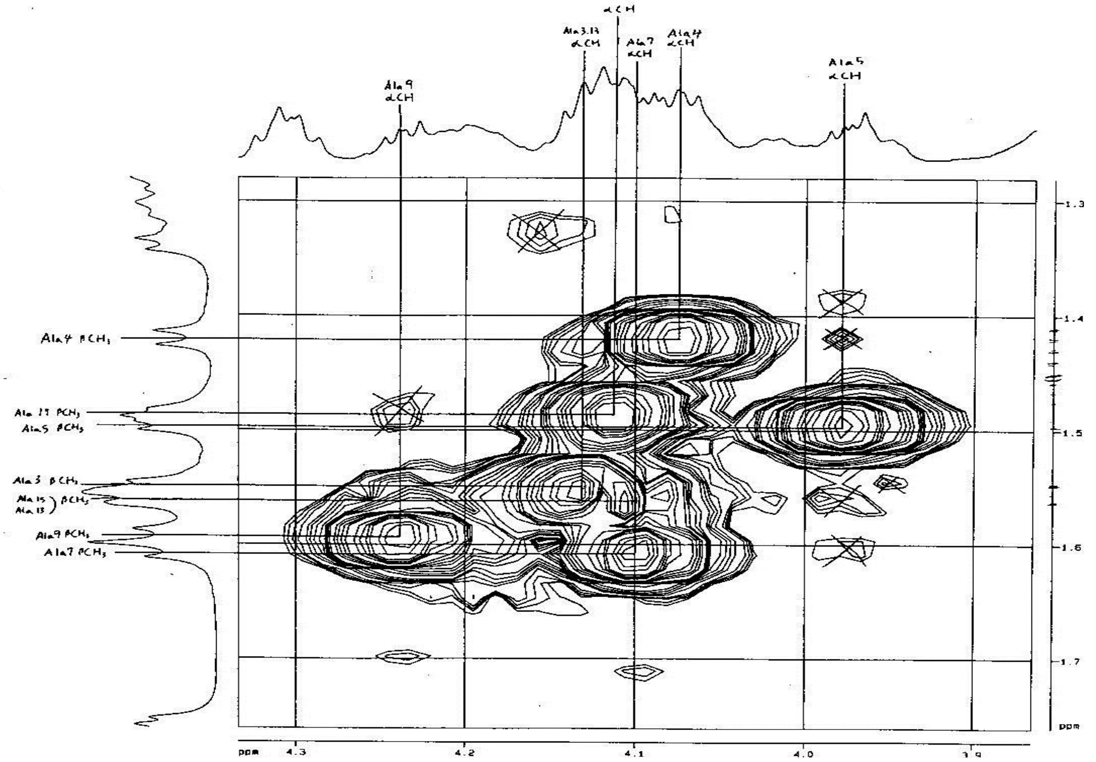

Figure S8E: ^{1}H,^{1}H COSY spectrum of the monoacetate, in which the proton spin–spin couplings for αCH/βCH found in Ala residues are highlighted.

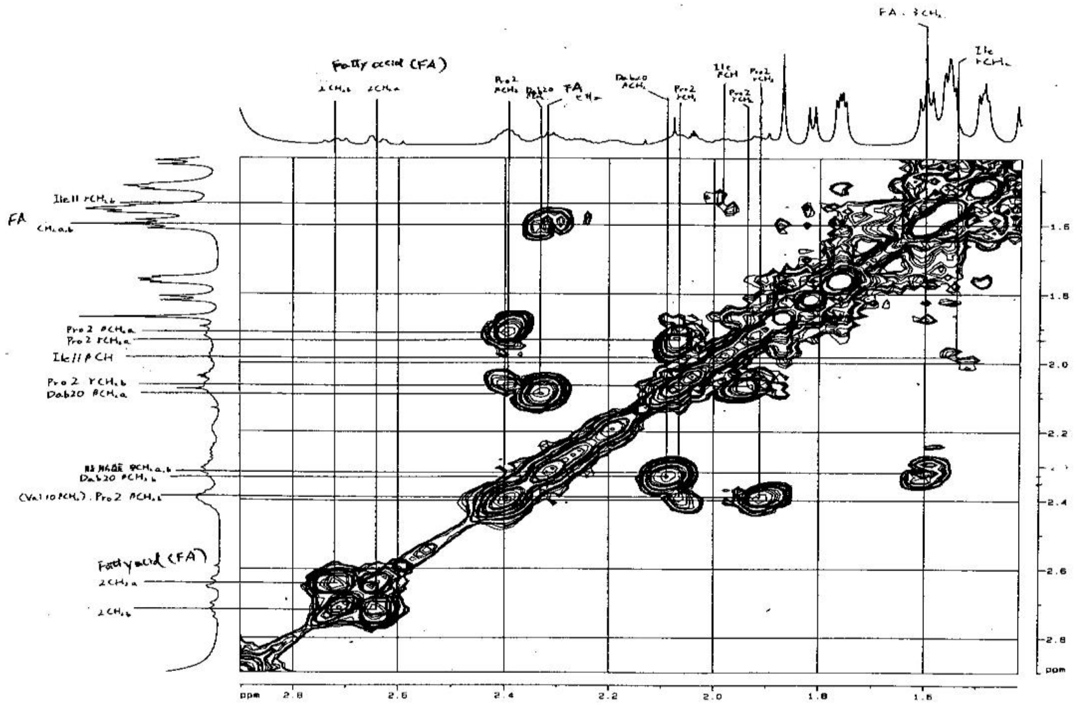

Figure S8F: ^{1}H,^{1}H COSY spectrum of the monoacetate, in which the proton spin–spin couplings for βCH/γCH of Pro, Dab and Ile residues are highlighted.

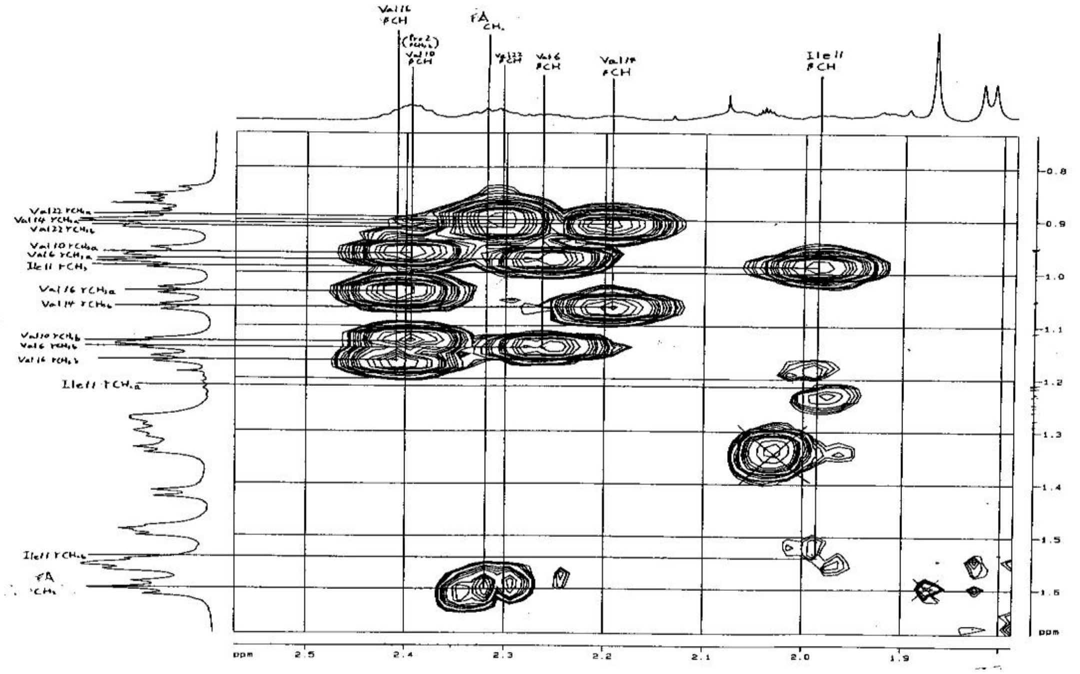

Figure S8G: $^{1}H,^{1}H$ COSY spectrum of the monoacetate, in which the proton spin–spin couplings for βCH/γCH of Val residues are highlighted.

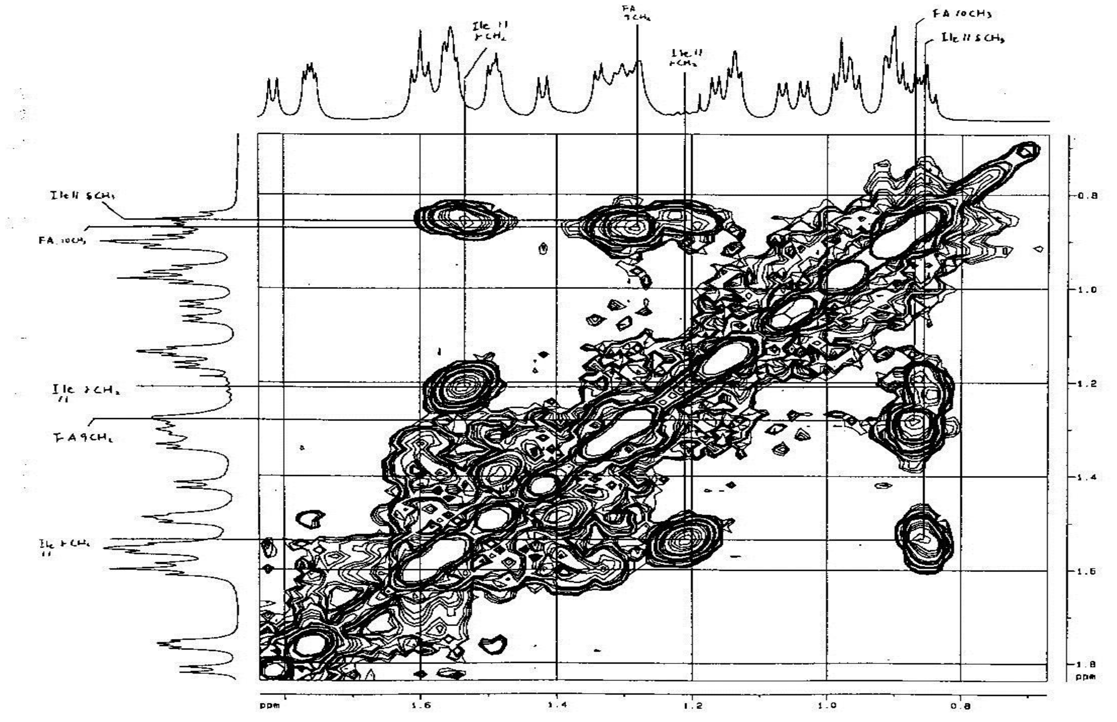

Figure S8H: $^{1}H,^{1}H$ COSY spectrum of the monoacetate, in which the proton spin–spin couplings for γCH/δCH of Ile residue are highlighted.

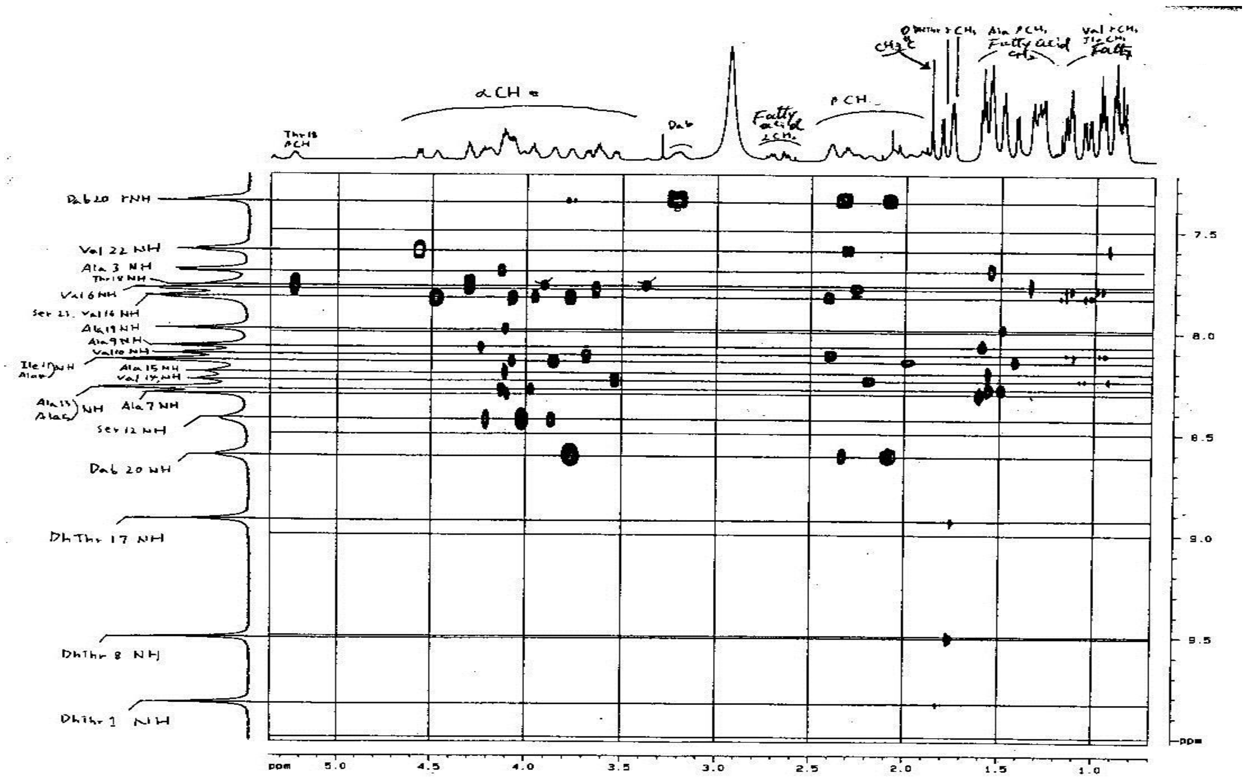

Figure S9: TOCSY spectrum of cicorinotoxin monoacetate (600 MHz, acetone d_6), in which TOCSY cross peaks from amide protons are highlighted.

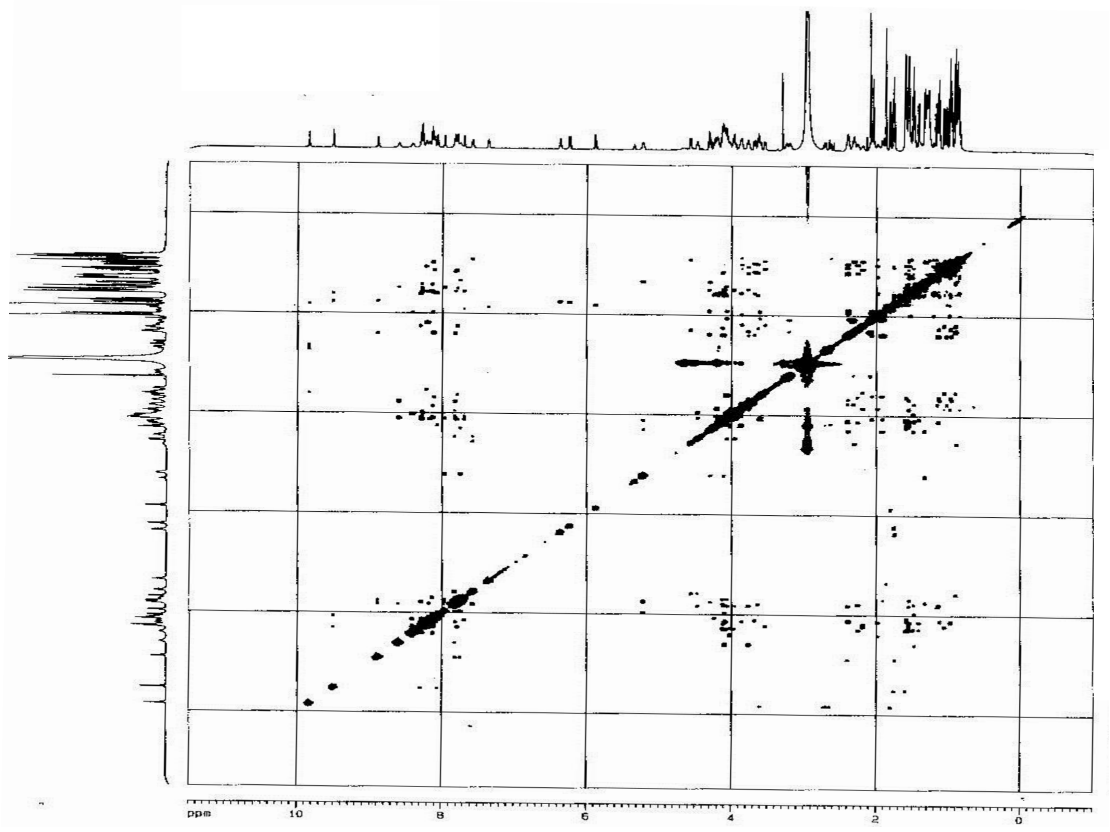

Figure S10A:. NOESY spectrum (overall region).

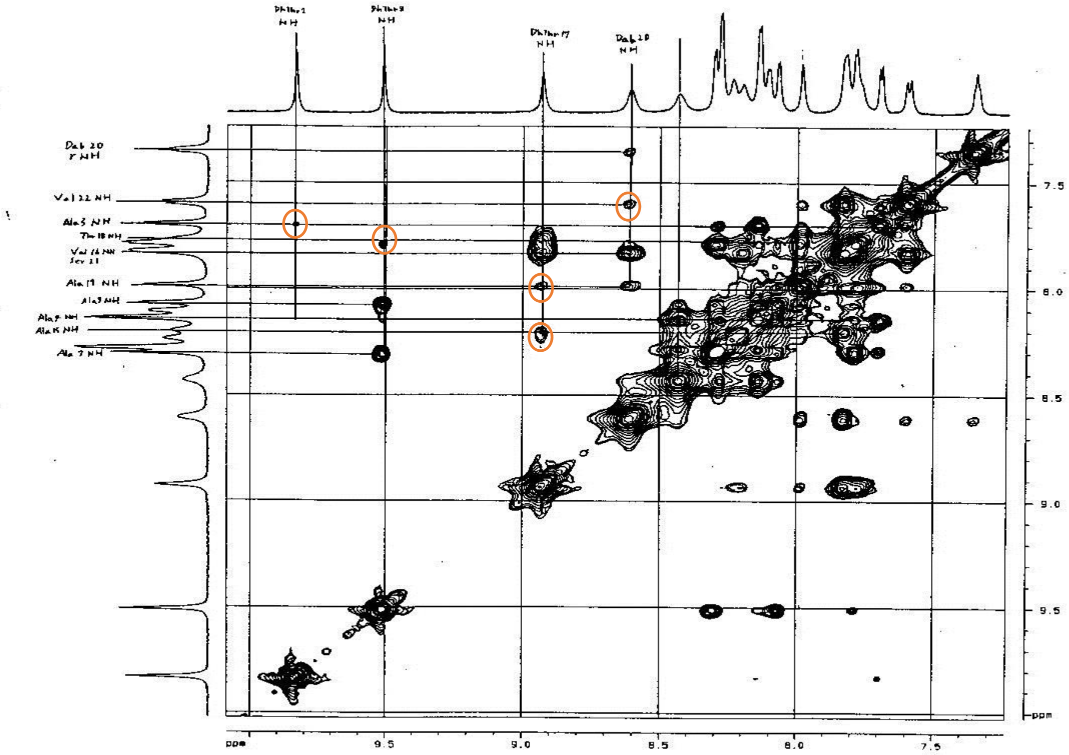

Figure S10B: NOESY correlations for $\alpha N^{i}H/\alpha N^{i+1}H$ (**Orange circles** indicate long range NOEs).

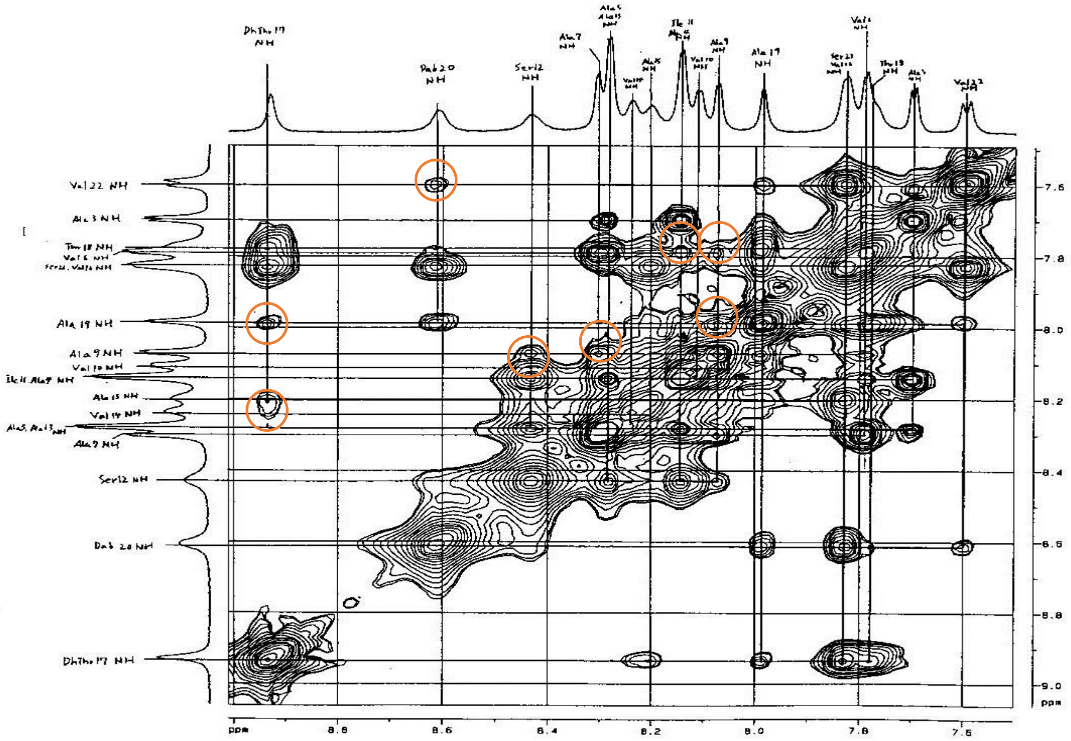

Figure S10C: NOESY spectrum of $\alpha N^{i}H/\alpha N^{i+1}H$ (expanded region); (**Orange circles** represent long range NOEs).

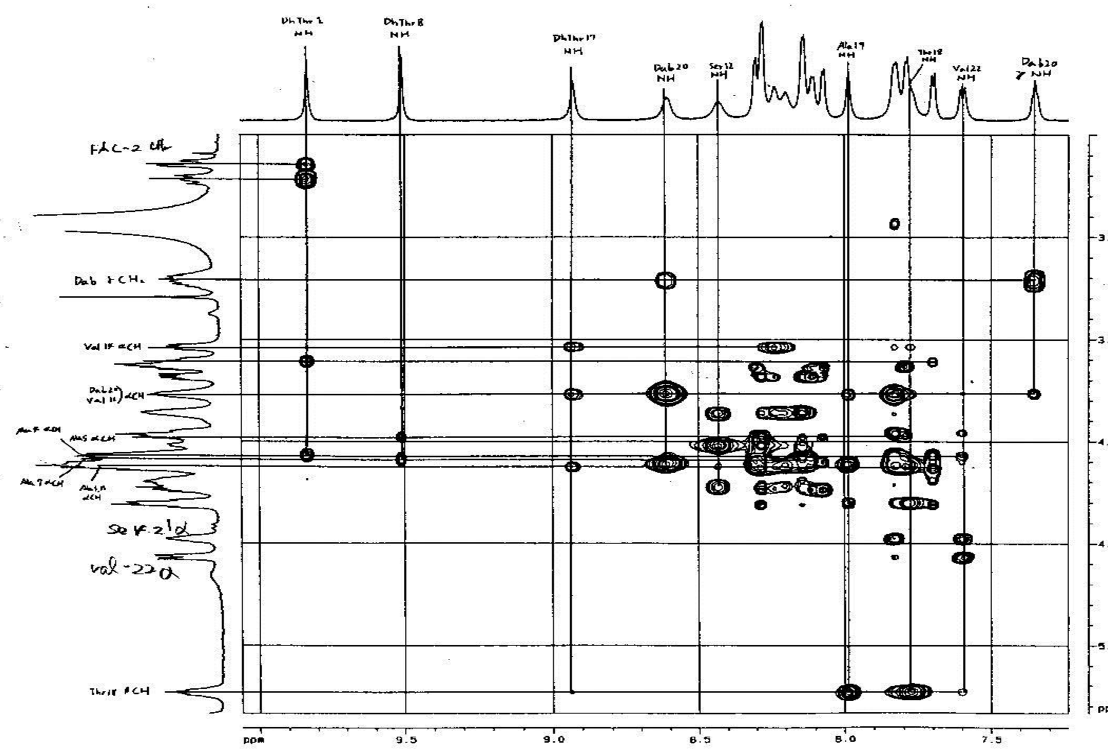

Figure S10D: NOESY spectrum representing the cross peaks of $\alpha N^{i}H/\alpha C^{i}H$, $\alpha C^{i}H/\alpha N^{i+1}H$.

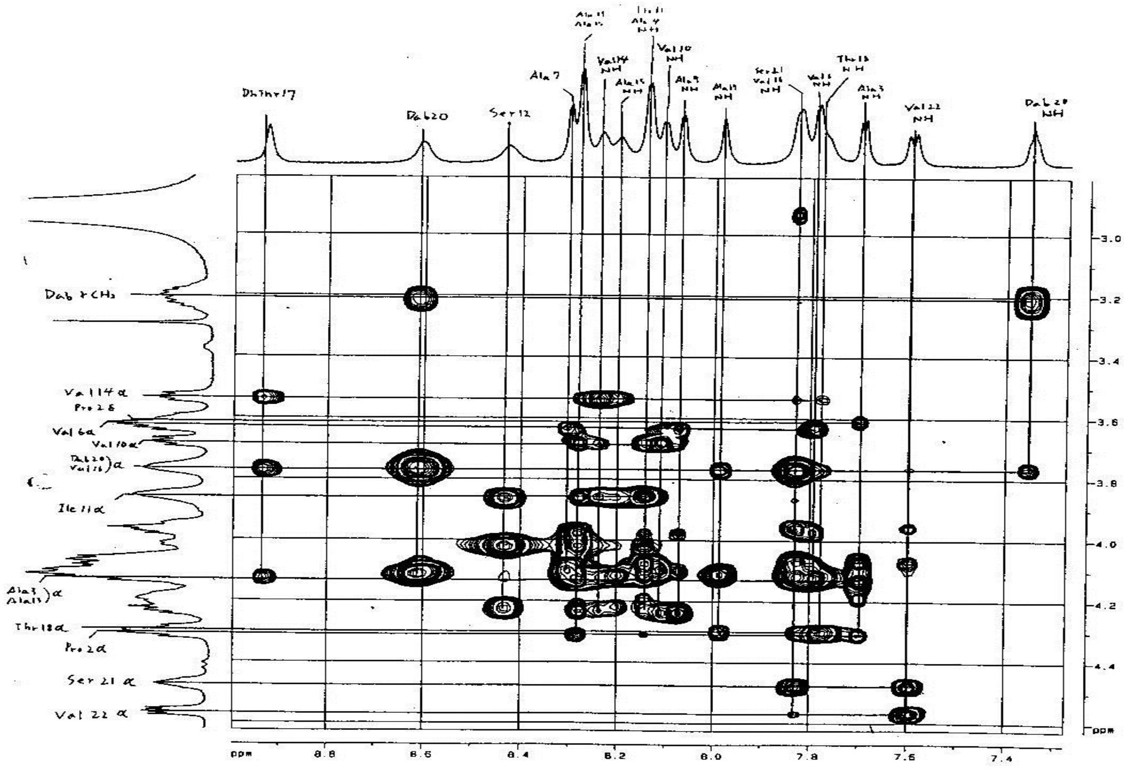

Figure S10E: NOESY spectrum representing the cross peaks of $\alpha N^{i}H/\alpha C^{i}H$, $\alpha C^{i}H/\alpha N^{i+1}H$ (expanded region of Figure S10D).

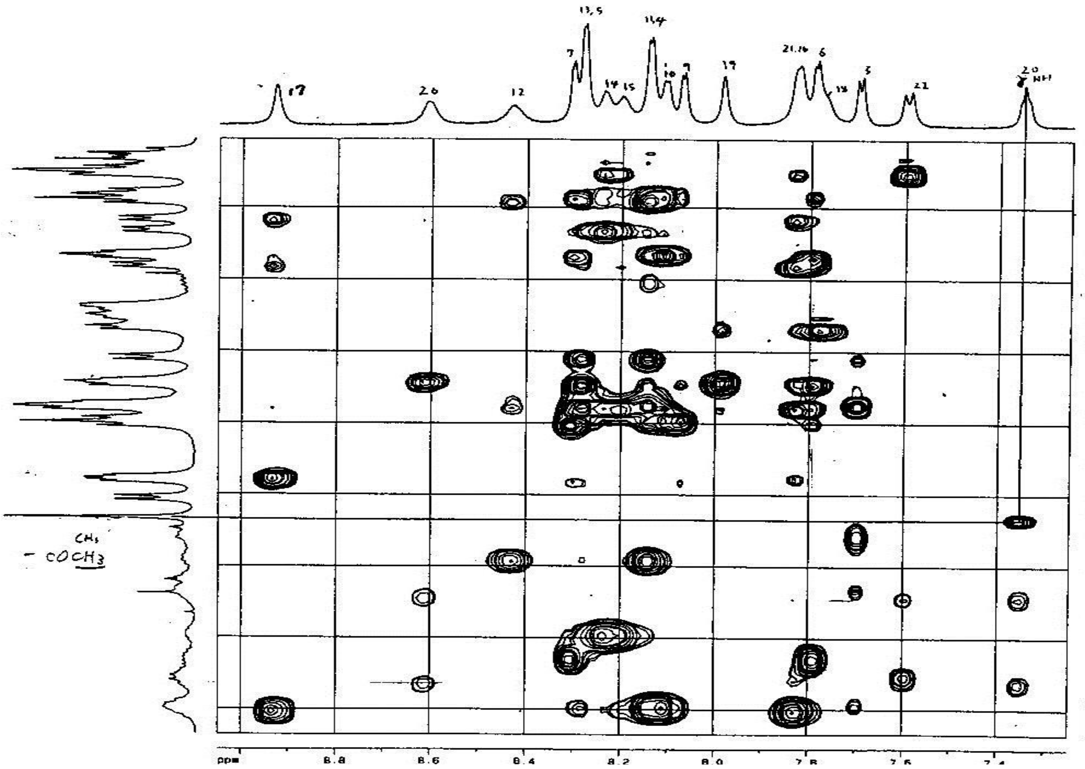

Figure S10F:. NOESY spectrum; the cross peaks of $\beta C^{i}H/\alpha N^{i+1}H$ are highlighted. The NOE between γNH of Dab and the acetyl Me of the monoacetate was clearly observed, indicating the Ac is positioned at Dab.

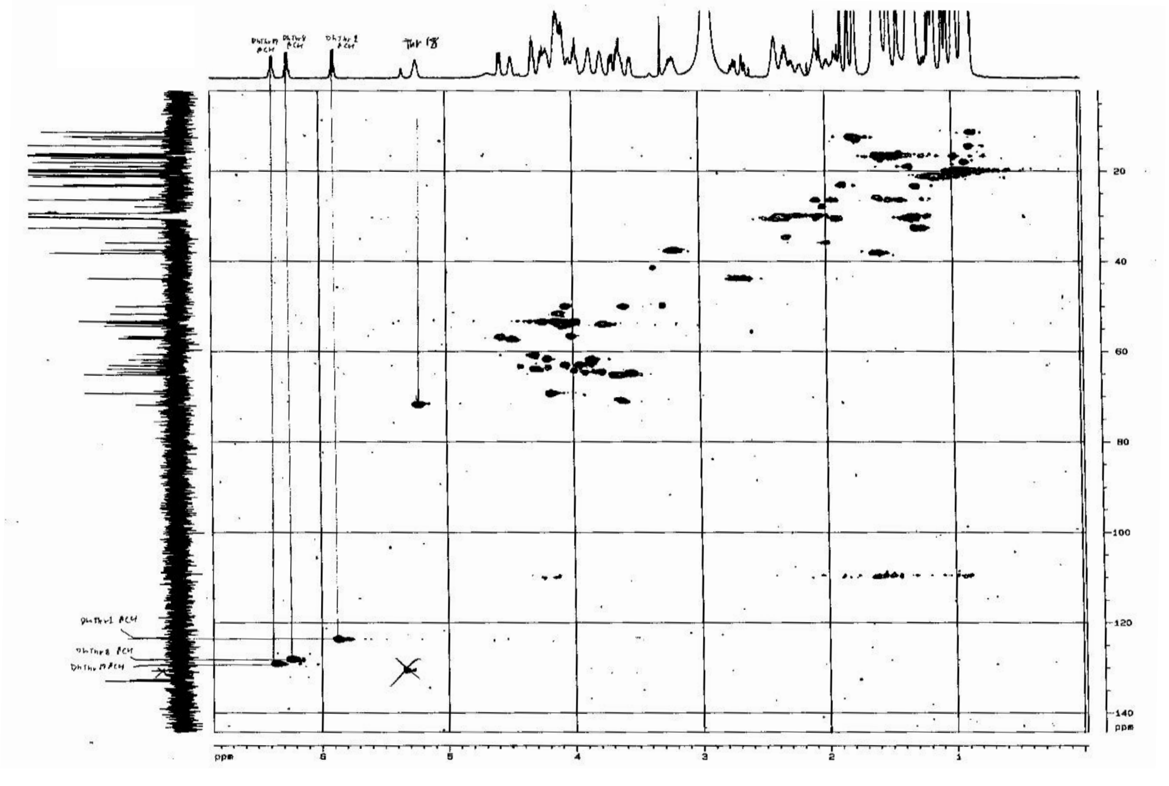

Figure S11: HMQC spectrum of the monoacetate (overall region).

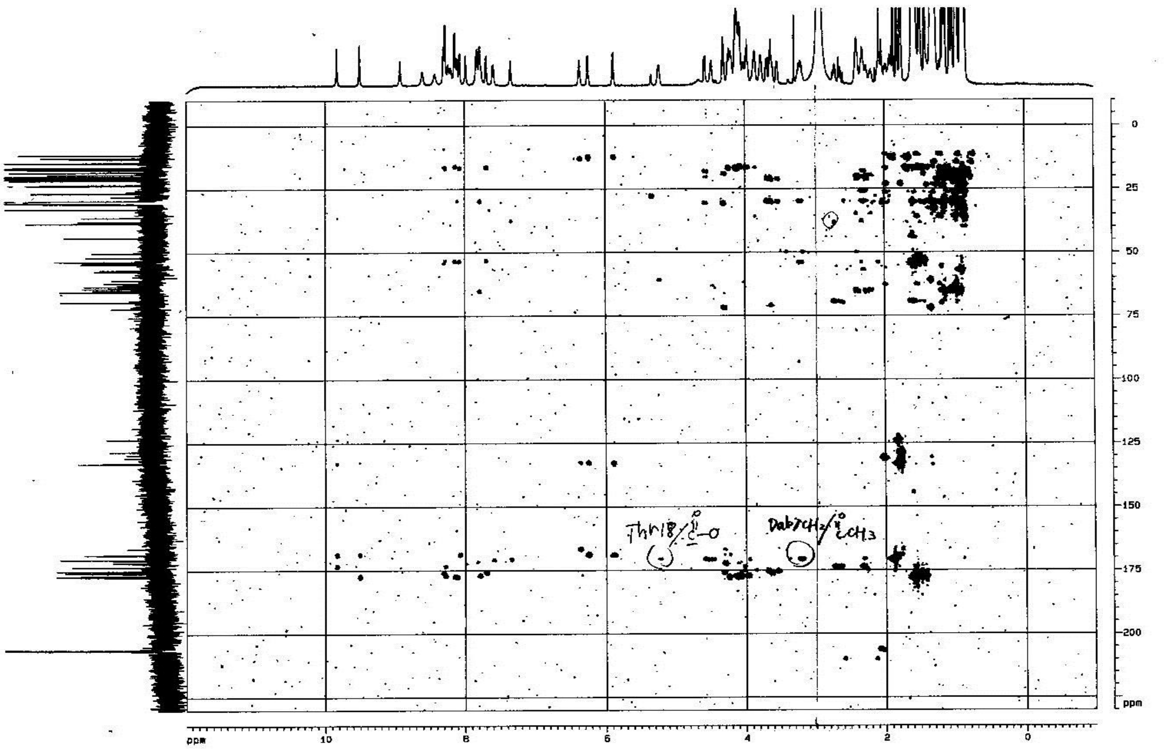

Figure S12A:. HMBC spectrum of the monoacetate (overall region).

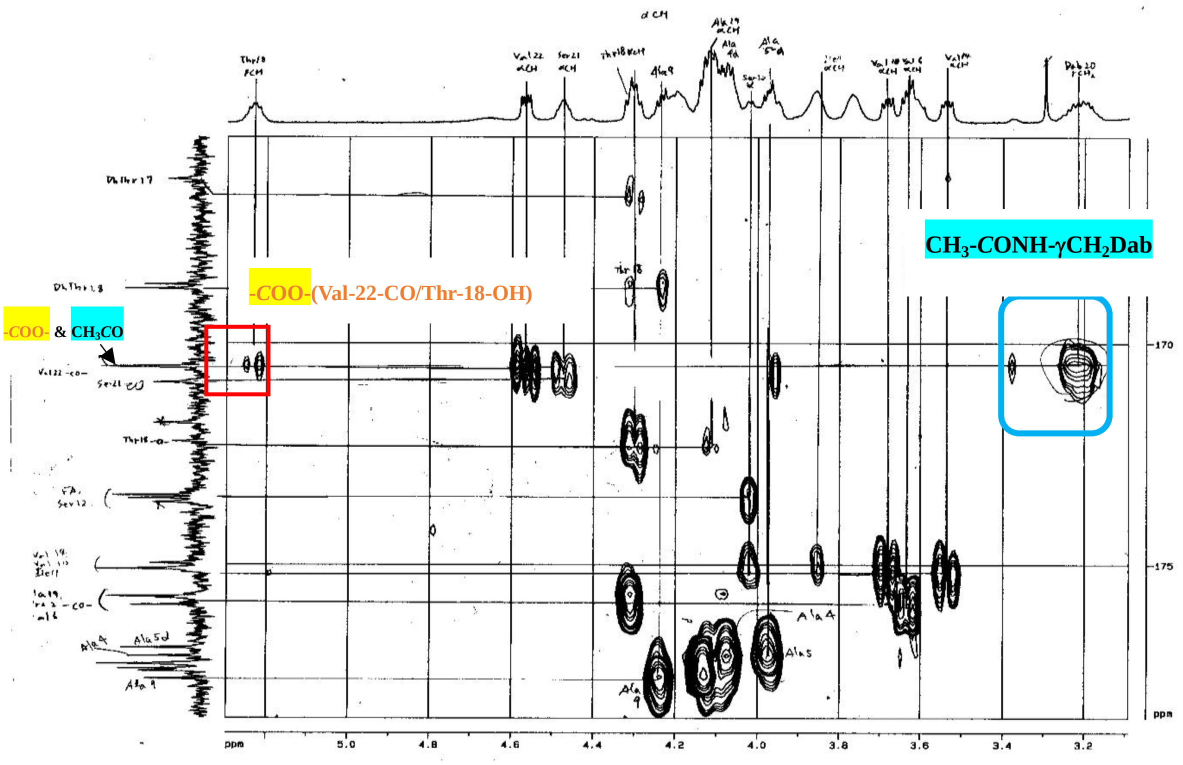

Figure 12B: HMBC spectrum of the monoacetate (ester and NH-Ac regions are highlighted).

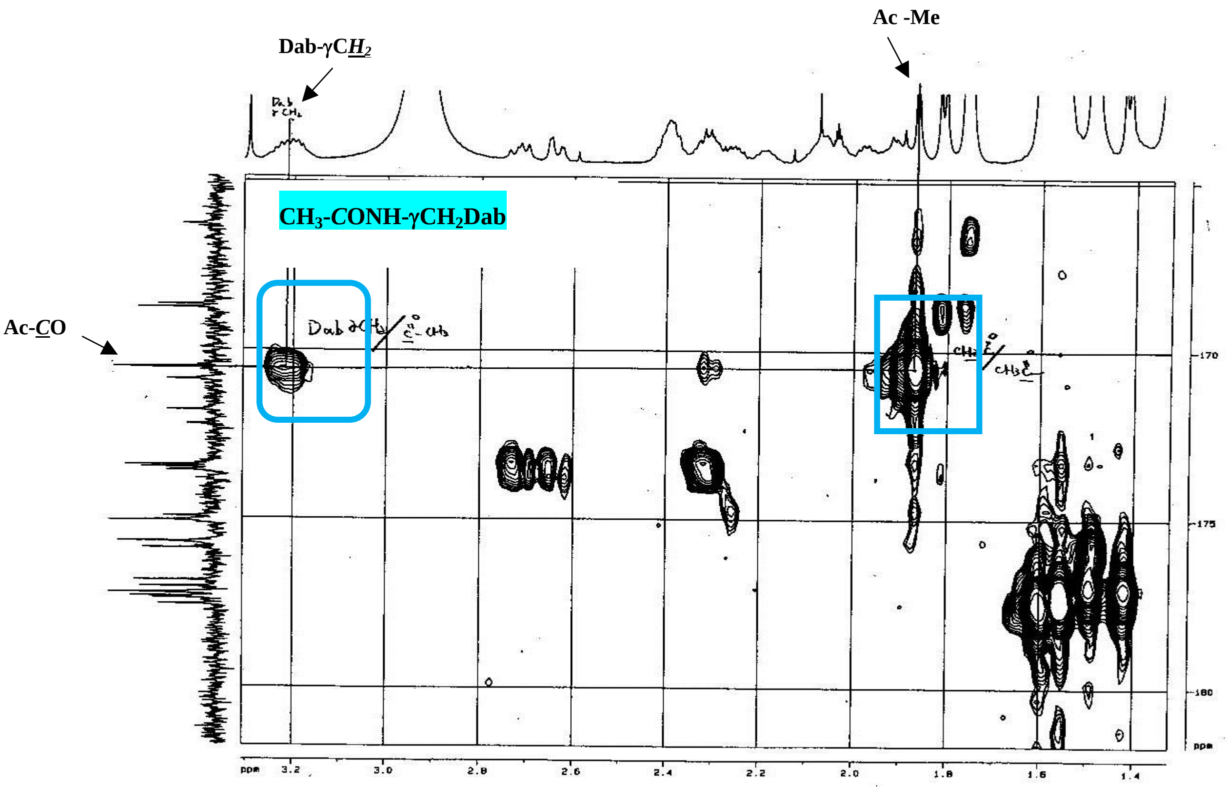

Figure S12C: HMBC spectrum for the assignment of acetyl Me signal of the monoacetate.

Table S1: ^{1}H NMR data of cichorinotoxin monoacetate in acetone-d_6 (600 MHz).
The multiplicity and coupling constants (*J*, Hz) are given only for the protons that were clearly observed, but for other protons, whose multiplicities are not clear, the chemical shifts only are given.

AA	NH	α	β	γ	δ	others
DhThr[1]	9.83 (s)	–	5.89 (q, *J*=7.0 Hz)	C$\underline{H_3}$ 1.82 (d, *J*=7.0 Hz)	–	
Pro[2]	–	4.31	C$\underline{H_2}$ 1.91, 2.39	C$\underline{H_2}$ 2.06, 1.96	C$\underline{H_2}$ 4.06; 3.60	
Ala[3]	7.70 (d, *J*=6.1 Hz)	4.12	C$\underline{H_3}$ 1.55	–	–	
Ala[4]	8.14 (d, *J*=3.3 Hz)	4.06	C$\underline{H_3}$ 1.42 (d, *J*=7.2 Hz)	–	–	
Ala[5]	8.28	3.97	C$\underline{H_3}$ 1.50 (d, *J*=7.2 Hz)	–	–	
Val[6]	7.79	3.62 (m)	2.26 (m)	C$\underline{H_3}$ 0.98 (d, *J*=7.0 Hz); C$\underline{H_3}$ 1.14	–	
Ala[7]	8.30	4.09	1.61 (*d*, *J*=7.3 Hz)	–	–	
DhThr[8]	9.51 (s)	–	6.24 (q, *J*=7.0 Hz)	C$\underline{H_3}$ 1.76 (d, *J*=7.0 Hz)	–	
Ala[9]	8.07 (d, *J*=3.5 Hz)	4.23	C$\underline{H_3}$ 1.59 (d, *J*=7.3 Hz)	–	–	
Val[10]	8.10 (d, *J*=3.5 Hz)	3.68 (dd, *J*=10.4, 5.4 Hz)	2.39	C$\underline{H_3}$ 0.96; C$\underline{H_3}$ 1.12	–	
Ile[11]	8.14 (d, *J*=3.3 Hz)	3.85 (bs)	1.98 (m)	C$\underline{H_3}$ 0.99 C$\underline{H_2}$ 1.22; 1.54	C$\underline{H_3}$ 0.85(t, *J*=7.3 Hz)	
Ser[12]	8.42 (bs)	4.01	3.87, 4.22	–	–	
Ala[13]	8.28	4.12	C$\underline{H_3}$ 1.57	–	–	
Val[14]	8.24 (bs)	3.53 (dd,	2.19 (m)	C$\underline{H_3}$ 0.91;	–	

		J=10.0, 4.9 Hz)		$C\underline{H}_3$ 1.07 (d, *J*=6.3 Hz)		
Ala^{15}	8.20 (bs)	4.10	$C\underline{H}_3$ 1.57	–	–	
Val^{16}	7.83	3.76	2.41	$C\underline{H}_3$ 1.03 (d, *J*=6.5 Hz); $C\underline{H}_3$ 1.16 (d, *J*=6.5 Hz)	–	
DhThr^{17}	8.93 (s)	–	6.37 (q, *J*=7.0 Hz)	$C\underline{H}_3$ 1.75 (d, *J*=7.0 Hz)	–	
Thr^{18}	7.78 (s)	4.29 (m)	5.23 (m)	$C\underline{H}_3$ 1.34 (d, *J*=6.0 Hz)		
Ala^{19}	7.98 (bs)	4.10	$C\underline{H}_3$ 1.48 (d, *J*=7.2 Hz)	–	–	
Dab^{20}	8.61 (bs)	3.76	2.33 (m, 1H); 2.08 (m, 1H)	$C\underline{H}_2$ 3.21 (m, 2H)	–	γN*H* 7.35 (bs) $COC\underline{H}_3$ 1.87 (s)
Ser^{21}	7.82	4.46 (m)	3.94; 4.05	–	–	
Val^{22}	7.60 (d, *J*=9.0 Hz)	4.56 (dd, *J*=9.6, 3.8 Hz)	2.31	$C\underline{H}_3$ 0.87;0.91	–	

* The ^{1}H NMR data of the fatty acid moiety is described in Figure 3 (main Text).

Table S2: ^{13}C NMR data of cichorinotoxin monoacetate in acetone-d_6 (150 MHz).

AA	CO	α	β	γ	δ	Others
DhThr^{1}	168.77 (s)	132.8 (s)	123.3 (d)	12.28 (q)^{g}	–	
Pro^{2}	175.76(s)^{l}	60.6 (t)	30.58 (t)	26.4 (t)	49.9 (t)	
Ala^{3}	*j*	*b*	h ($\underline{C}H_3$)	–	–	
Ala^{4}	177.07	*b*	16.1 (q)	–	–	
Ala^{5}	176.88	*b*	h ($\underline{C}H_3$)	–	–	
Val^{6}	175.9 (s)	65.09 (d)^{d}	*f*	γCH_3: 19.9 (q); 21.2		

				(q)		
Ala^{7}	*j*	*b*	h ($\underline{C}H_3$)	—	—	
DhThr^{8}	168.67 (s)	132.6 (s)[a]	128.2 (d)	12.37 (q)[g]	—	
Ala^{9}	177.57	53.5 (d)	h ($\underline{C}H_3$)	—	—	
Val^{10}	175.1(s)[k]	65.10 (d)[d]	*f*	*i*		
Ile^{11}	173.4 (s)[n]	62.4 (d)	35.9 (d)	26.2 (d), 16.75 (γ CH_3)[o]	11.3 (q)	
Ser^{12}	174.9 (s)[n]	56.6 (d)	61.8 (t)	—	—	
Ala^{13}	*j*	*b*	*h* ($\underline{C}H_3$)	—	—	
Val^{14}	175.1(s) [k]	64.6 (d) [c]	*f*	*i*		
Ala^{15}	*j*	*b*	h (CH_3)	—	—	
Val^{16}	*m*	64.6 (d)[c]	*f*	*i*		
DhThr^{17}	166.3(s)	132.5 (s) [a]	129.3(d)	12.8 (q)	—	
Thr^{18}	172.2 (s)	63.8 (d)	71.8 (d)	18.9 (q)	—	
Ala^{19}	175.72 (s)[l]	51.7 (d)	*h* ($\underline{C}H_3$)	—	—	
Dab^{20e}	*m*	*b*	*f*	37.5 (t)	—	$\underline{C}OCH_3$ 170.53 (or 170.54)[e]; $CO\underline{C}H_3$ 23.0
Ser^{21}	170.9 (s)	57.1 (d)	63.1 (t)	—	—	
Val^{22}	170.53 (or 170.54)[e]	56.9 (d)	30.45 (t)	γ$\underline{C}H_3$: 18.0 (q); 20.2 (q)	—	

a: The chemical shifts of *a* may be exchangeable due to closely spaced peaks.

b. Assignments of each of b peaks were unsuccessful, because these peaks were closely spaced peaks: δ_C (ppm) 53.28. 53.30, 53.37, 53.71, 53.76, 53.81, 54.33.

c: the two carbon signals were overlapped.

d: The chemical shifts of *a* may be exchangeable due to closely spaced peaks.

e: δNH-*C*O*C*H3. The chemical shifts of *C*H_3 was 23.0 ppm, and that of *C*O was assigned to be either 170.53 or 170.54.

f: The carbon signals (*C*H) at β-position of Val moieties were buried in the large solvent peaks (acetone-d_6) that appeared around 29.8 ppm, thus, the exact chemical shifts were

not given.

g: exchangeable.

h: The Me signals, which are assigned to be Ala βCH_3, were resonated at δ_C (ppm)16.1, 16.3, 16.43, 16.47, 16.59, 16.63 and 17.1. The signal peak at δ_C 16.63 was larger (ca, twice) than those of other Ala βCH_3, indicating that two AlaβCH_3 carbon signals were overlapped.

i: The Me signals were resonated at δ_C (ppm) 19.67, 19.70, 19.73, 19.8 and 20.2 These carbon signals (Val γCH_3) were not differentiated due to the closed peaks in the HMBC spectrum.

j: The carbonyl carbons of 8 Ala moieties were found at δ_C (ppm) 177.57, 177.40, 177.35, 177.24, 177.07 and 176.88. The 6 carbonyl carbon signals were observed, indicating that the other 2 carbons were overlapped at the above-mentioned CO signals. δ_C 176.88 was confirmed to be <u>*C*</u>O of Ala 5 by a clear HMBC cross peak between αC<u>*H*</u> of Ala 5 and the carbonyl signal. The signal δ_C 177.07 was assigned to be <u>*C*</u>O of Ala 4, because clear HMBC cross peak was observed between βC<u>*H*</u>$_3$ of Ala 4 and δ_C 177.07. The signal δ_C 177.57 was assigned to be <u>*C*</u>O of Ala 9, because clear HMBC cross peak was observed between αC<u>*H*</u> of Ala 9 and δ_C 177.57. Assignments of other j peaks were unsuccessful, because these peaks were closely situated.

k: The two peaks were overlapped.

l: exchangeable due to the closely spaced signals

m: the two carbonyl carbons were invisible in the ^{13}C NMR and in the HMBC spectrum, thus the δ_C (ppm) of the two carbons were not given.

n: Remark: αCH proton of Ser12 had unambiguous HMBC cross peaks for the two carbonyl carbons at 174.9 and 173.7 ppm. Observation of the HMBC correlations of αCH and βCH of Ser 12 for the carbon signal at 174.9 ppm, but no cross peak for the carbon at 173.4 ppm, enabling the differentiation between the assignments of the carbonyl groups of Ile11 and Ser 12.

o: the assignment was confirmed by a clear HMBC cross peak between Ile11αCH proton and the carbon signal at δ_C 16.75.

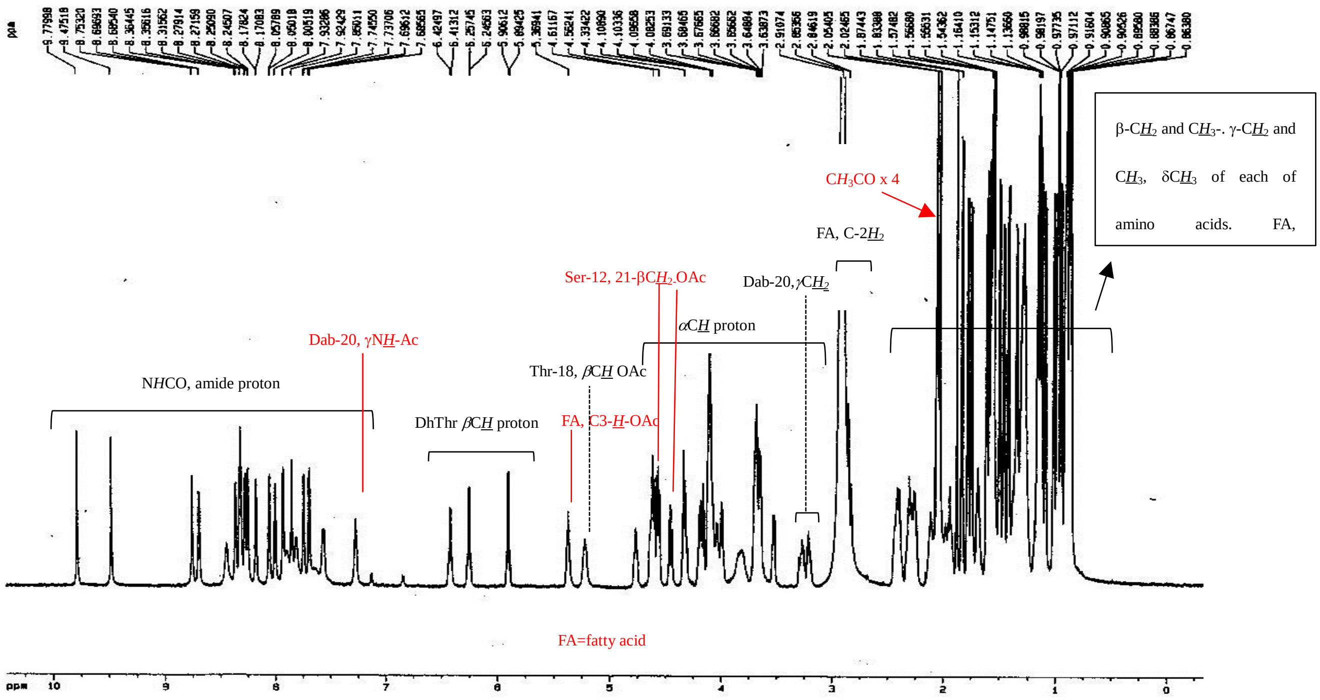

Figure S13A: ^{1}H NMR spectrum of cichorinotoxin tetraacetate (600 MH, acetone-d_6 overall spectrum).

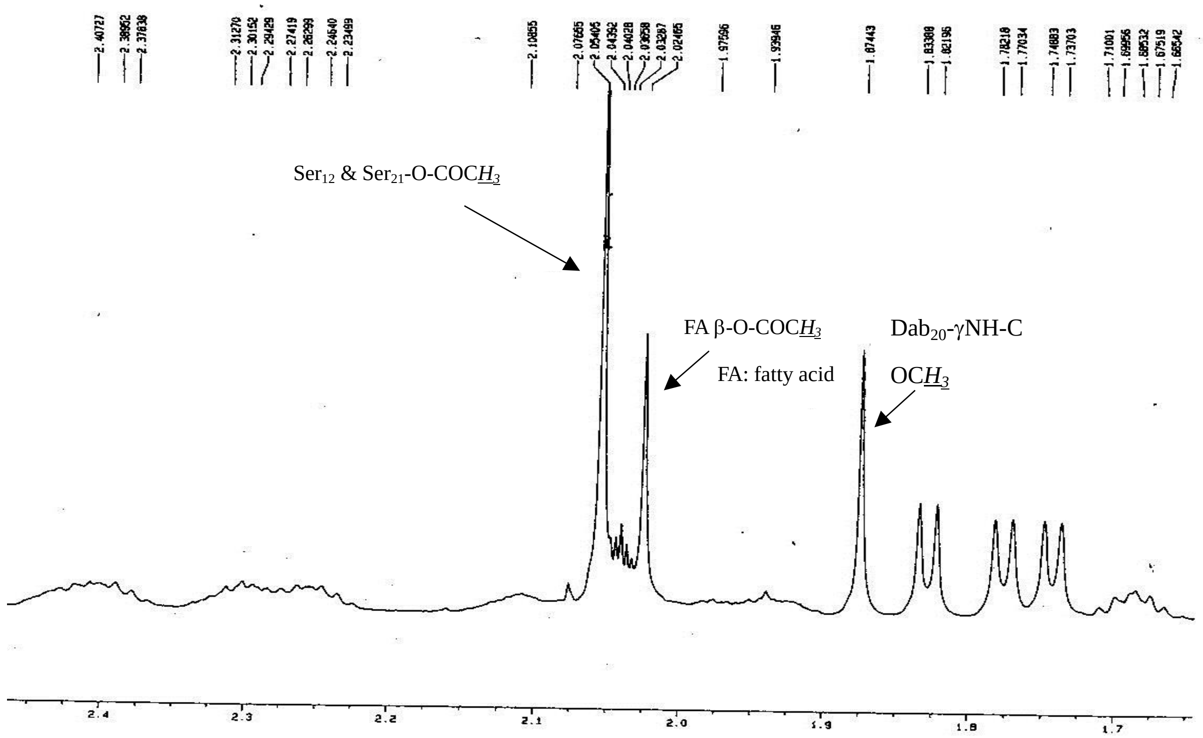

Figure S13B: ^{1}H NMR spectrum of cichorinotoxin tetraacetate (acetyl protons region).

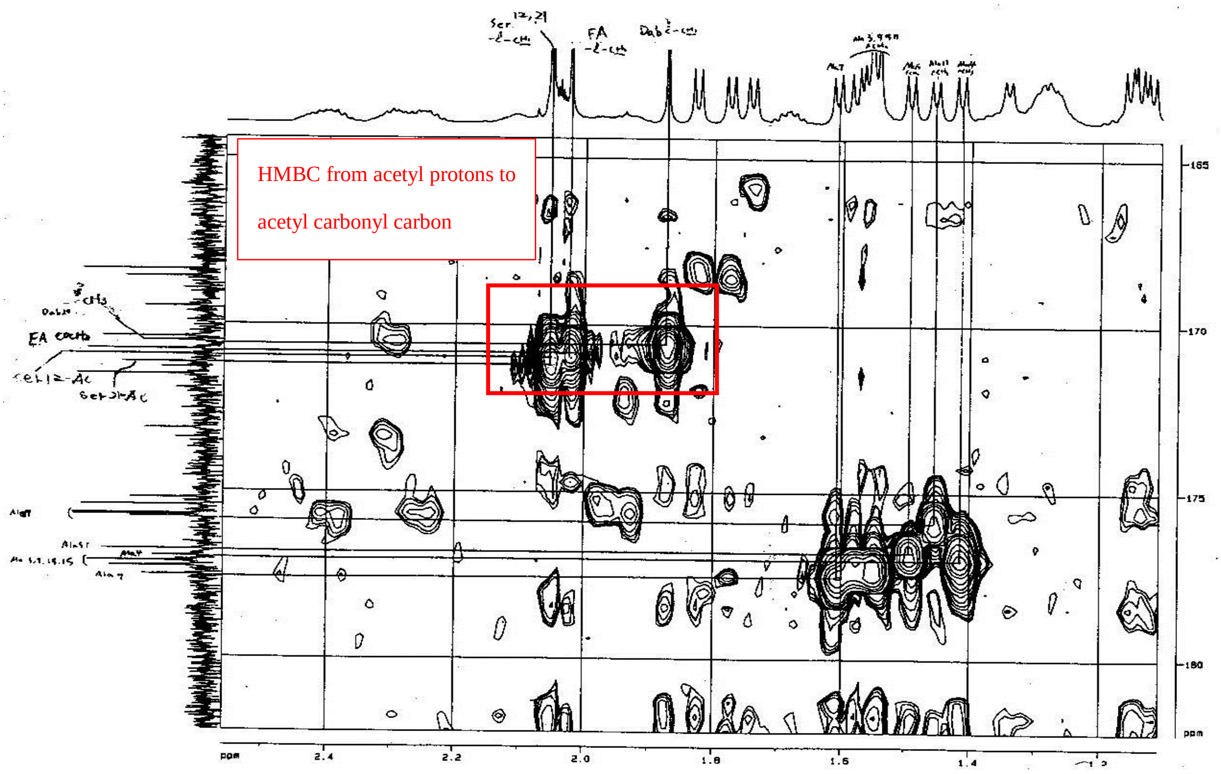

Figure S13C: HMBC spectrum of the tetraacetate: the HMBC cross peaks from acetyl Me proton to acetyl carbon are highlighted).

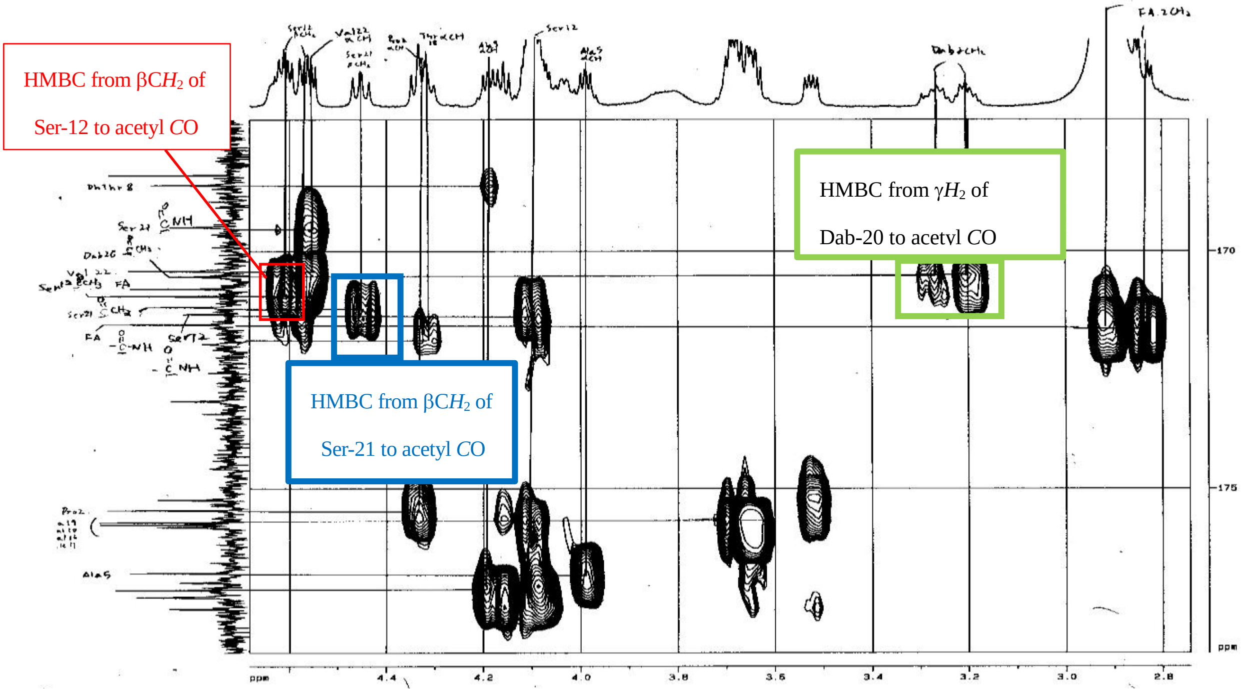

Figure S13D: HMBC spectrum of the tetraacetate: the HMBC data indicated that the OH groups of Ser-12, Ser-21 and γ-NH_2 group of Dab-20 were acetylated.

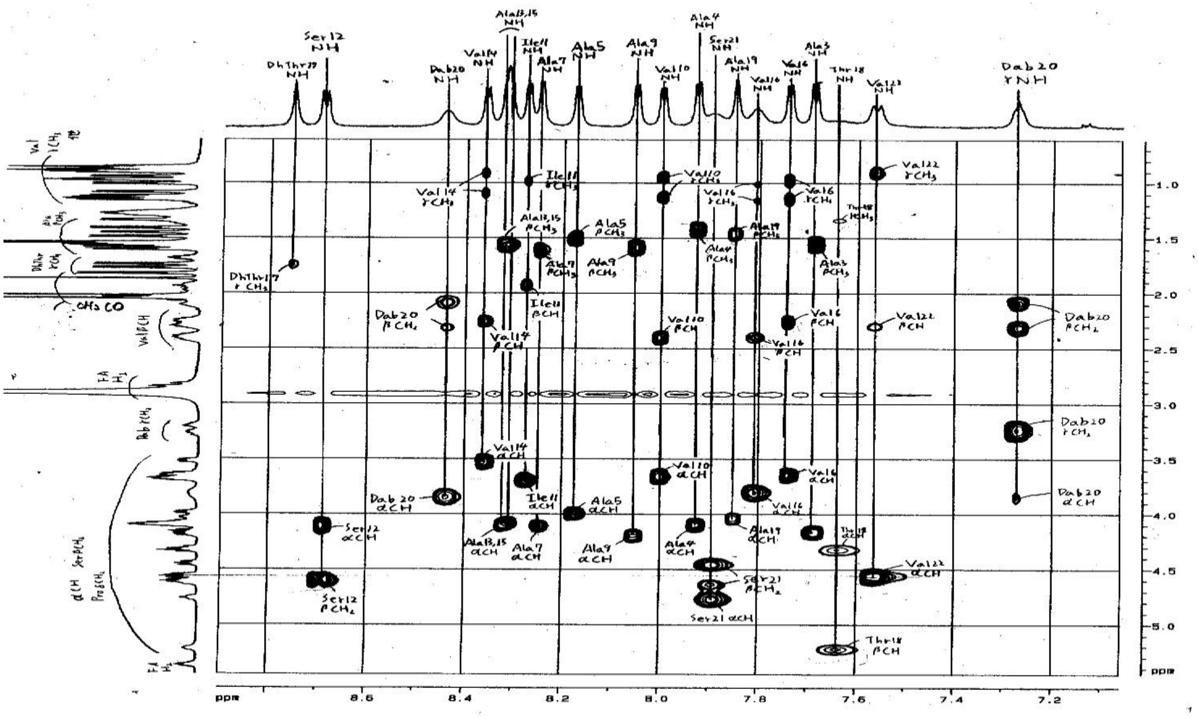

Figure S13E: TOCSY spectrum of the tetraacetate, where the TOCSY correlations from the amide NH protons to aliphatic methyl protons are highlighted.

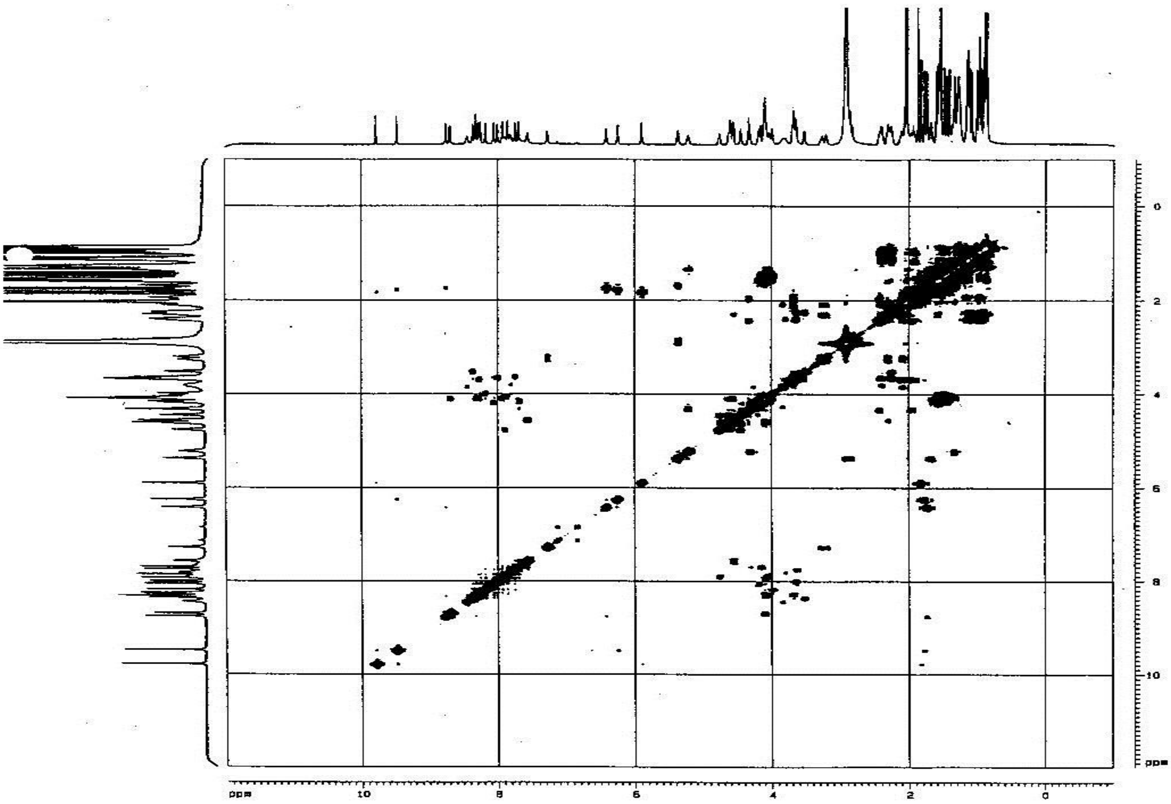

Figure S13F: $^{1}H,^{1}H$ COSY spectrum of the tetraacetate (overall region).

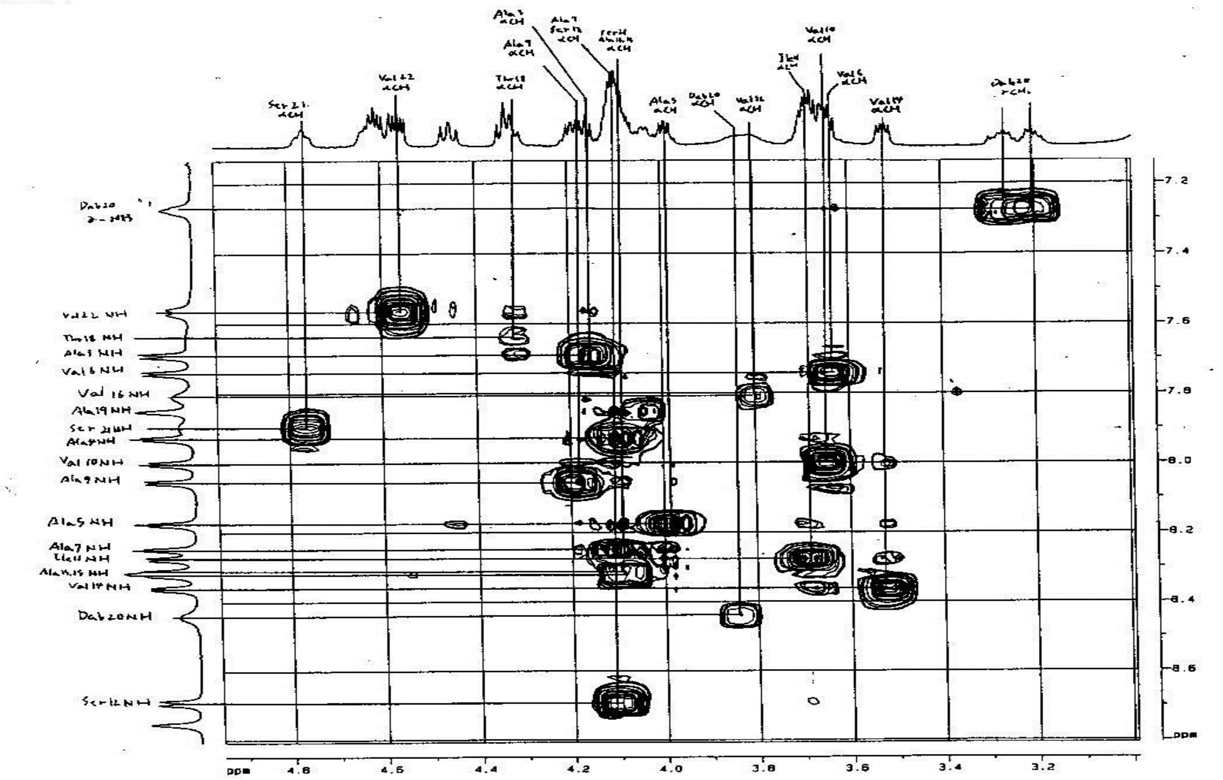

Figure S13G: ^{1}H,^{1}H COSY spectrum of the tetracetate (N^{i}H/Cα^{i}H cross peaks regions are highlighted).

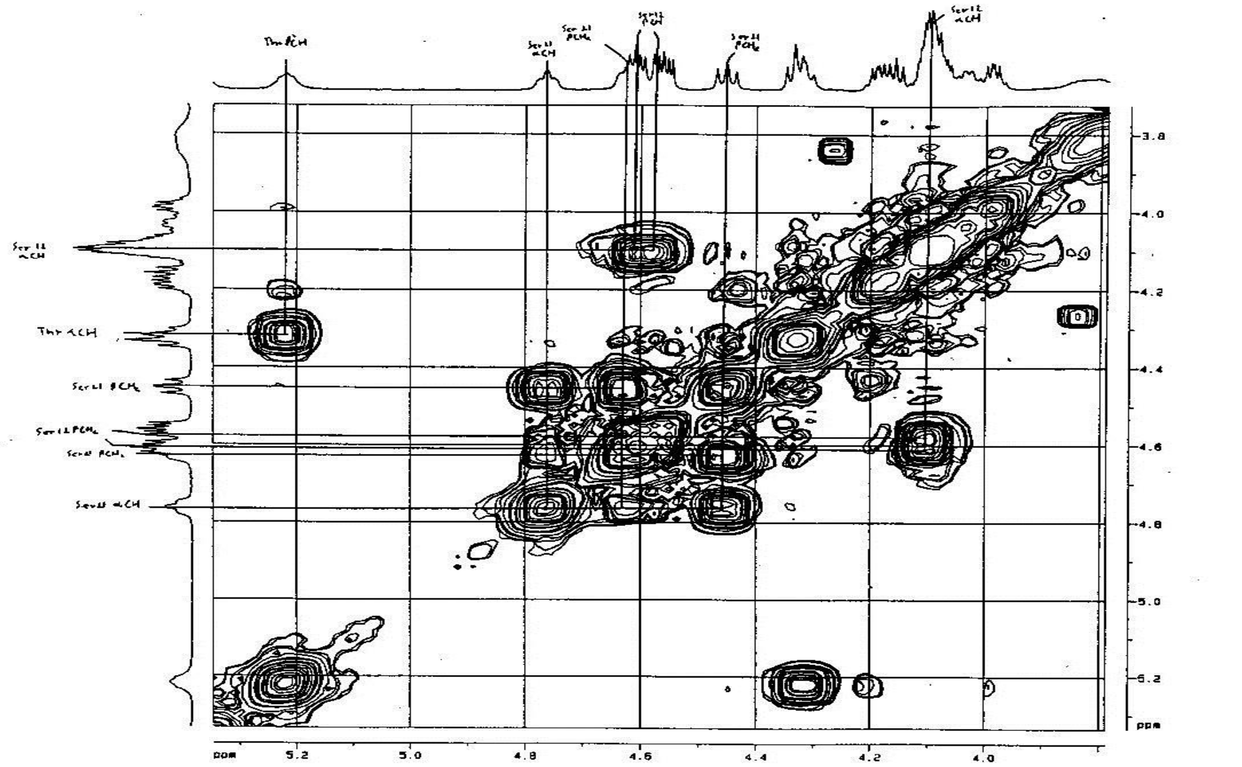

Figure S13H: ^{1}H,^{1}H COSY spectrum of the tetracetate (the cross peaks of Cα^{i}H /Cβ^{i}H of Ser and Thr are highlighted).

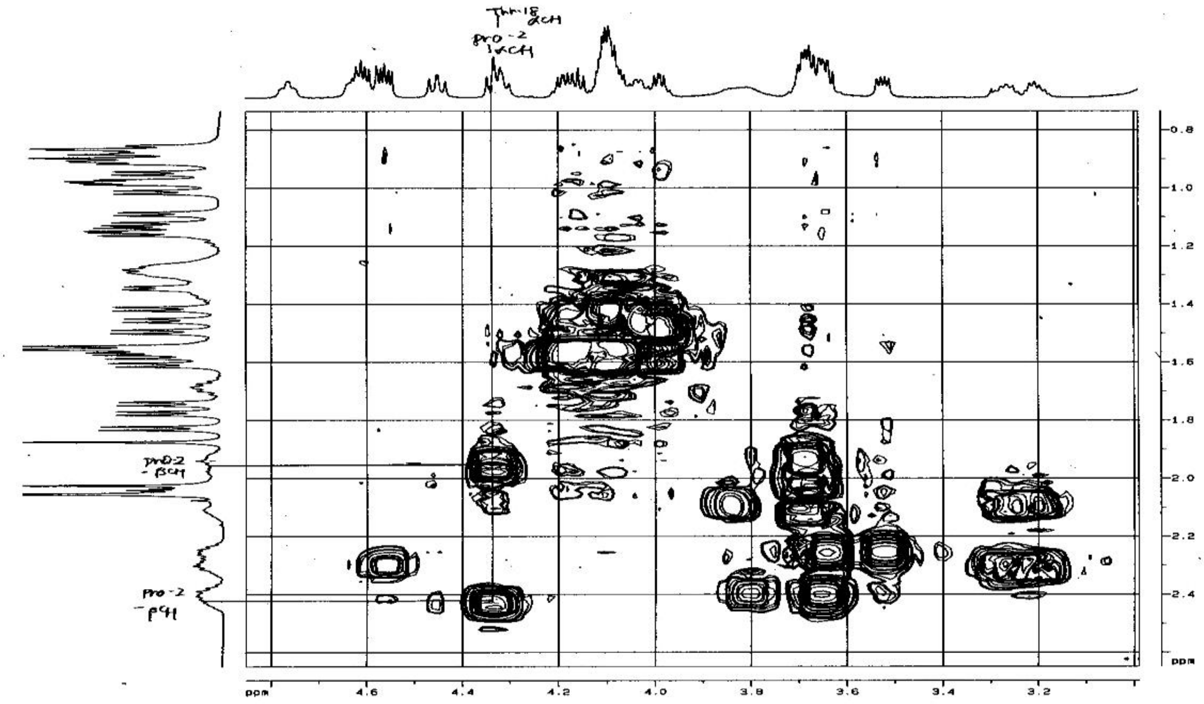

Figure S13I: ^{1}H,^{1}H COSY spectrum of the tetracetate (the cross peaks of Cα'H /Cβ'H of Pro-2 are highlighted).

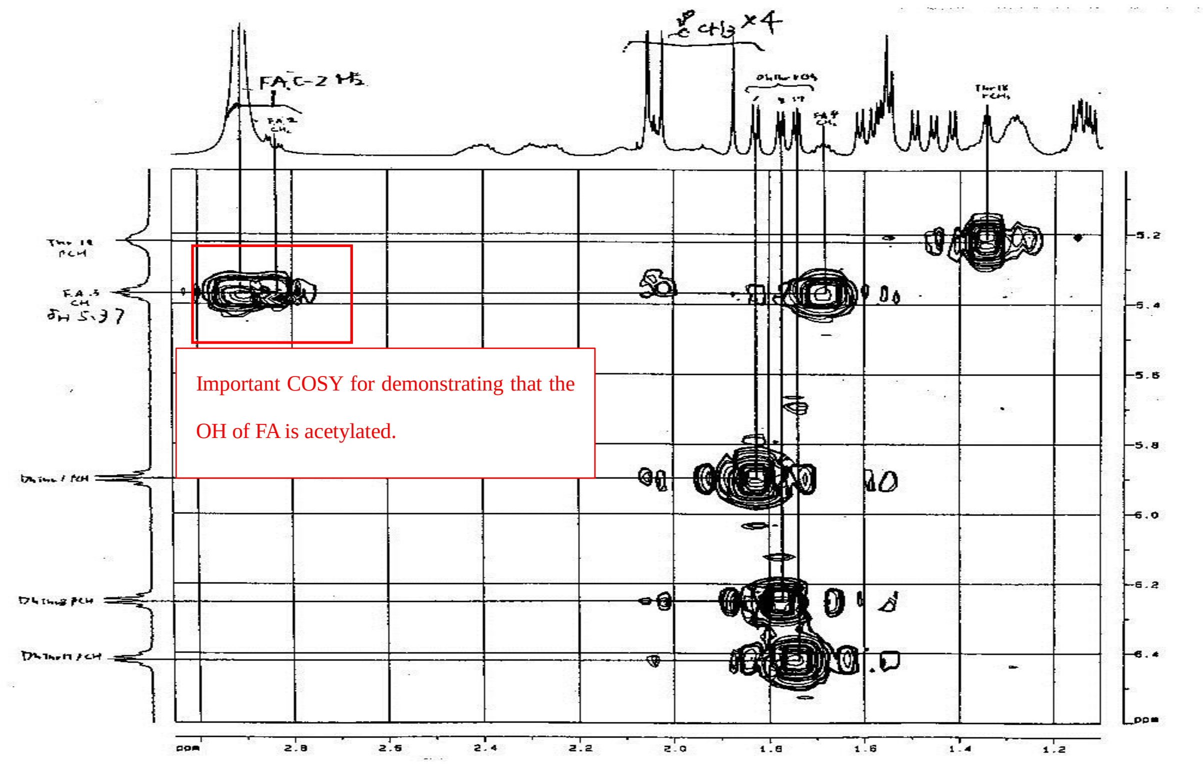

Figure S13J: ^{1}H,^{1}H COSY spectrum of the tetracetate (the cross peaks of DhThr β-Me/β-CH are highlighted). The clear COSY cross peak of FA C-2 CH_2/ FA C-3 CH enabled the assignment of FA C-3 CH (δ_H 5.37), demonstrating that the OH of FA was acetylated.

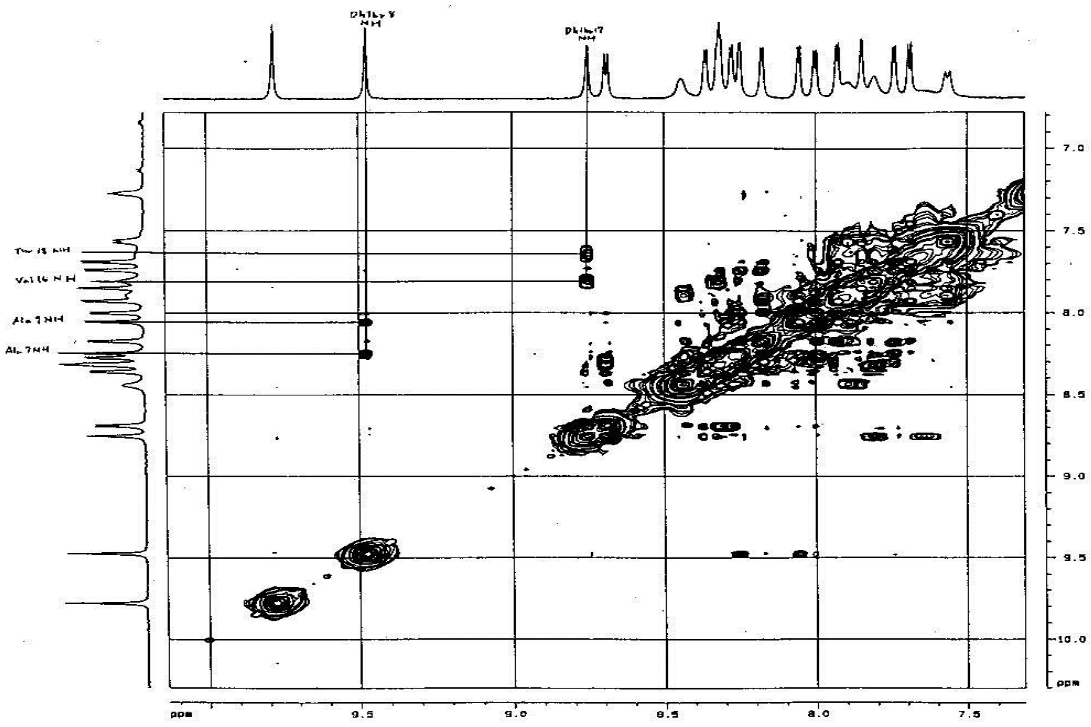

Figure S13K: NOE correlations for $\alpha N^{i}H/\alpha N^{i+1}H$.

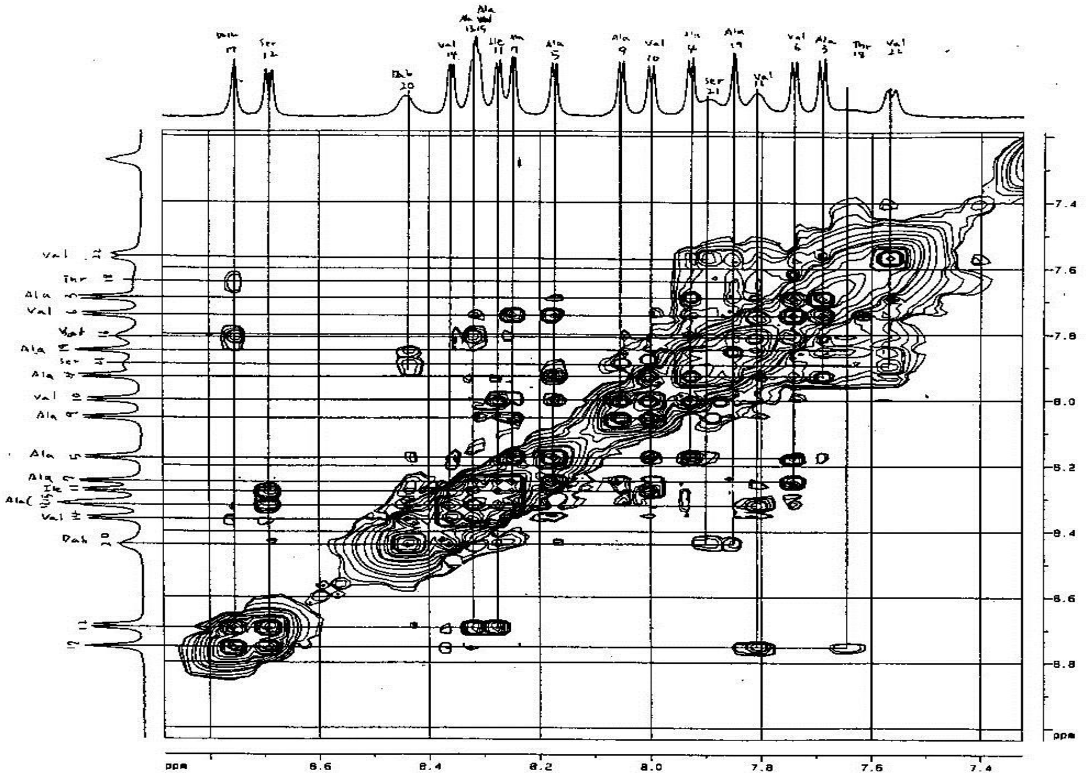

Figure S13L: NOESY correlations for $\alpha N^{i}H/\alpha N^{i+1}H$ (expanded region of Figure S13K).

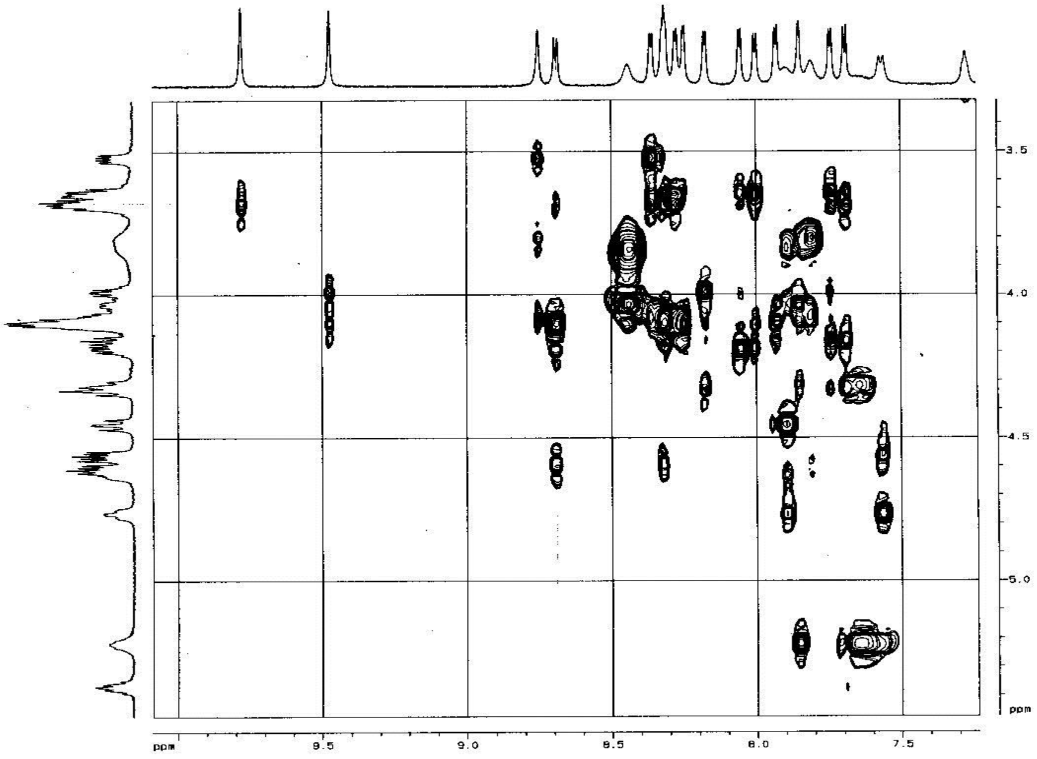

Figure S13M: NOESY correlations for $\alpha N^{i}H/\alpha C^{i}H$.

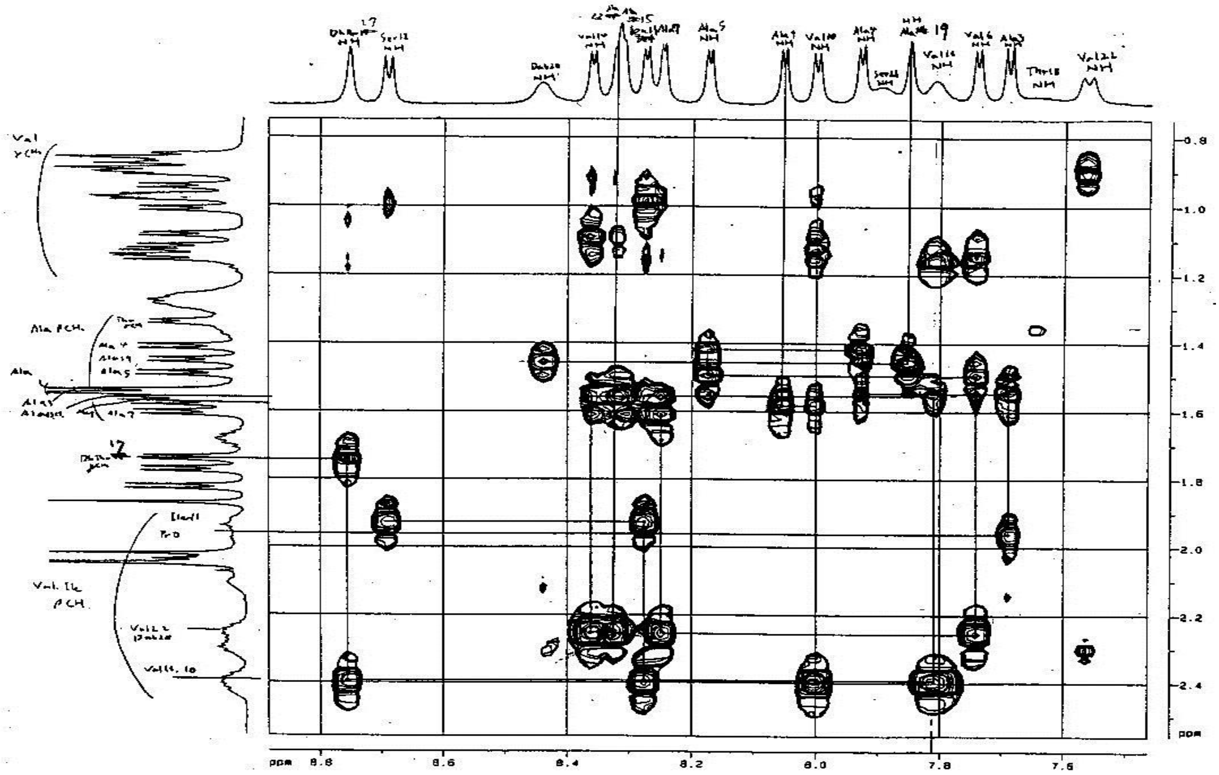

Figure S13N: NOESY correlations ($\beta C^{i}H/\alpha N^{i+1}H$ cross peaks are highlighted).

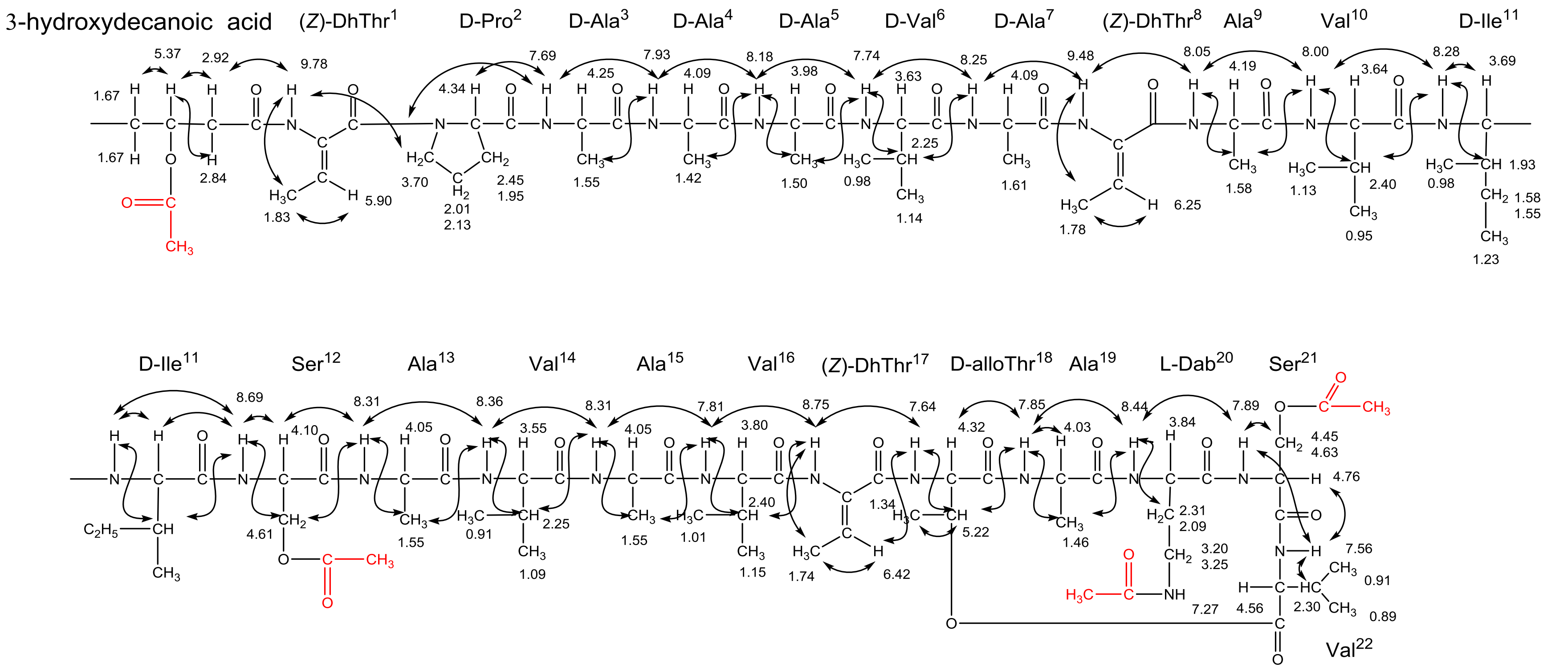

Figure S 14: Summarized NOE correlations and the ^{1}H-chemical shifts of the tetraacetate (600 MHz, acetone-d_6). The NOE correlations are collected from the Figures S13K–S13M.

Table S3: ^{1}H NMR chemical shifts of the tetraacetate in acetone-d_6 (600 MHz). The multiplicity and coupling constants (*J*, Hz) are given only for the protons that were clearly observed, but for other protons, whose multiplicities are not clear, the chemical shifts only are given.

AA	NH	α	β	γ	δ	others
DhThr[1]	9.78 (s)	–	5.90 (q, *J*=7.0 Hz)	C*H*$_3$ 1.83 (d, *J*=7.0 Hz)	–	
Pro[2]	–	4.34	C*H*$_2$ 2.45, 1.95	C*H*$_2$ 2.01, 2.13	C*H*$_2$ 3.70	
Ala[3]	7.69 (d, *J*=6.3 Hz)	4.25	C*H*$_3$ 1.55	–	–	
Ala[4]	7.93 (d, *J*=5.1 Hz)	4.09	C*H*$_3$ 1.42 (d, *J*=7.3 Hz)	–	–	
Ala[5]	8.18 (d, *J*=4.5 Hz)	3.99	C*H*$_3$ 1.50 (d, *J*=7.2 Hz)	–	–	
Val[6]	7.74 (d, *J*=5.1 Hz)	3.63 (m)	2.25 (m)	C*H*$_3$ 0.98; C*H*$_3$ 1.14	–	
Ala[7]	8.25 (d, J=3.5 Hz)	4.09	1.61 (*d*, *J*=7.2 Hz)	–	–	
DhThr[8]	9.48 (s)	–	6.25 (q, *J*=7.0Hz)	C*H*$_3$ 1.78 (d, *J*=7.0 Hz)	–	
Ala[9]	8.05 (d, *J*=4.6 Hz)	4.19	C*H*$_3$ 1.58 (d, *J*=7.3 Hz)	–	–	
Val[10]	8.00 (d, *J*=5.5 Hz)	3.64	2.40	C*H*$_3$ 0.95; C*H*$_3$ 1.13	–	
Ile[11]	8.28 (d, *J*=4.5 Hz)	3.69	1.94 (m)	C*H*$_3$ 0.98 C*H*$_2$ 1.58;1.55	C*H*$_3$ 0.98	
Ser[12]	8.69 (d, *J*=6.9 Hz)	4.10	4.61 (2H)	–	–	COC*H*$_3$ 2.08 (s)
Ala[13]	8.31	4.05	C*H*$_3$ 1.55	–	–	
Val[14]	8.36 (d, J=5.0 Hz)	3.55 (dd, *J*=10.0, 5.0 Hz)	2.25 (m)	C*H*$_3$ 0.91; C*H*$_3$ 1.09 (d, *J*=6.4 Hz)	–	
Ala[15]	8.31	4.05	C*H*$_3$ 1.57	–	–	
Val[16]	7.81 (bs)	3.80	2.40	C*H*$_3$ 1.01;	–	

				C*H*3 1.15		
DhThr[17]	8.75 (s)	–	6.42 (q, *J*=7.1 Hz)	C*H*3 1.74 (d, *J*=7.1 Hz)	–	
Thr[18]	7.64 (very broad)	4.32 (m)	5.22 (very broad)	C*H*3 1.34		
Ala[19]	7.85 (s)	4.09	C*H*3 1.45 (d, *J*=7.0 Hz)	–	–	
Dab[20]	8.44 (bs)	3.84	2.31 (m); 2.09 (m)	C*H*2 3.20 (m); 3.25 (m)	–	γN*H* 7.27 (bs) COC*H*3 1.87 (s)
Ser[21]	7.89 (very broad)	4.76 (m)	4.45; 4.63	–	–	COC*H*3 2.08 (s)
Val[22]	7.56 (d, *J*=8.5 Hz)	4.56 (m)	2.30	C*H*3 0.89;0.91	–	

* The ^{1}H NMR chemical shifts of the fatty acid moiety were as follows. H-2, δ_H 2.84, 2.92; H-3, δ_H 5.37; H-4, δ_H 1.67 (2H); H-5, δ_H 1.27; H-6–H-8 not assignable; H-9, δ_H 1.28, 1.55; H-10 (Me), δ_H 0.87. CH_3CO: acetyl proton δ_H 2.02 (see Fig. S13B).

Table S4: ^{13}C-NMR data of cichorinotoxin tetracetate in acetone-d_6 (150 MHz).

AA	CO	α	β	γ	δ	Others
DhThr[1]	168.39	*f*	123.43	*g*	–	
Pro[2]	175.46	*c*	30.46	26.27	49.80	
Ala[3]	*k*	*a*	*d*	–	–	
Ala[4]	*k*	*a*	*d*	–	–	
Ala[5]	176.79	*a*	*d*	–	–	
Val[6]	*j*	65.05	*b*	*h; i*		
Ala[7]	177.54	54.31	*d*	–	–	
DhThr[8]	168.60 (s)	*f*	128.00	*g*	–	
Ala[9]	*k*	*a*	*d*	–	–	
Val[10]	*j*	65.36	*b*	*h; i*		
Ile[11]	*j*	*c*	35.69	CH_2: *e*	11.20	

				CH_3: *d*	(δ CH_3)	
Ser[12]	171.32	*a*	62.02	–	–	$\underline{C}OCH_3$: 170.94; $CO\underline{C}H_3$: *i*
Ala[13]	*k*	*a*	*d*	–	–	
Val[14]	175.24	64.78	*b*	*h; i*		
Ala[15]	*k*	*a*	*d*	–	–	
Val[16]	173.15	64.48	*b*	*h; i*	–	
DhThr[17]	165.84	*f*	128.98	12.79	–	
Thr[18]	171.95	59.98	71.68	18.85	–	
Ala[19]	*j*	51.78	*d*	–	–	
Dab[20e]	173.15	*a*	*b*	37.16	–	$\underline{C}OCH_3$: 170.53; $CO\underline{C}H_3$:23.00
Ser[21]	169.49	*a*	64.48	–	–	$\underline{C}OCH_3$: 171.17; $CO\underline{C}H_3$: *i*
Val[22]	170.41	57.08	30.27	γ$\underline{C}H_3$: *h*; 18.07;	–	

[*] The ^{13}C NMR chemical shifts of the fatty acid moiety were as follows. C-1, δ_C 171.54; C-2, δ_C 40.97; C-3, δ_C 71.68; C-4, *e*; C-5, δ_C 34.94; C-6, *b*; C-7, not assignable; C-8 or C-9, δ_C 23.20 or 32.48; C-10, δ_C 12.79. $CH_3\underline{C}O$: δ_C 170.79; $\underline{C}H_3CO$: δ_C 20.90.

** a–k: the δ_C of these signals were very close each other, thus the correct assignments were unsuccessful.

a: δ_C 53.23, 53.29, 53.46, 53.79, 53.86.

b: overlapped with solvent peak

c: δ_C 63.85, 63.95

d: δ_C 16.14, 16.30,16.38, 16.52, 16.60, 16.67, 16.77, 17.02

e: δ_C 25.89, 26.03

f: δ_C 132.44, 132.52, 132.74

g: δ_C 12.20, 12.31

h: δ_C 19.62, 19.67, 19.73, 19.86, 20.15

i: δ_C 20.82, 20.90, 21.13, 21.17, 21.20

j: δ_C 175.82, 175.76, 175.82

k: δ_C 177.14, 177.29, 177.54

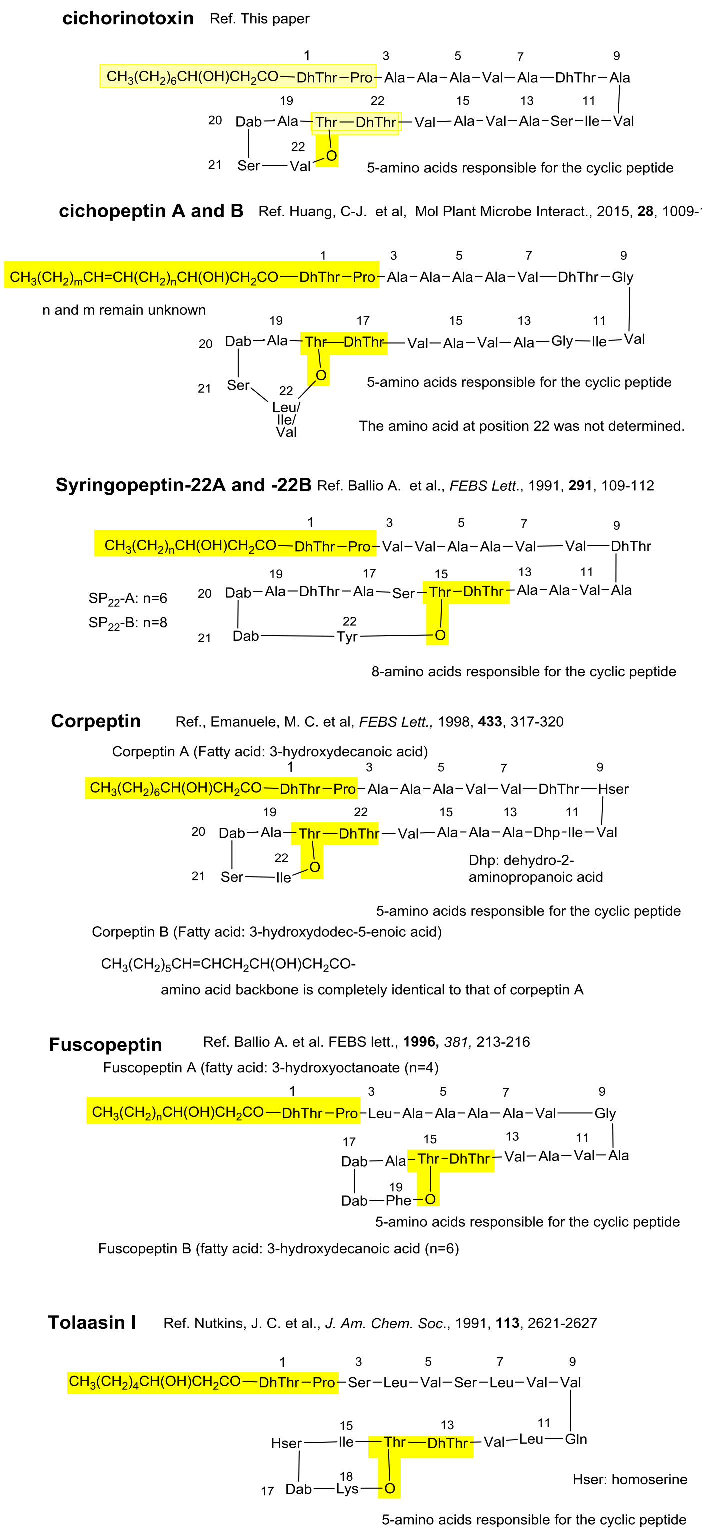

Figure S15: Typical lipodepsipeptides having biological activities. The lipophilic residues (fatty acids) connected with DhThr-Pro are conserved. The two amino acids─DhThr-Thr─are strictly conserved and the OH of the Thr residue is connected to other amino acids through ester bond. The conserved portions are highlighted with yellow background.

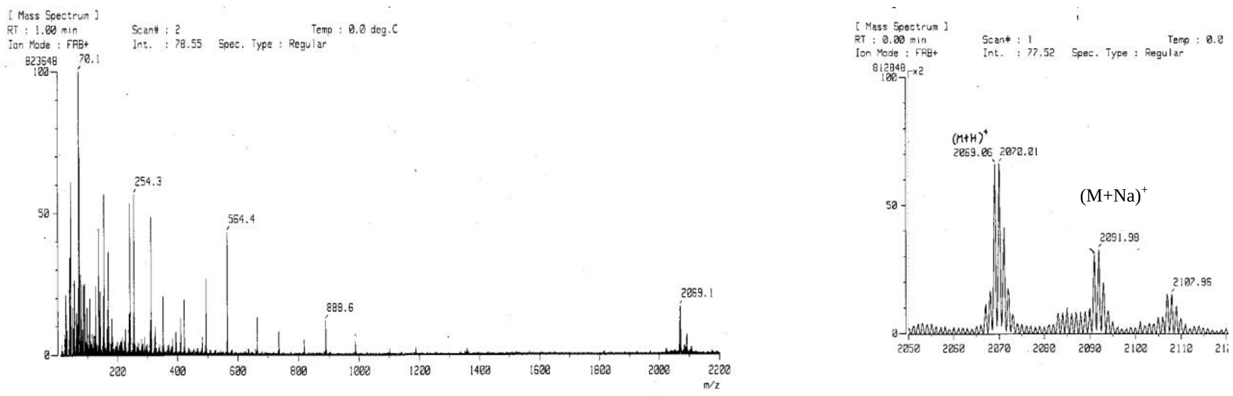

Figure S16A: FABMS spectrum of compound **A** (matrix: NBA); This FABMS data indicated the MW of compound **A** is the same as that of original cichorinotoxin. Remark: $[M + H]^+$ and $[M + Na]^+$ were observed, in a similar was as Figures S4A.

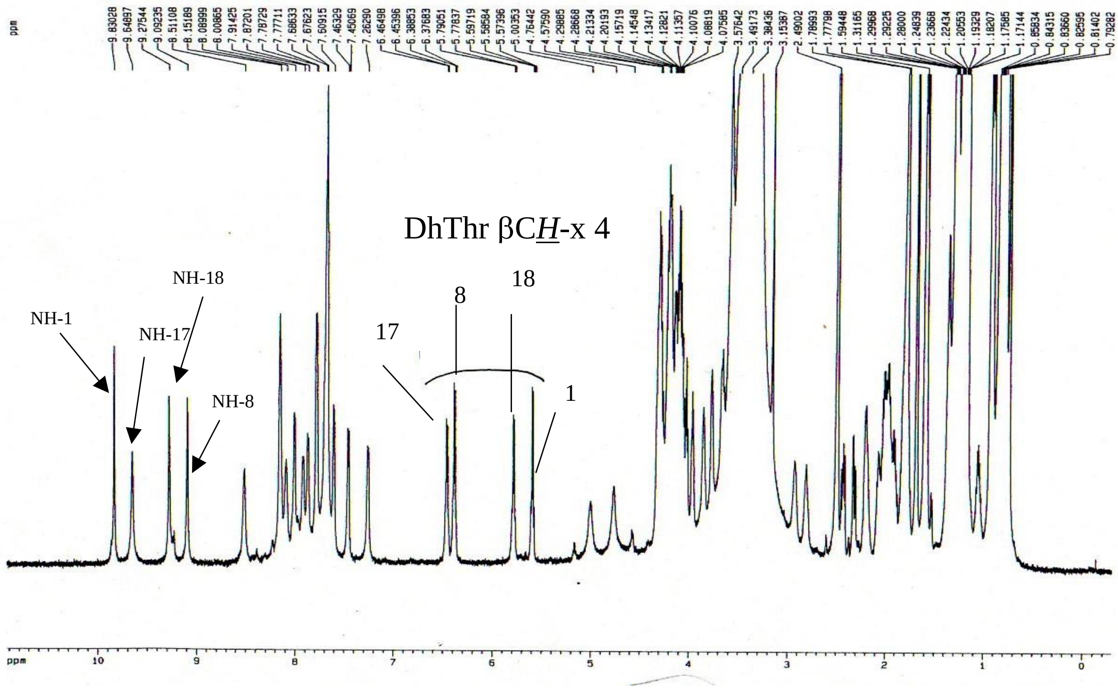

Figure S16B: ^{1}H-NMR of compound **A** (600 MHz, DMSO-d_6). Remark: 4 βC<u>H</u> protons of DhThr were observed, despite 3 βC*H* protons of DhThr are present in the original chicorinotoxin (compare Figure S6 with Figure S16B). The numbers of the amide protons (N*H*) and the olefinic protons (βC*H* proton) indicate the positions of amino acids in the cichorinotoxin.

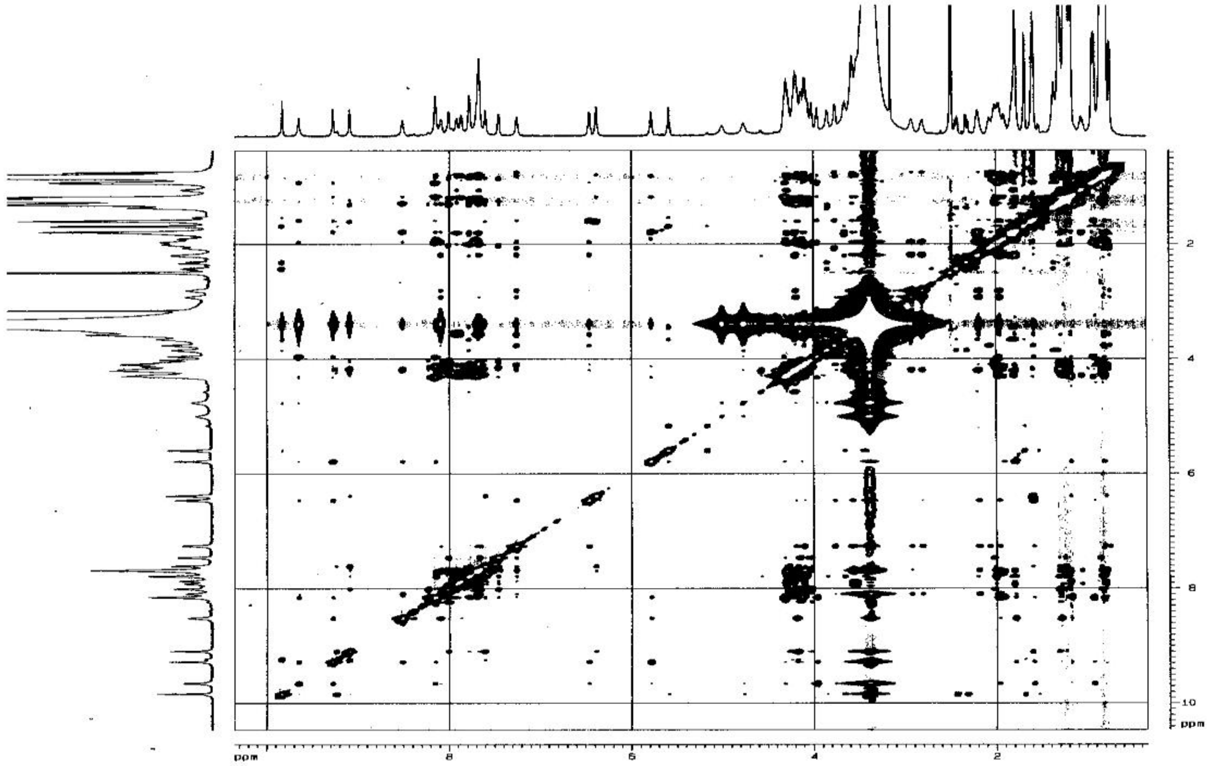

Figure S16C: Overall NOESY spectrum of compound **A** (600 MHz, DMSO-d_6).

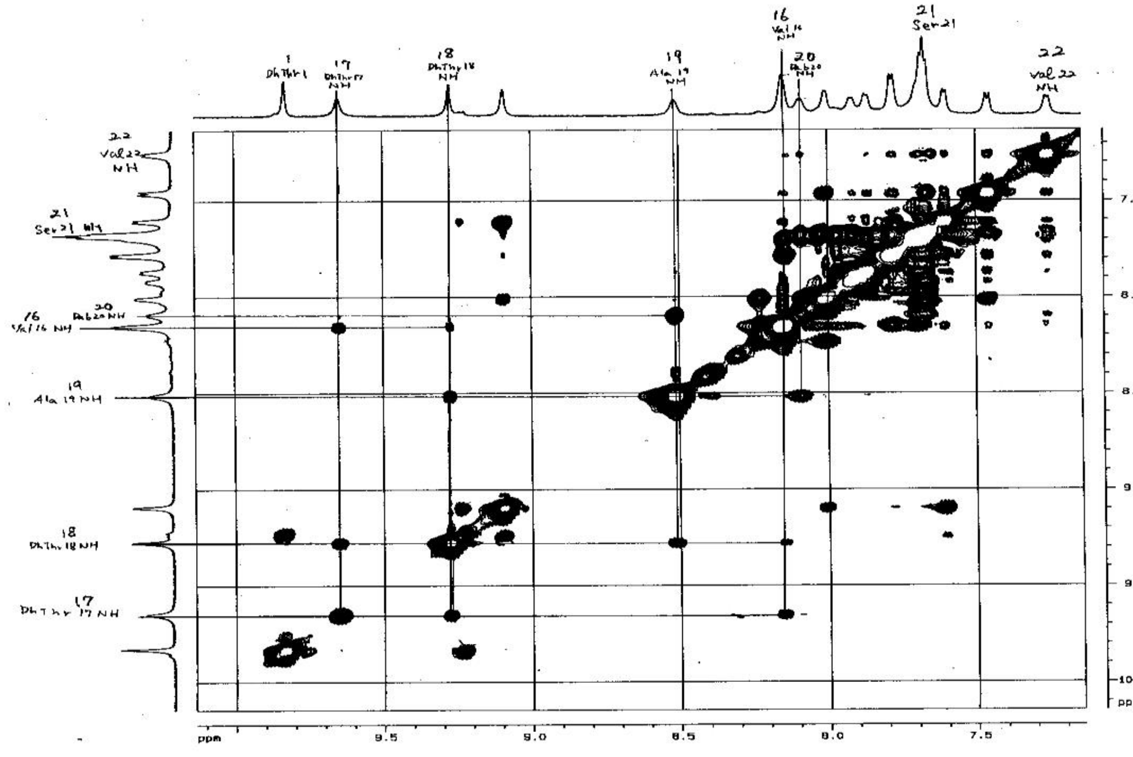

Figure S16D: NOESY correlations for $\alpha N^{i}H/\alpha N^{i+1}H$ (expanded region of Figure S16B).

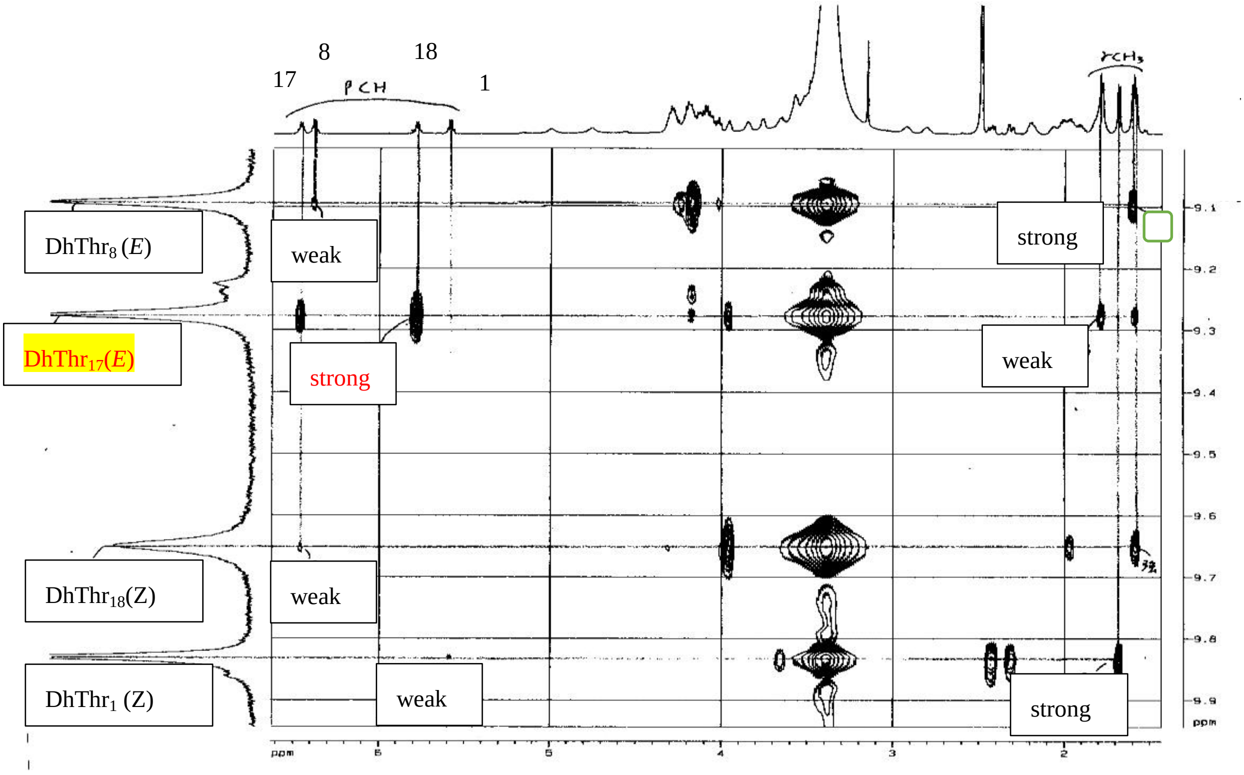

Figure S16E: NOESY correlations for determining the *E*,*Z*-geometry of DhThr of compound **A**.

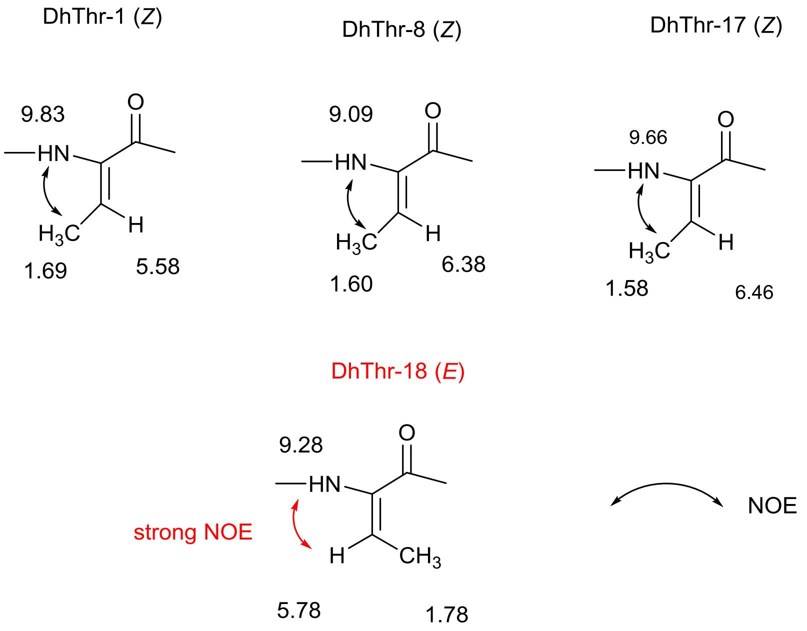

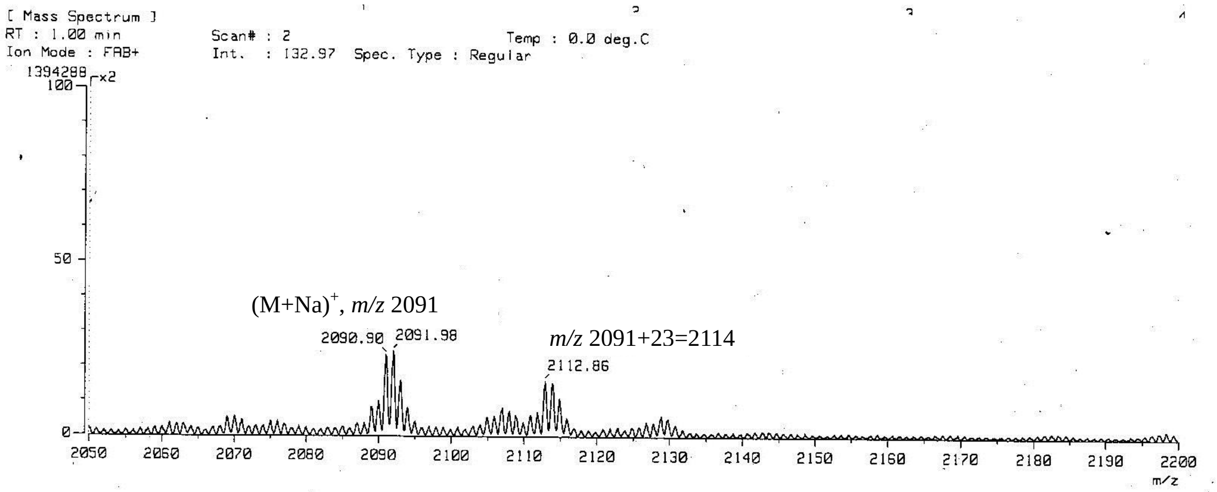

Figure S17A: FABMS spectrum of compound **B** (matrix: NBA). The ions, *m/z* 2091 and *m/z* 2114, were observed, but no peak $[M + H]^+$ (*m/z* 2069) was detected, despite the $[M + H]^+$ ion having been observed for compound **A**. This phenomenon was also observed in the FABMS spectrum of Figure S4B. The careful NMR analyses of compound **B** including 2D NMR indicated that the MW should be the same as that of cichorinotoxin (MW: 2068).

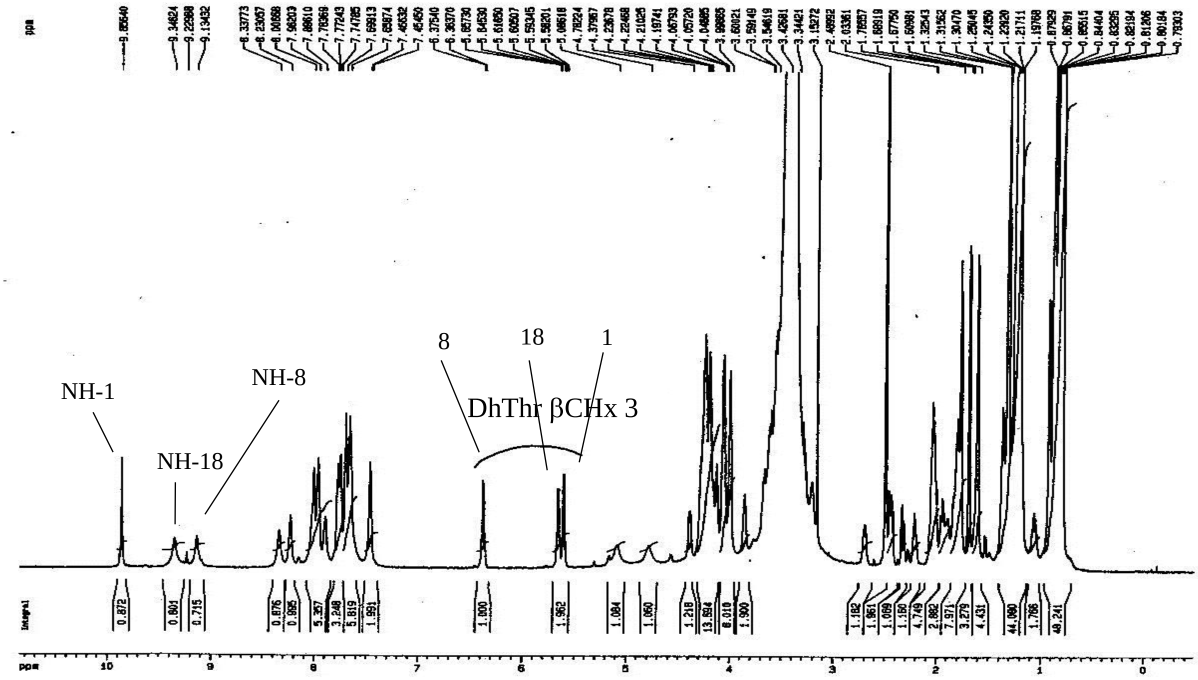

Figure S17B: ^{1}H NMR spectrum of compound **B** (600 MHz, DMSO-d_6). The amide protons (NH) and the olefinic protons (βC<u>H</u> proton) are shown by the numerical figures. Remark: The numbers of the DhThr residues (3 residues) of compound **B** are the same as natural cichorinotoxin (3 residues).

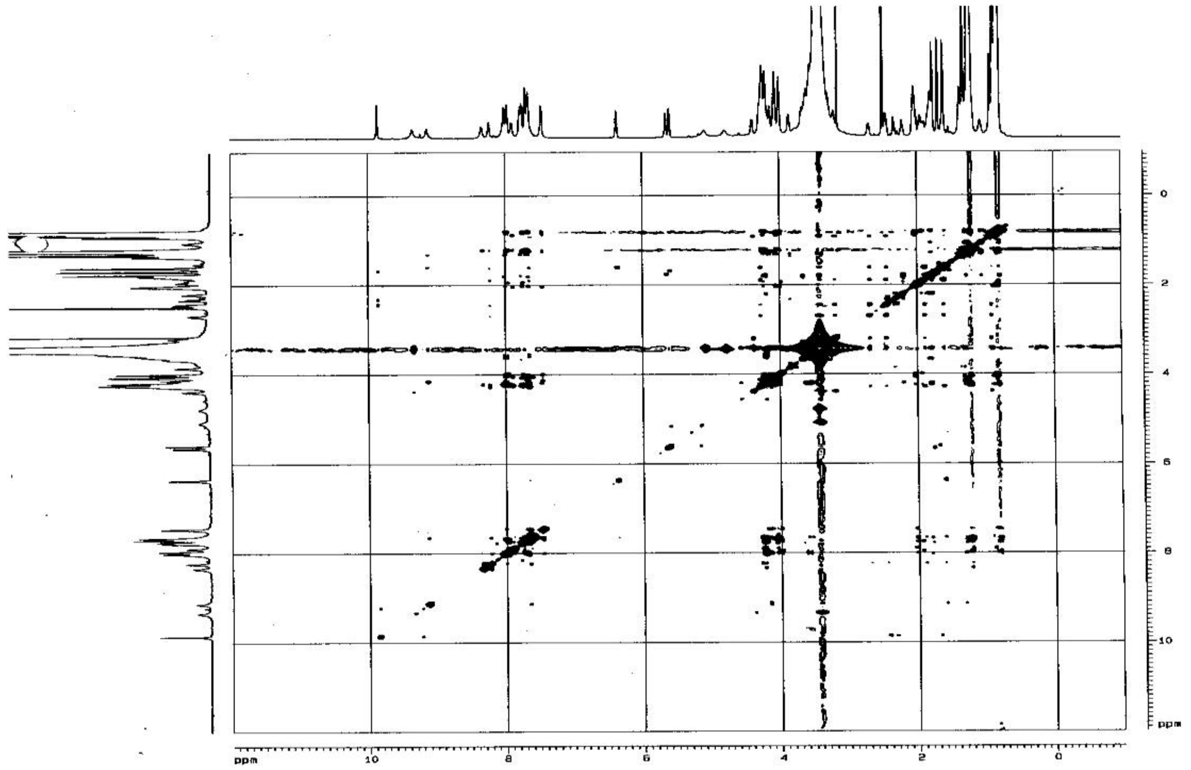

Figure S17C: The overall NOESY spectrum compound **B** (600 MHz, DMSO-d_6).

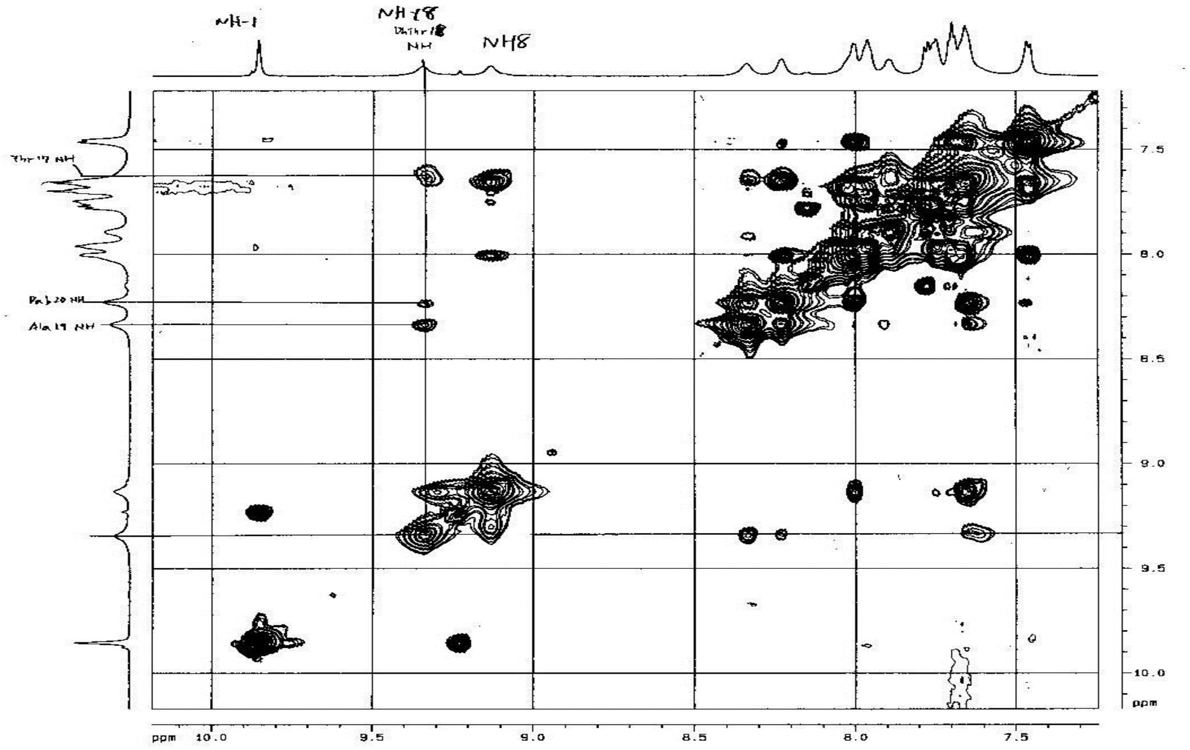

Figure S17D: NOESY correlations for $\alpha N^{i}H/\alpha N^{i+1}H$ (expanded region of Figure S17C).

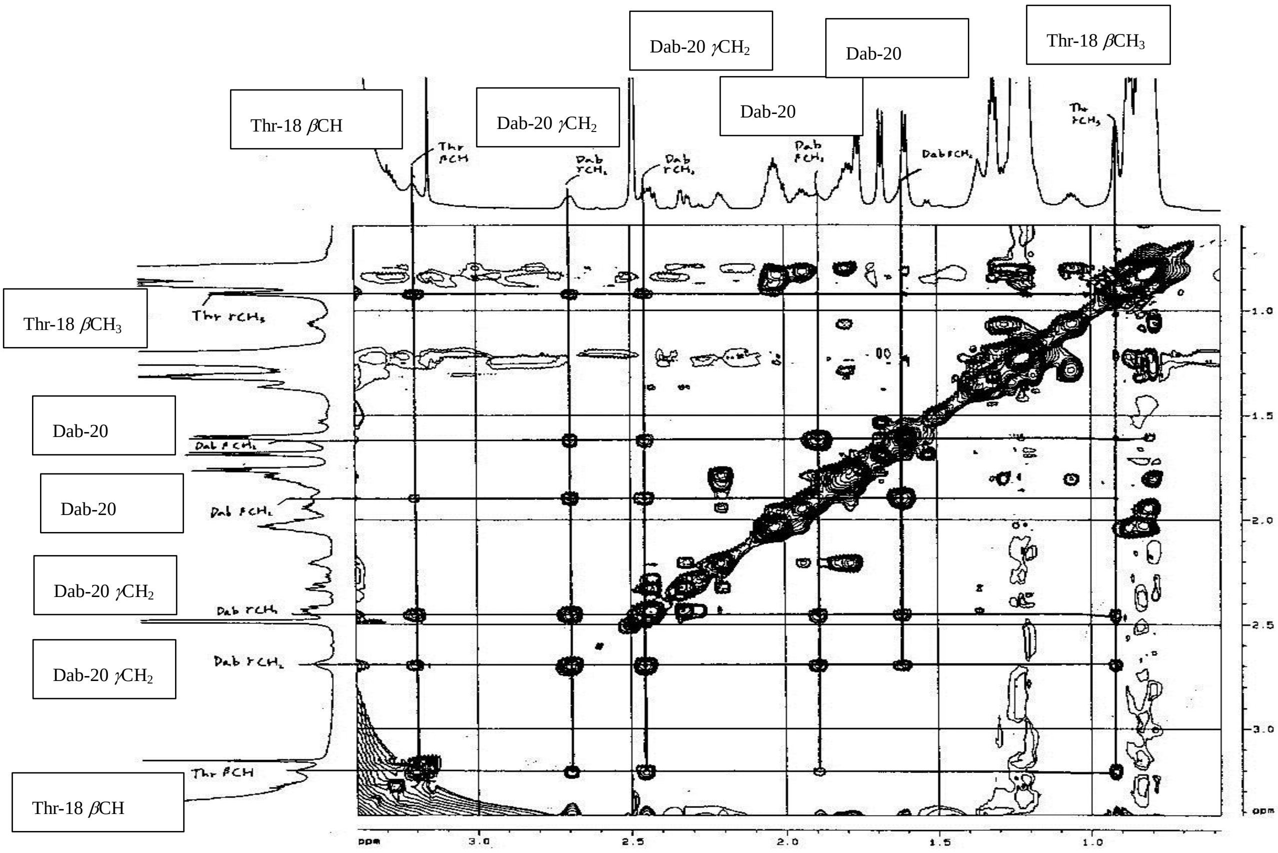

Figure S17E: NOESY correlations highlighted between βC$\underline{H_2}$ of Thr_{17} and γC$\underline{H_2}$ of Dab_{20}.

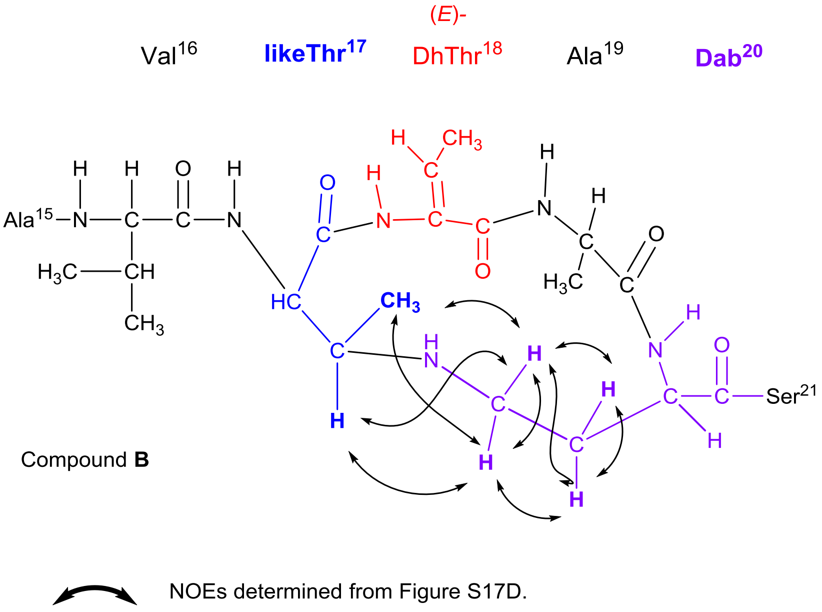

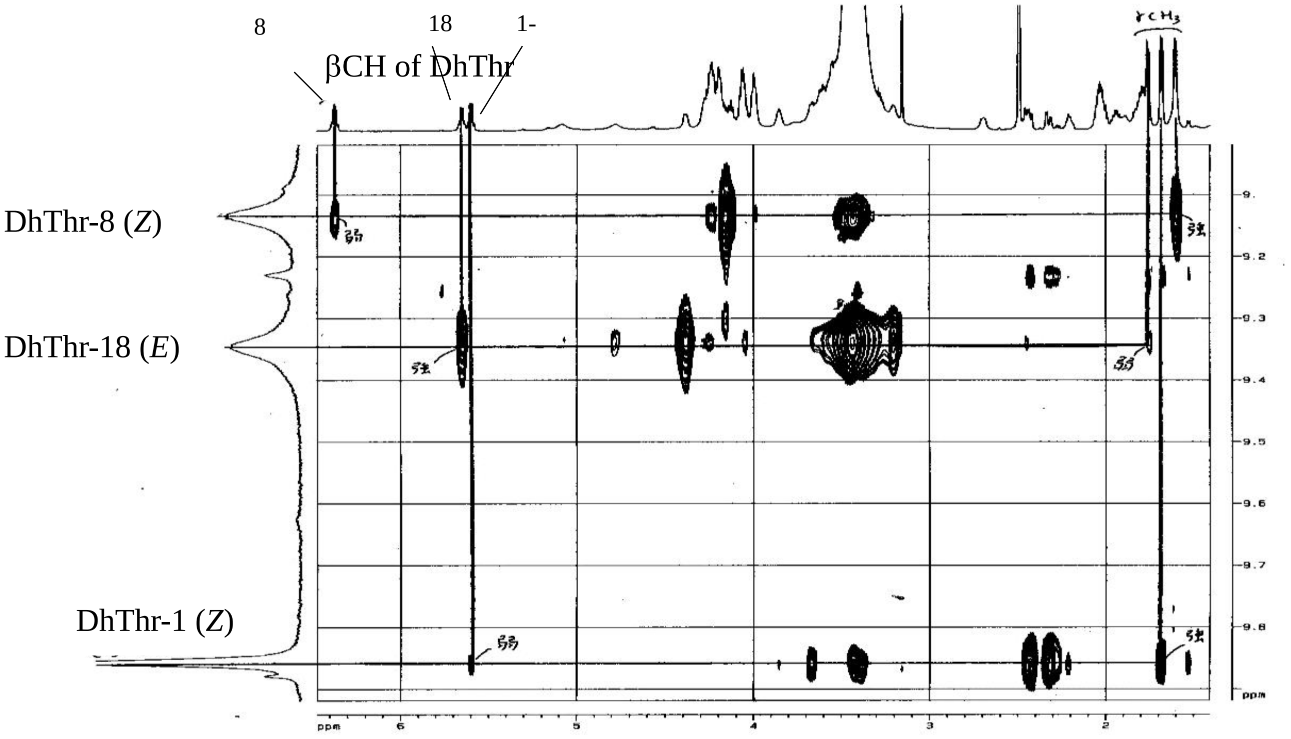

Figure S17F: NOESY spectrum for the determination of *E*,*Z*-configuration of three DhThr residues.

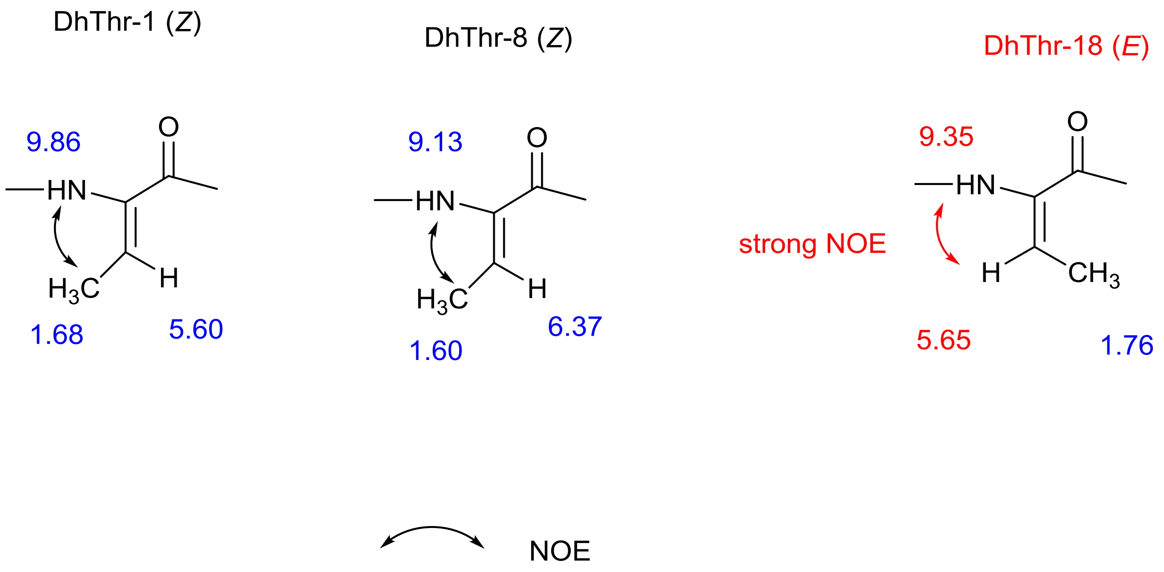

Compound **A**

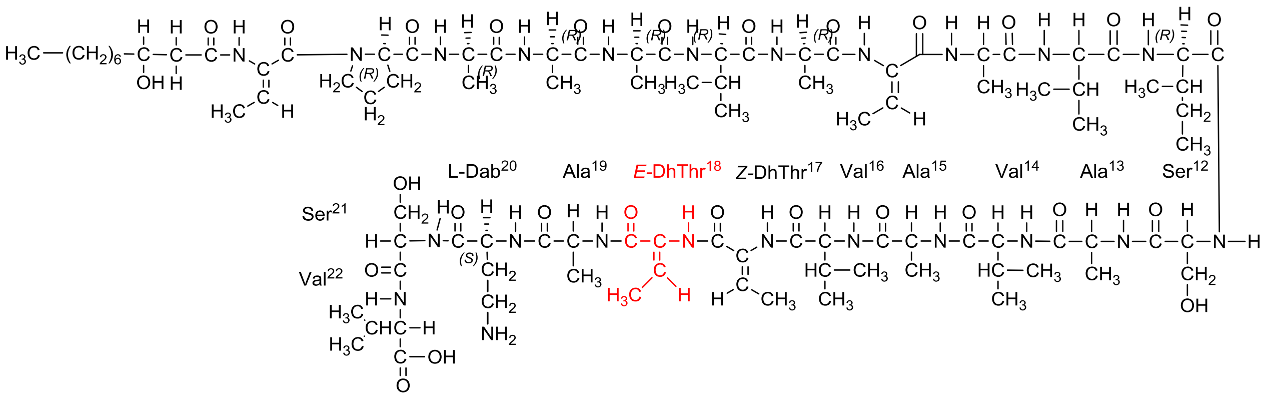

Compound **B**

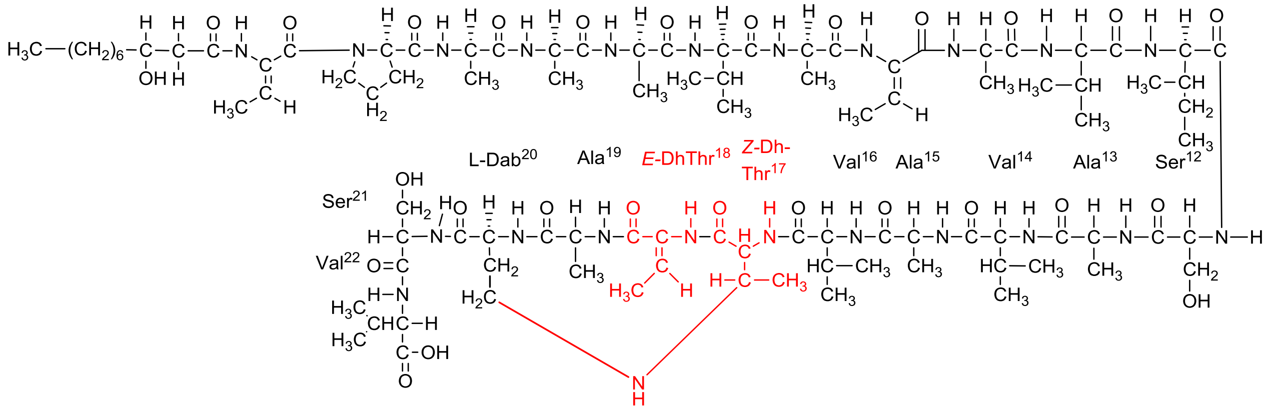

Figure S18: Overall structures of compounds **A** and **B**, which were generated by the alkaline hydrolysis of natural cichorinotoxin.

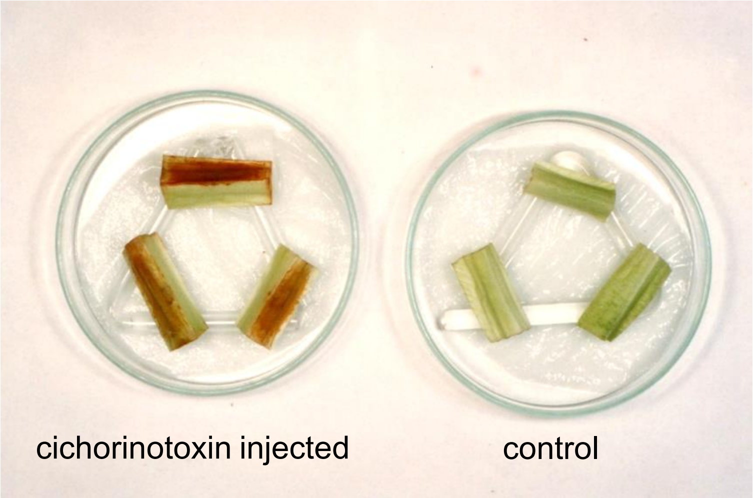

Figure S19A: This photograph shows the bioassay method. Left: cichorinotoxin was injected to the lettuce midrib (just under the epidermis) and stood at ambient temperature for 1 day. A small amount of water was immersed in a filter paper, which was placed below the petri dish. This necrotic lesion appears after standing usually for 1 to 2 days.

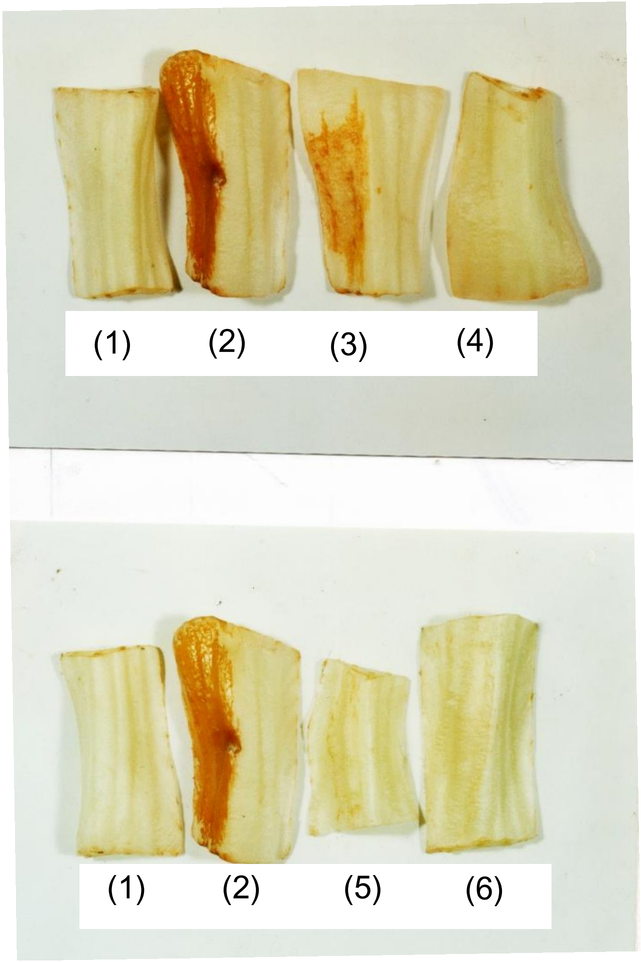

Figure S19B: Necrotic lesions of lettuce, which were caused by the following compounds. (1) Distilled water; (2) cichorinotoxin; (3) mono-acetate; (4) tetra-acetate; (5) compound **A**; (6) compound **B**.

One mg of these compounds (2)–(5) were dissolved in MeOH (100 μl). To the 10 μl of the MeOH solution, 190 μl of a distilled water was added. The suspensions thus obtained were well immingled and 50 μl of this suspension was injected into each midrib of lettuce. The injected quantities were 25 μg for each of compounds (2)–(5). (1) indicates the control, the experiment of which was carried out by no injection of any other compounds (just the distilled H_2O including small amount of MeOH was injected). Necrotic activities caused by compounds (3)–(6) were significantly decreased, therefore these pictures were taken after standing 3 days at 30 °C.

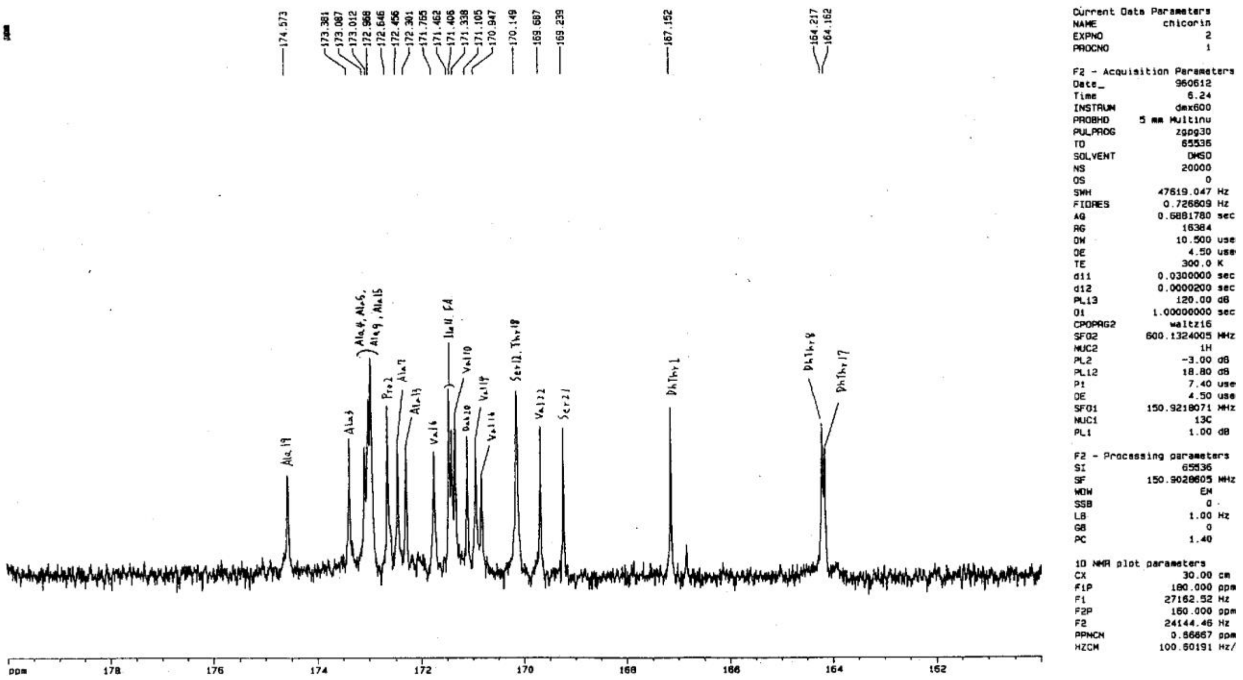

Figure S20A: ^{13}C NMR spectrum of natural cichorinotoxin (150 MHz, DMSO-d_6).

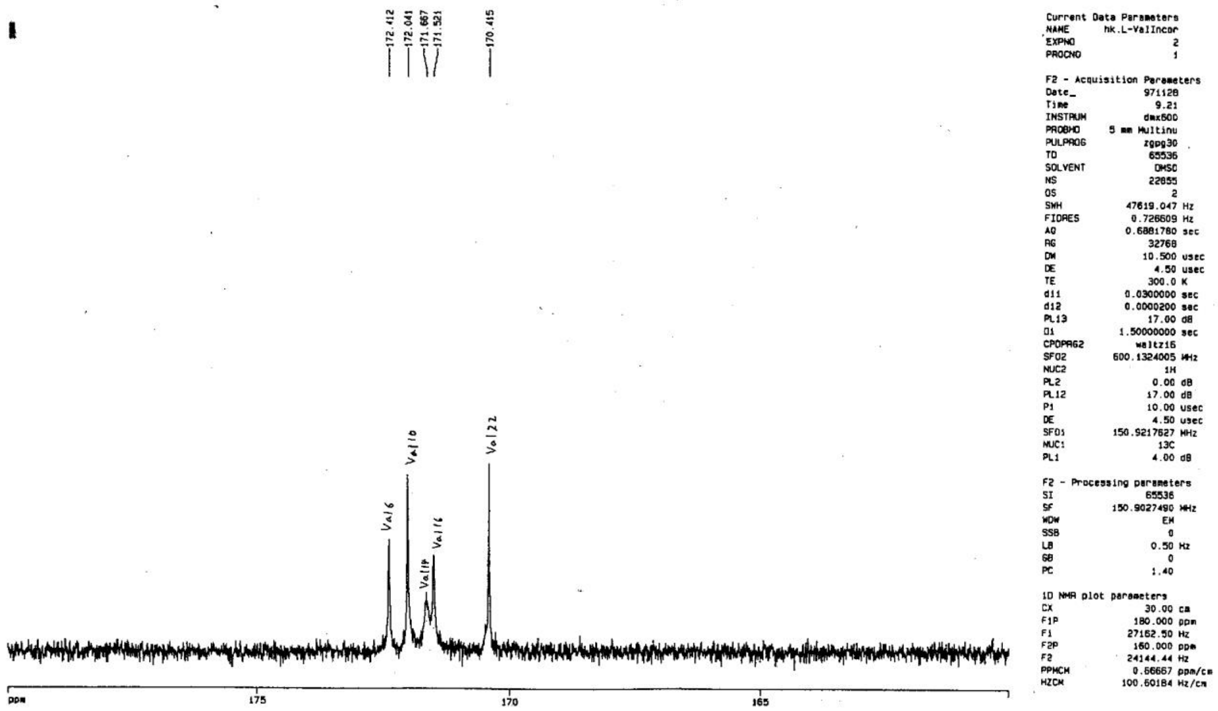

Figure S20B: ^{13}C NMR spectrum of cichorinotoxin (150 MHz, DMSO-d_6), which was obtained by feeding L-[1-^{13}C]-labelled Val.

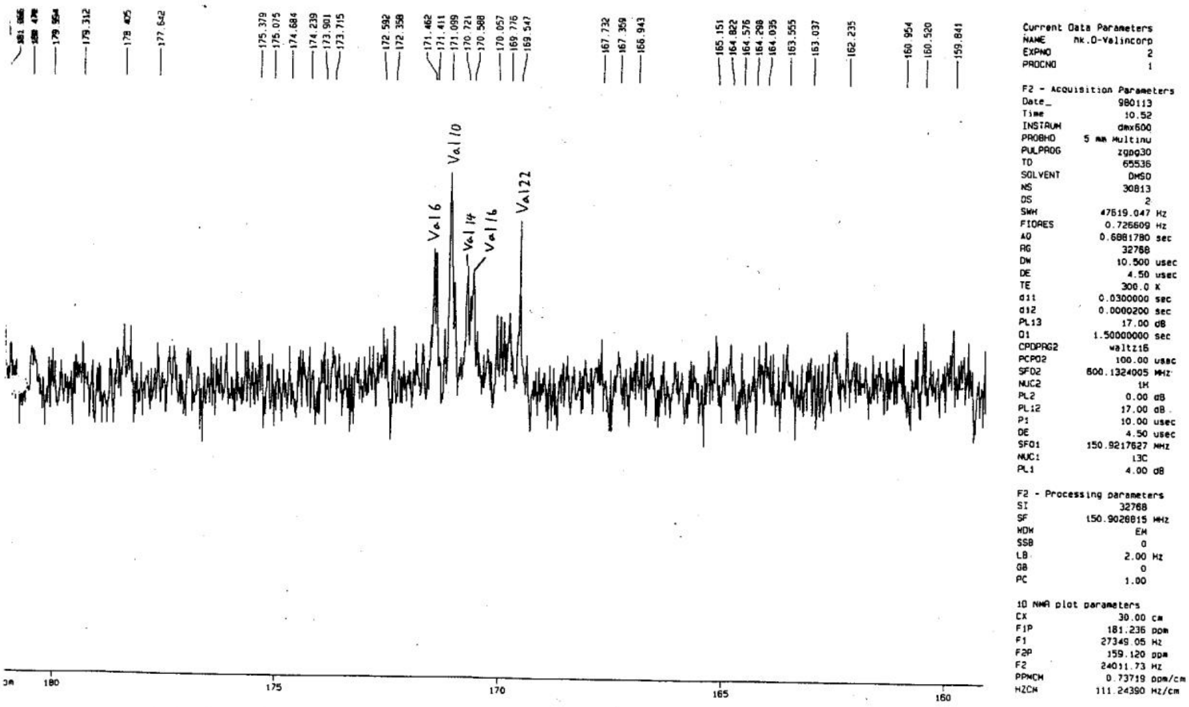

Figure S20C:. ^{13}C NMR spectrum of cichorinotoxin (150 MHz, DMSO-d_6), which was obtained by feeding D-[1-^{13}C]-labelled Val.

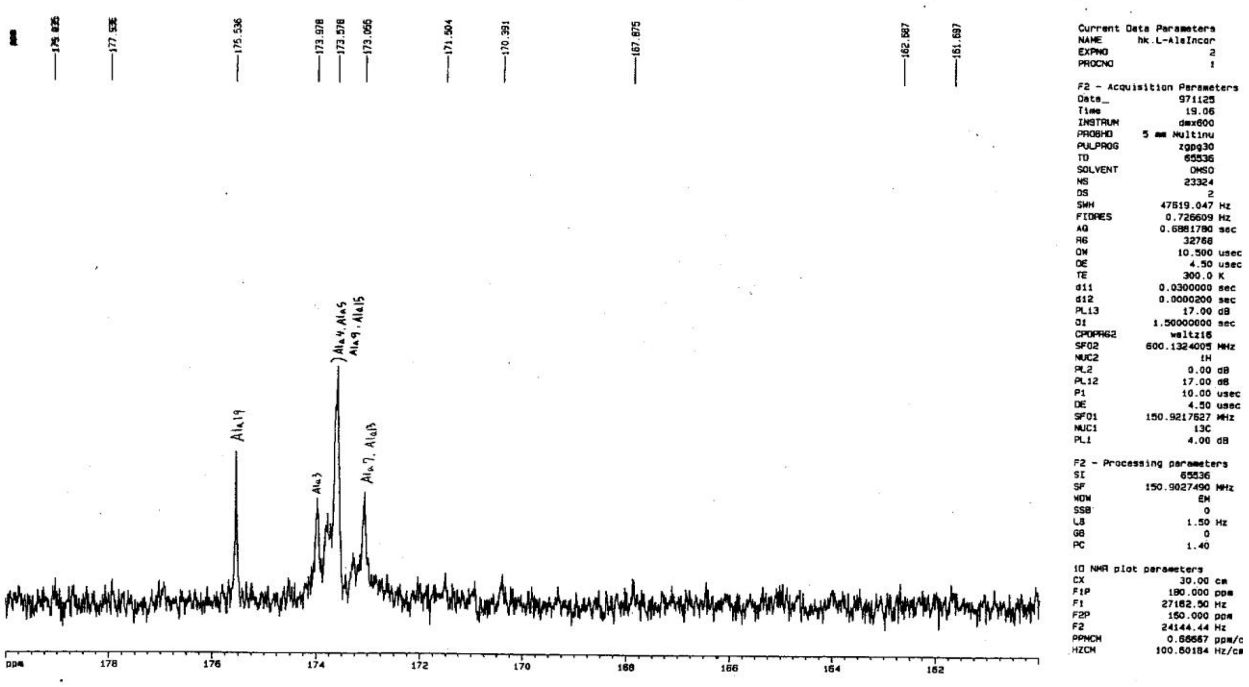

Figure S20D: ^{13}C NMR spectrum of cichorinotoxin (150 MHz, DMSO-d_6), which was obtained by feeding L-[1-^{13}C]-labelled Ala.

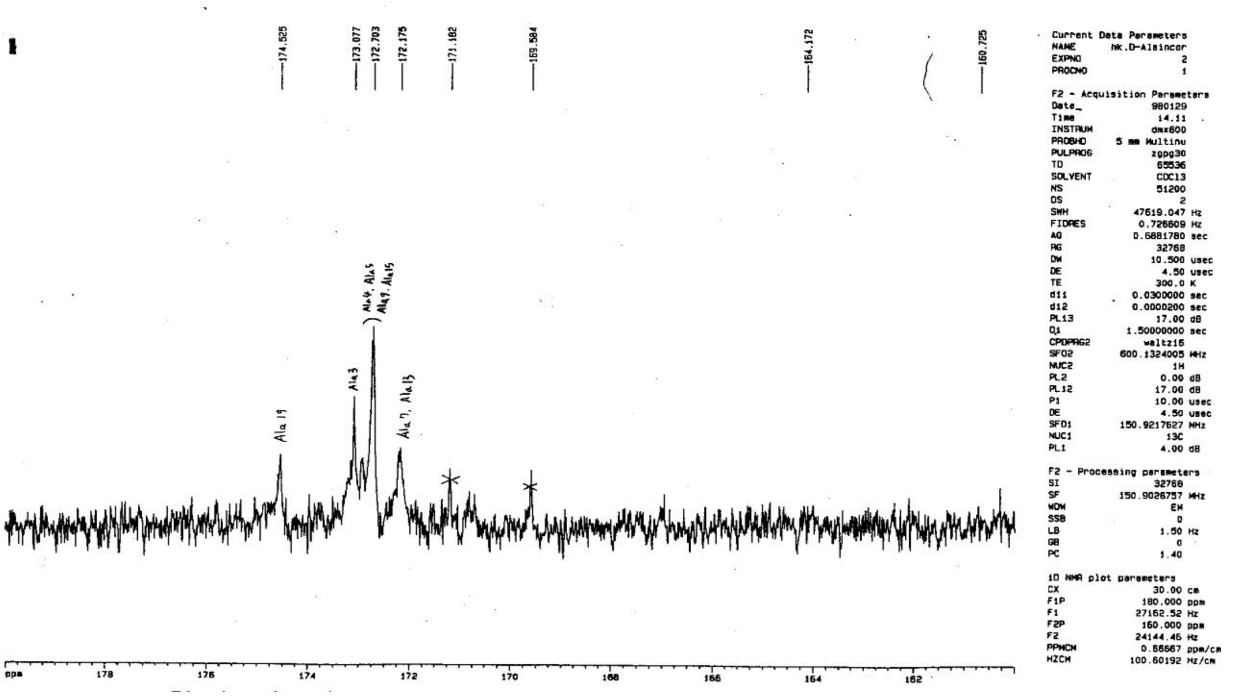

Figure S20E: ^{13}C NMR spectrum of cichorinotoxin (150 MHz, DMSO-d_6), which was obtained by feeding D-[1-^{13}C]-labelled Ala.

Conclusions: Five Val residues (Val^6, Val^{10}, Val^{14}, Val^{16} and Val^{22}) are involved in cichorinotoxin (Figure 6, main Text). The five Val residues are well separated in the ^{13}C NMR spectra (Figures S20A, S20B and S20C). All the five Val residues were labelled by the feedings of L- and D-^{13}C labelled Val amino acids, despite both D- and L- Val residues being involved in cichorinotoxin (see Table 1, main Text). This finding definitively demonstrated that a racemase is included in the NRPS biosynthetic gene cluster.

Figures S20D and 20E indicated that any other amino acids other than Ala were not labelled and that the two spectra showed almost the same pattern. Separate administrations of D- and L-Ala enriched all the signals of amide carbonyl groups of Ala residues (Ala1^9, Ala^3, Ala^4, Ala^5, Ala^9, Ala^{15}, Ala^7 and Ala^{13}), despite both D- and L- Ala residues being involved in cichorinotoxin (Table 1, main Text), suggesting that the epimerase (racemase) are present in the biosynthetic gene cluster.
